# Trash to Treasure: Eco-Friendly and Practical Synthesis of Amides by Nitriles Hydrolysis in WEPPA

**DOI:** 10.3390/molecules24213838

**Published:** 2019-10-24

**Authors:** Yajun Sun, Weiwei Jin, Chenjiang Liu

**Affiliations:** The Key Laboratory of Oil and Gas Fine Chemicals, Ministry of Education & Xinjiang Uygur Autonomous Region, Urumqi Key Laboratory of Green Catalysis and Synthesis Technology, School of Chemistry and Chemical Engineering, Xinjiang University, Urumqi 830046, China; yx1330221804@163.com

**Keywords:** amides, nitrile hydrolysis, agro-waste, green reaction medium

## Abstract

The hydration of nitriles to amides in a water extract of pomelo peel ash (WEPPA) was realized with moderate to excellent yields without using external transition metals, bases or organic solvents. This reaction features a broad substrate scope, wide functional group tolerance, prominent chemoselectivity, and good reusability. Notably, a magnification experiment in this bio-based solvent at 100 mmol further demonstrated its practicability.

## 1. Introduction

**Scheme 1:**
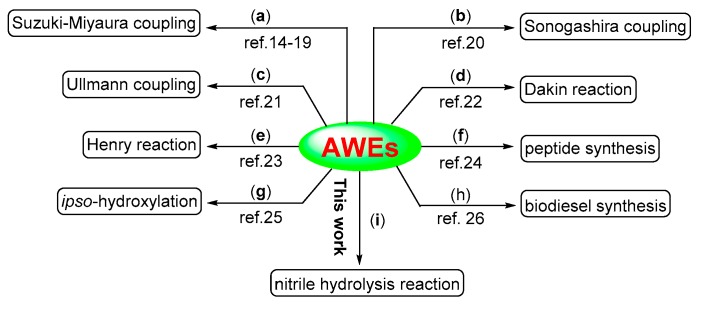
Representative reactions realized in AWEs.

Nowadays, with the aggravation of the environmental crisis, there is an increasing requirement for sustainable chemical technologies from academia and industry. Significant advances have been made in the exploitation of greener processes from renewable feedstocks [1,2,3]. Volatile organic solvents (VOCs) are recognized as one of the major contributors to the generation of bulky chemical waste. A range of green solvents such as ionic liquids [4,5], deep eutectic solvents [6], super critical fluids [7,8,9], and biosolvents [10], fluorinated solvents [11], water [12], and so on have been developed as alternative solutions. In recent years, a water extract of agro-waste ash (AWEs) has emerged as a novel green reaction medium [13] and has been successfully employed in transition-metal-catalyzed cross-coupling reactions (Suzuki–Miyaura [14,15,16,17,18,19], Sonogashira [20], Ullmann [21]), Dakin reaction [22], Henry reaction [23], peptide synthesis [24], ipso-hydroxylation [25], and biodiesel synthesis [26] (Scheme 1a–h)). AWEs, easily prepared from various renewable agricultural waste products, can be a rich source of raw materials and play multiple roles, including those of the water, in situ base, reductant, and so forth.

Amides are an important class of organic synthetic building blocks and have been widely used in the construction of pharmaceutical molecules, in pesticide chemistry, and as advanced functional materials [27,28,29]. Among the well-established synthetic methods for amide synthesis, nitrile hydration reactions are considered one of the most straightforward and economic. For this purpose, some elegant synthetic methods involving the hydration of nitriles and employing transition metal catalysts (e.g., Ru [30,31,32,33,34,35], Rh [36,37], Pd [38,39], Os [40], Ir [41], Pt [42], Cu [43,44,45], Ag [46,47], Au [48,49], Fe [50], Co [51], Ni [52,53], Mn [54,55], etc.) have been well documented. Meanwhile, some alternative methods for the nitriles’ hydration reactions with transition-metal-free catalysts such as acids [56,57,58], bases [59,60,61,62,63,64,65], and others [66,67] have also been developed. These protocols usually have some inherent drawbacks. Some nitrile hydration reactions in aqueous media have been well documented, but transition metal catalysts, external strong acids, strong bases, and/or volatile organic solvents are usually indispensable in these transformations [30,31,32,33,34,35,36,37,38,39,40,41,42,43,44,45,46,47,48,49,50,51,52,53,54,55,56,57,58,59,60,61,62,63,64,65].

As a result, developing a biocompatible, recyclable, and practical procedure for the construction of amides is still highly desirable. In light of Green Chemistry Principles 5 and 7 [68], and also as part of our long-term pursuit of environmentally benign chemistry [69,70,71,72], we present herein our systematic studies on the preparation and characterization of several kinds of water extract of agro-waste ash and their performance in the multiple roles of base, solvent, and promoter in the hydration of nitriles to amides (Scheme 1i). To the best of our knowledge, this is the first example of the formation of valuable amide derivatives through the hydrolysis of nitriles using a water extract of agro-waste ash as the green reaction medium.

## 2. Results and Discussion

**Figure 1:**
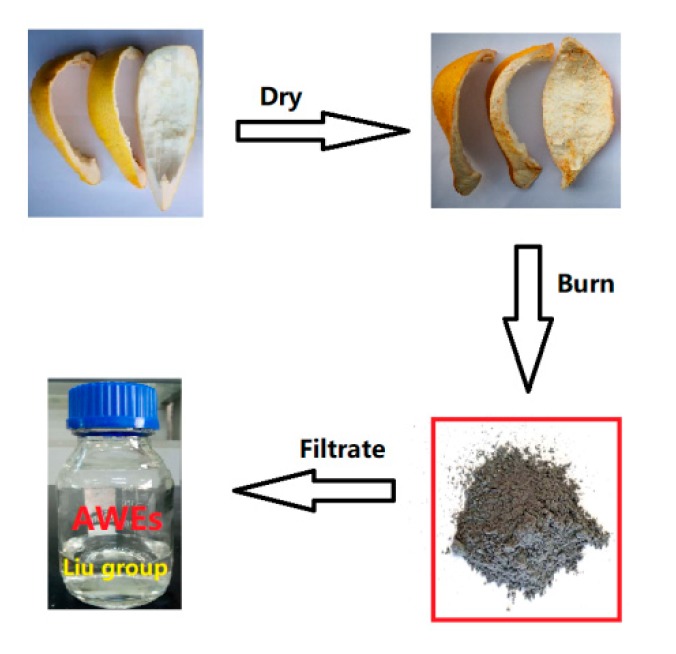
Preparation of AWEs.

**Figure 2:**
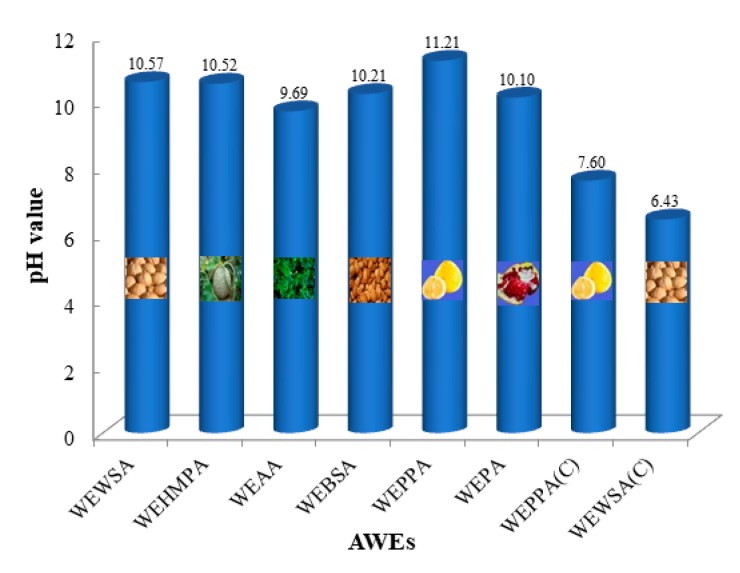
pH values of AWEs.

The AWEs used in this paper were prepared according to the literature methods [13,14,15,16,17,18,19,20,21,22,23,24,25,26]: (i) drying the agro-waste; (ii) burning the dried agro-waste to get the ash; (iii) suspension and stirring of the ash in distilled water, followed by filtration with sintered glass crucible and collection filtrate (Figure 1). For a comparison with the reported methods, we prepared two kinds of AWEs using the ash obtained by high-temperature calcination and marked them as WEPPA(C) and WEWSA(C). The pH values of AWEs were measured by the pH meter, as shown in Figure 2. Among them, WEPPA had the highest basicity (pH = 11.21), while high-temperature calcination led to an obvious decrease in basicity (pH: 10.57 versus 6.43, 11.21 versus 7.60).

**Figure 3:**
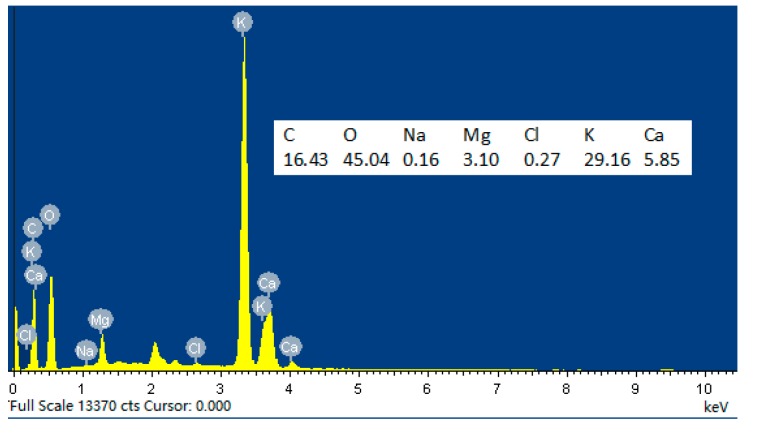
EDX spectrum of the pomelo peel ash.

**Figure 4:**
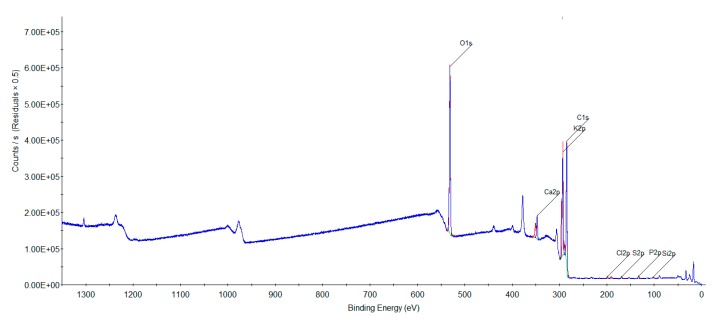
XPS spectrum of the pomelo peel ash.

To illustrate the origin of the basicity and analyze the kinds and concentrations of the remaining elements, the pomelo peel ash was characterized by inductively coupled plasma atomic emission spectroscopy (ICP-AES) (Table 1), energy-dispersive X-ray (EDX) (Figure 3), and X-ray photoelectron spectroscopy (XPS) (Figure 4 and Table 2, respectively. The high element concentrations of K, Ca, Mg, and Na were revealed by the ICP-AES analysis. That was why the aqueous pomelo peel ash had strong basicity. The EDX and XPS spectrums jointly revealed an abundance of the oxides and/or carbonates of K, Ca, Mg, and Na.

The hydration of benzonitrile (**1a**) to benzamide (**2a**) was selected as the model reaction to optimize the reaction conditions (Table 3). Among a range of AWEs screened, WEPPA produced the best results and produced benzamide in a 41% conversion (entries 1–6). This result could be ascribed to the strongest basicity of WEPPA. Notably, the different preparation methods of AWEs were critical for the efficient hydration of the substrate, and the results were consistent with their relative weakly acidity or alkalinity (entries 7–8, pH = 6.43 or 7.60). Extending the reaction time to 24 or 36 h had only limited effects on the conversion (entries 9–10), while the conversions could be remarkably improved by increasing the reaction temperature (entries 11–13). To our delight, the reaction efficiency could be further boosted when performed the model reaction in the closed vessel synthesis reactor (entries 14–16). The best isolated yield, 94%, was obtained at 150 °C for 0.5 h (entry 17). Reducing the reaction time had a negative impact on the reaction activity (entry 18). A control experiment showed that WEPPA was essential; no reaction was happened in distilled water (entry 19).

Having established the optimized reaction conditions in hand, the versatility of this protocol was then exploited (Table 4). Generally, various functional benzonitriles with electron-donating or -withdrawing groups reacted smoothly to generate the desired amide products **2a**–**y** in moderate to excellent yields (41–96%). The steric hindrance of the methyl group on the phenyl ring had little influence on the isolated yields (**2b**–**d**). The condensed aromatic nitriles could also be converted into the corresponding products **2m** and **2n** in moderate yields. Aminobenzamide derivatives, especially *o*-aminobenzamides, which are important synthetic structural units, could be conveniently prepared with 79–96% yields (**2o**–**v**). Isophthalamide (**2w**) and terephthalamide (**2x**) were obtained by the concurrent hydrolysis of two cyano groups of phthalonitriles. The total chemoselectivity of this catalytic system was verified by the hydrolysis of starting material **1y** containing both the aromatic and aliphatic cyano groups, only the aromatic cyano group was transformed to the amide group with a 77% yield. In a similar fashion, *trans*-cinnamonitrile and ferroceneactonitrile underwent an efficient hydrolysis reaction, producing the desired *trans*-cinnamamide (**2z**) and ferrocenecarboxamide (**2aa**) with good yields (84% and 63%, respectively). This methodology could also be extended to the heteroaromatic nitriles including five- and six- membered *N*-, *O*-, and *S*-containing heterocycles (**2a′**–**g′**). To our delight, aliphatic nitriles could also be efficiently involved in this catalytic system (**2a″**–**d″**).

**Scheme 2:**
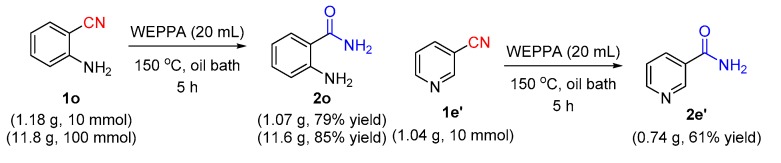
Gram-scale reactions of **1o** and **1e′**.

The potential and practical applications of this environmentally benign protocol were firmly demonstrated by the scaling-up experiments of **1o** and **1e′** at 10 mmol; the desired hydration products **2o** and **2e′** were obtained with 79% and 61% yields, respectively. Notably, the hydration of *o*-aminobenzonitrile was further amplified to 100 mmol to assemble the *o*-aminobenzoamide **2o** in 85% yield (Scheme 2). The synthetic significance of amides was confirmed by the transformation of aromatic amides to useful synthetic intermediates. With Lawesson reagent as the thionation reagent, benzothioamide **3a** was readily obtained with a 73% yield [73] (Equation (1)). Based on a consecutive iodination/Kornblum oxidation/annulation tandem reaction, 2-aminobenzamide (**2o**) was transformed to the heterocyclic product 2-benzoylquinazolin-4(3*H*)-one **3c** (75%), which was an analogue of alkaloid Luotonin F [74] (Equation (2)).
(1)
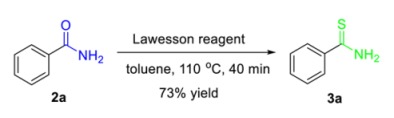

(2)
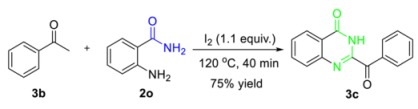
.



**Figure 5:**
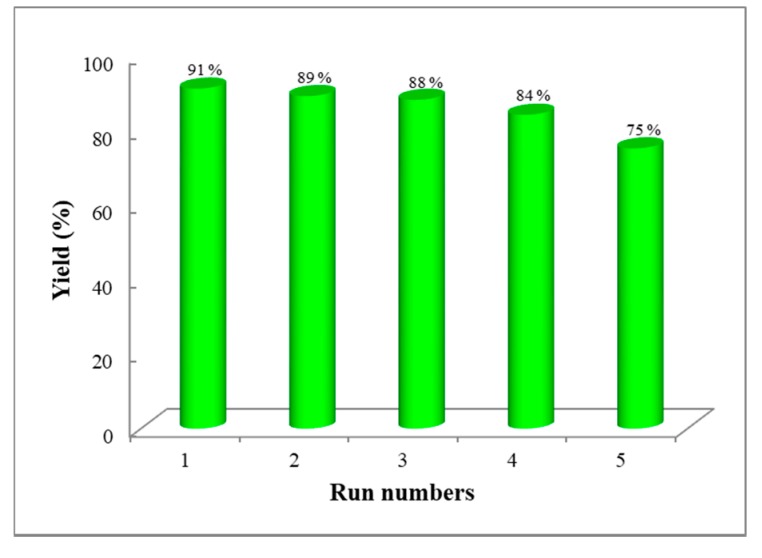
Recycling use of the WEPPA in the synthesis of 4-fluorobenzamide **2g**.

It is worth noting that the AWEs could be easily recycled. The good reusability performance of WEPPA was evaluated in the hydration of 4-fluorobenzonitrile **1g** under optimal conditions. WEPPA could be reused at least four times with good yields (Figure 5). Interestingly, the target product 4-fluorobenzamide **2g** was a water-insoluble white solid and WEPPA could be easily separated by simple filtering with a sintered glass crucible without further purification by column chromatography. Then the recovered WEPPA filtrate could be reused for the subsequent hydrolysis reaction. Actually, the reduction of catalytic efficiency was largely due to the gradual volumetric loss of WEPPA during the product purification process.

In order to probe the real role of the water extract of agro-waste ash, some comparative experiments were designed and conducted (Table 5). A series of water solutions were prepared by dissolving commercially available inorganic carbonates or oxides of K, Na, Ca, Mg, Cu, Fe, and Mn in distilled water. The dosage of carbonates or oxides was based on the results of the ICP-AES analysis of the pomelo peel ash. Under standard conditions, not all of them could give similar results to WEPPA; the water solution of CaO produced the highest yield of the target product **2a** in 40% (Table 5, entry 7). Even the water solution that combined these inorganic carbonates and oxides only produced **2a** with a 26% yield (Table 5, entry 12). Although the real role of the water extract of agro-waste ash is not clear at present, these preliminary results demonstrated that it may not be functioning as the green reaction medium and base. 

The concrete mechanism was also not clear. Based on literature reports and the primary results mentioned above, we thought the mixture materials showed a synergistic effect [9e], which is obviously different from the hydrolysis reaction in a water solution of single inorganic carbonates or oxides (for example, K_2_CO_3_, Na_2_CO_3_, etc.) and produced the result of “a whole greater than the sum of the parts.”

## 3. Materials and Methods

### 3.1. General Experimental Procedures

^1^H-, ^13^C-, and ^19^F-NMR spectra were recorded on a Varian Inova-400 (400 MHz, 100 MHz and 376 MHz, respectively) spectrometer (Varian, Palo Alto, CA, USA). ^1^H- and ^13^C-NMR chemical shifts were determined relative to internal standard TMS at δ 0.0 or CDCl_3_ (δ(^1^H), 7.26 ppm; δ(^13^C), 77.16 ppm) or *d*_6_-DMSO (δ(^1^H), 2.54 ppm; δ(^13^C), 39.50 ppm), and ^19^F NMR chemical shifts were determined relative to CFCl_3_ as internal standard. Chemical shifts (δ) are reported in ppm, and coupling constants (*J*) are in hertz (Hz). The following abbreviations are used to explain the multiplicities: s = singlet, d = doublet, t = triplet, q = quartet, m = multiplet, bs = broad singlet. pH values were detected by a PHS-3C acidometer (Rex Electric Chemical, Shanghai, China). Inductively coupled plasma atomic emission spectroscopy (ICP-AES) analysis was carried out on a Varian VISTA-PRO spectrometer (Varian, Palo Alto, CA, USA). X-ray photoelectron spectroscopy (XPS) was detected on a Thermo Scientific K-Alpha+X spectrometer (Thermo Fisher Scientific, Waltham, MA, USA). Energy-dispersive X-rays (EDX) were recorded on the SU8010 cold field emission ultra-high-resolution scanning electron microscope (Carl Zeiss AG, Jena, Germany). The melting point was recorded on BÜCHI (M-560) (WoLong Instrument, Shanghai, China) and uncorrected. Analytical thin-layer chromatography (TLC) was performed on 0.25 mm silica gel 60 F254 plates and viewed by UV light (254 nm). Column chromatographic purification was performed using a 200–300 mesh silica gel. All the chemical reagents were purchased from commercial sources and used as received unless otherwise indicated.

### 3.2. General Procedure for the Preparation of AWEs (Taking WEPPA as an Example)

The pomelo peel was obtained and dried naturally. The dried pomelo peel was burned to get its ash. Then, one gram pomelo peel ash was suspended into 10.0 mL of distilled water at room temperature for 30 min with constant stiring. The suspension was then filtered to obtain a pale yellow extract which named as WEPPA. 

### 3.3. General Procedure for the Hydrolysis of Nitriles in WEPPA (Taking ***1a*** as an Example)


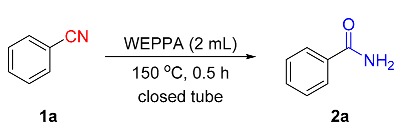


Benzonitrile **1a** (103 mg, 1.0 mmol) and WEPPA (2.0 mL) were added into a 10-mL closed tube with a stir bar. Then the reaction was stirred in a closed vessel synthesis reactor at 150 °C for 0.5 h. After cooling to ambient temperature, the resulting precipitate was collected by filtration, washed with ice water, and further dried in a vacuum drying oven. The filtrate was evaporated under reduced pressure. The resultant residue was purified by silica gel column chromatography (eluent: petroleum ether (35–60 °C)/EtOAc = 2:1 to 0:1, *v/v*). Finally, these two parts were combined to produce the desired benzamide **2a** with a 94% yield. *Benzamide* (**2a**): Known compound. 114.2 mg, 94% yield. White solid. m.p.: 127.3–129.1 °C. ^1^H-NMR (CDCl_3_, 400 MHz) δ 7.83–7.80 (m, 2H), 7.54–7.50 (m, 1H), 7.46–7.42 (m, 2H), 6.26 (bs, 2H); ^13^C-NMR (CDCl_3_, 100 MHz) δ 169.7, 133.5, 132.1, 128.8, 127.5.

### 3.4. Gram-Scale Experiments (Taking ***1o*** at 100 mmol as an Example)


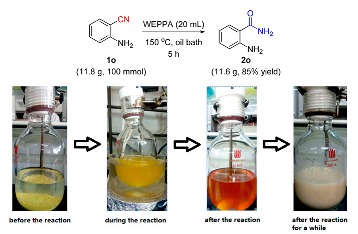


2-Aminobenzonitrile **1o** (11.8 g, 100.0 mmol) and WEPPA (150.0 mL) were added into a 300 mL closed tube with a stir bar. Then the reaction was stirred in an oil bath at 150 °C for 5 h. After cooling to ambient temperature, large amount of white solid precipitated out and was collected by filtration, washed with ice water and further dried in the vacuum drying oven. The filtrate was evaporated under reduced pressure to get the residual product. Finally, cmbining these two parts to afford the desired 2-aminobenzamide **2o** (11.6 g) in 85% yield. 

### 3.5. Recycling Experiments


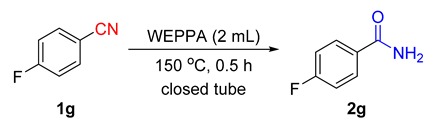


4-Fluorobenzamide **1g** (121 mg, 1.0 mmol) and WEPPA (2.0 mL) were added into a 10 mL closed tube with a stir bar. Then the reaction was stirred in a closed vessel synthesis reactor at 150 °C for 0.5 h. After cooling to ambient temperature, the resulting precipitate was collected by filtration, washed with ice water and further dried in the vacuum drying oven. The WEPPA filtrate could be reused at least four times in good yields (89%, 88%, 84% and 75%). 

### 3.6. Comparative Experiments

The results are summarized in Table 4. All products are known compounds, and were characterized by their ^1^H- and ^13^C-NMR spectra (see the Supplementary Materials).

*2-Methylbenzamide* (**2b**): Known compound. 114.1 mg, 84% yield. White solid. m.p.: 140.1–142.9 °C. ^1^H-NMR (CDCl_3_, 400 MHz) δ 7.43 (d, *J* = 7.6 Hz, 1H), 7.32 (td, *J* = 7.6 and 1.3 Hz, 1H), 7.21 (q, *J* = 7.2 Hz, 2H), 6.28 (bs, 1H), 5.86 (bs, 1H), 2.49 (s, 3H); ^13^C-NMR (CDCl_3_, 100 MHz) δ 172.4, 136.4, 135.4, 131.3, 130.4, 127.1, 125.8, 20.1.

*3-Methylbenzamide* (**2c**): Known compound. 108.2 mg, 80% yield. White solid. m.p.: 90.1–91.1 °C. ^1^H-NMR (CDCl_3_, 400 MHz) δ 7.65 (s, 1H), 7.60–7.58 (m, 1H), 7.33–7.29 (m, 2H), 6.30 (bs, 2H), 2.39 (s, 3H); ^13^C-NMR (CDCl_3_, 100 MHz) δ 170.0, 138.6, 133.5, 132.8, 128.6, 128.2, 124.4, 21.4. 

*4-Methylbenzamide* (**2d**): Known compound. 120.2 mg, 89% yield. White solid. m.p.: 148.1–148.8 °C. ^1^H-NMR (CDCl_3_, 400 MHz) δ 7.71 (d, *J* = 8.2 Hz, 2H), 7.25 (d, *J* = 9.0 Hz, 2H), 5.93 (bs, 2H), 2.42 (s, 3H); ^13^C-NMR (CDCl_3_, 100 MHz) δ 169.5, 142.7, 130.6, 129.4, 127.5, 21.6.

*4-Ethylbenzamide* (**2e**): Known compound. 132.6 mg, 89% yield. White solid. m.p.: 160.2–162.5 °C. ^1^H-NMR (CDCl_3_, 400 MHz) δ 7.74 (d, *J* = 8.3 Hz, 2H), 7.27 (d, *J* = 8.3 Hz, 2H), 6.06 (bs, 2H), 2.70 (q, *J* = 7.6 Hz, 2H), 1.25 (t, *J* = 7.6 Hz, 3H); ^13^C-NMR (CDCl_3_, 100 MHz) δ 169.7, 148.8, 130.9, 128.2, 127.6, 28.9, 15.4.

*4-(Chloromethyl)benzamide* (**2f**): Known compound. 107.2 mg, 63% yield. White solid. m.p.: 133.3–135.1 °C. ^1^H-NMR (*d*_6_-DMSO, 400 MHz) δ 7.92 (bs, 1H), 7.84 (d, *J* = 8.2 Hz, 2H), 7.38 (d, *J* = 8.0 Hz, 2H), 7.30 (bs, 1H), 4.56 (s, 2H); ^13^C-NMR (*d*_6_-DMSO, 100 MHz) δ 167.8, 145.9, 132.6, 127.3, 125.9, 62.5.

*4-Fluorobenzamide* (**2g**): Known compound. 127.0 mg, 91% yield. White solid. m.p.: 155.3–155.5 °C. ^1^H-NMR (*d*_6_-DMSO, 400 MHz) δ 8.03 (bs, 1H), 8.00–7.96 (m, 2H), 7.43 (bs, 1H), 7.34–7.28 (m, 2H); ^13^C-NMR (*d*_6_-DMSO, 100 MHz) δ 166.8, 163.9 (d, *J* = 245.8 Hz), 130.7 (d, *J* = 11.5 Hz), 130.1 (d, *J* = 9.0 Hz), 115.1 (d, *J* = 21.6 Hz); ^19^F NMR (*d*_6_-DMSO, 376 MHz) δ −109.6.

*4-Chlorobenzamide* (**2h**): Known compound. 130.1 mg, 84% yield. White solid. m.p.: 177.4–178.8 °C. ^1^H-NMR (CDCl_3_, 400 MHz) δ 7.77–7.74 (m, 2H), 7.45–7.42 (m, 2H), 5.85 (bs, 2H); ^13^C-NMR (CDCl_3_, 100 MHz) δ 168.3, 138.5, 131.8, 129.1, 128.9.

*4-Bromobenzamide* (**2i**): Known compound. 165.0 mg, 83% yield. White solid. m.p.: 188.9–191.6 °C. ^1^H-NMR (*d*_6_-DMSO, 400 MHz) δ 8.08 (bs, 1H), 7.87–7.84 (m, 2H), 7.72–7.68 (m, 2H), 7.49 (bs, 1H); ^13^C-NMR (*d*_6_-DMSO, 100 MHz) δ 166.9, 133.4, 131.2, 129.6, 125.0.

*4-Formylbenzamide* (**2j**): Known compound. 91.0 mg, 61% yield. White solid. m.p.: 178.9–182.1 °C. ^1^H-NMR (*d*_6_-DMSO, 400 MHz) δ 10.09 (bs, 1H), 8.19 (bs, 1H), 8.07 (d, *J* = 7.9 Hz, 2H), 7.99 (d, *J* = 8.0 Hz, 2H), 7.62 (bs, 1H); ^13^C-NMR (*d*_6_-DMSO, 100 MHz) δ 192.9, 167.0, 139.3, 137.8, 129.3, 128.1.

*4-Acetylbenzamide* (**2k**): Known compound. 110.8 mg, 68% yield. Yellow solid. m.p.: 192.5–194.1 °C. ^1^H-NMR (*d*_6_-DMSO, 400 MHz) δ 8.15 (bs, 1H), 8.04–7.98 (m, 4H), 7.57 (bs, 1H), 2.62 (s, 3H); ^13^C-NMR (*d*_6_-DMSO, 100 MHz) δ 197.7, 167.1, 138.6, 138.1, 128.1, 127.7, 26.9.

*[1,1′-Biphenyl]-4-carboxamide* (**2l**): Known compound. 164.1 mg, 83% yield. White solid. m.p.: 232.1–234.5 °C. ^1^H-NMR (*d*_6_-DMSO, 400 MHz) δ 8.08 (bs, 1H), 8.02 (d, *J* = 8.4 Hz, 2H), 7.80 (bs, 1H), 7.78–7.75 (m, 3H), 7.53 (t, *J* = 7.3 Hz, 2H), 7.44 (t, *J* = 7.2 Hz, 2H); ^13^C-NMR (*d*_6_-DMSO, 100 MHz) δ 167.5, 142.7, 139.2, 133.1, 129.0, 128.1, 128.0, 126.8, 126.4.

*1-Naphthamide* (**2m**): Known compound. 94.6 mg, 55% yield. White solid. m.p.: 204.8–206.2 °C. ^1^H-NMR (*d*_6_-DMSO, 400 MHz) δ 8.36 (d, *J* = 7.3 Hz, 1H), 8.05–8.00 (m, 3H), 7.70–7.55 (m, 5H); ^13^C-NMR (*d*_6_-DMSO, 100 MHz) δ 170.5, 134.6, 133.2, 129.7 × 2, 128.1, 126.6, 126.1, 125.6, 125.1, 124.9. 

*Anthracene-9-carboxamide* (**2n**): Known compound. 90.7 mg, 41% yield. Yellow solid. m.p.: 186.2–188.6 °C. ^1^H-NMR (*d*_6_-DMSO, 400 MHz) δ 8.68 (bs, 1H), 8.30 (bs, 1H), 8.16 (d, *J* = 7.9 Hz, 2H), 8.08 (d, *J* = 8.8 Hz, 3H), 7.64–7.57 (m, 4H); ^13^C-NMR (*d*_6_-DMSO, 100 MHz) δ 170.2, 133.7, 130.7, 128.3, 126.8 × 2, 126.2, 125.5, 125.4.

*2-Aminobenzamide* (**2o**): Known compound. 130.5 mg, 96% yield. Yellow solid. m.p.: 110.1–111.5 °C. ^1^H-NMR (CDCl_3_, 400 MHz) δ 7.36 (dd, *J* = 7.9 and 1.3 Hz, 1H), 7.25–7.20 (m, 1H), 6.68 (d, *J* = 8.2 Hz, 1H), 6.66–6.62 (m, 1H), 5.90 (bs, 2H), 5.67 (bs, 2H); ^13^C-NMR (CDCl_3_, 100 MHz) δ 171.8, 149.6, 133.1, 128.1, 117.6, 116.5, 114.1.

*2-Amino-6-methylbenzamide* (**2p**): Known compound. 139.9 mg, 93% yield. White solid. m.p.: 143.7–144.8 °C. ^1^H-NMR (*d*_6_-DMSO, 400 MHz) δ 7.63 (bs, 1H), 7.42 (bs, 1H), 6.92 (t, *J* = 7.7 Hz, 1H), 6.51 (d, *J* = 7.9 Hz, 1H), 6.39 (d, *J* = 7.2 Hz, 1H), 4.90 (bs, 2H), 2.21 (s, 3H); ^13^C-NMR (*d*_6_-DMSO, 100 MHz) δ 170.5, 145.4, 134.2, 128.7, 123.0, 117.9, 112.7, 19.9.

*2-Amino-5-methylbenzamide* (**2q**): Known compound. 135.9 mg, 90% yield. Yellow solid. m.p.: 172.6–174.3 °C. ^1^H-NMR (*d*_6_-DMSO, 400 MHz) δ 7.65 (bs, 1H), 7.34 (bs, 1H), 6.95 (dd, *J* = 8.2 and 1.5 Hz, 2H), 6.59 (d, *J* = 8.2 Hz, 1H), 6.31 (bs, 2H), 2.15 (s, 3H); ^13^C-NMR (*d*_6_-DMSO, 100 MHz) δ 171.3, 147.8, 132.7, 128.6, 122.7, 116.5, 113.7, 20.0. 

*2-Amino-4-methylbenzamide* (**2r**): Known compound. 146.1 mg, 97% yield. White solid. m.p.: 148.9–149.5 °C. ^1^H-NMR (*d*_6_-DMSO, 400 MHz) δ 7.62 (bs, 1H), 7.43 (d, *J* = 8.1 Hz, 1H), 6.92 (bs, 1H), 6.53 (bs, 2H), 6.47 (s, 1H), 6.29 (d, *J* = 8.5 Hz, 1H), 2.16 (s, 3H); ^13^C-NMR (*d*_6_-DMSO, 100 MHz) δ 171.2, 150.3, 141.6, 128.8, 116.4, 115.6, 111.1, 21.0.

*Amino-6-chlorobenzamide* (**2s**): Known compound. 135.6 mg, 79% yield. White solid. m.p.: 131.6–132.3 °C. ^1^H-NMR (*d*_6_-DMSO, 400 MHz) δ 7.81 (bs, 1H), 7.58 (bs, 1H), 7.01 (t, *J* = 8.0 Hz, 1H), 6.64 (d, *J* = 8.1 Hz, 1H), 6.58 (d, *J* = 7.8 Hz, 1H), 5.21 (bs, 2H); ^13^C-NMR (*d*_6_-DMSO, 100 MHz) δ 167.5, 147.0, 130.0, 129.8, 121.7, 116.1, 113.6.

*2-Amino-4-chlorobenzamide* (**2t**): Known compound. 155.2 mg, 91% yield. White solid. m.p.: 179.7–180.6 °C. ^1^H-NMR (*d*_6_-DMSO, 400 MHz) δ 7.81 (bs, 1H), 7.57 (d, *J* = 8.5 Hz, 1H), 7.19 (bs, 1H), 6.86 (bs, 2H), 6.77 (d, *J* = 2.2 Hz, 1H), 6.52 (dd, *J* = 8.5 and 2.2 Hz, 1H); ^13^C-NMR (*d*_6_-DMSO, 100 MHz) δ 170.4, 151.5, 136.3, 130.6, 115.1, 114.0, 112.4.

*2-Amino-4-(trifluoromethyl)benzamide* (**2u**): Known compound. 175.8 mg, 86% yield. White solid. m.p.: 150.8–151.1 °C. ^1^H-NMR (*d*_6_-DMSO, 400 MHz) δ 7.96 (bs, 1H), 7.73 (d, *J* = 8.2 Hz, 1H), 7.36 (s, 1H), 7.07 (d, *J* = 1.1 Hz, 1H), 6.91 (bs, 2H), 6.78 (dd, *J* = 8.2 and 1.7 Hz, 1H); ^13^C-NMR (*d*_6_-DMSO, 100 MHz) δ 170.2, 150.2, 131.8 (q, *J* = 31.0 Hz), 129.9, 124.0 (d, *J* = 271.1 Hz), 116.7, 112.5 (d, *J* = 4.0 Hz), 109.9 (d, *J* = 3.6 Hz). 

*3-Aminobenzamide* (**2v**): Known compound. 125.5 mg, 92% yield. Yellow solid. m.p.: 112.1–112.7 °C. ^1^H-NMR (*d*_6_-DMSO, 400 MHz) δ 7.74 (bs, 1H), 7.15 (bs, 1H), 7.10–7.07 (m, 2H), 7.02–7.00 (m, 1H), 6.72–6.70 (m, 1H), 5.21 (bs 2H); ^13^C-NMR (*d*_6_-DMSO, 100 MHz) δ 168.7, 148.5, 135.2, 128.5, 116.5, 114.7, 113.1. 

*Isophthalamide* (**2w**): Known compound. 136.1 mg, 83% yield. Pale yellow solid. m.p.: > 300 °C. ^1^H-NMR (*d*_6_-DMSO, 400 MHz) δ 8.42 (bs, 1H), 8.13 (bs, 2H), 8.03 (dd, *J* = 7.7 and 1.7 Hz, 2H), 7.57 (t, *J* = 7.7 Hz, 1H), 7.50 (bs, 2H); ^13^C-NMR (*d*_6_-DMSO, 100 MHz) δ 167.5, 134.4, 130.1, 128.2, 126.8.

*Terephthalamide* (**2x**): Known compound. 143.0 mg, 87% yield. Pale yellow solid. m.p.: > 300 °C. ^1^H-NMR (*d*_6_-DMSO, 400 MHz) δ 8.11 (bs, 2H), 7.97 (s, 4H), 7.52 (bs, 2H); ^13^C-NMR (*d*_6_-DMSO, 100 MHz) δ 167.3, 136.5, 127.3.

*4-(Cyanomethyl)benzamide* (**2y**): Known compound. 123.6 mg, 77% yield. White solid. m.p.: > 300 °C. ^1^H-NMR (*d*_6_-DMSO, 400 MHz) δ 8.02 (bs, 1H), 7.94–7.92 (m, 1H), 7.92 (t, *J* = 1.8 Hz, 1H), 7.46 (d, *J* = 8.4 Hz, 2H), 7.43 (bs, 1H), 4.15 (s, 2H); ^13^C-NMR (*d*_6_-DMSO, 100 MHz) δ 167.3, 134.4, 133.6, 128.1, 127.9, 118.9, 22.2. 

*Cinnamamide* (**2z**): Known compound. 123.0 mg, 84% yield. White solid. m.p.: 148.2–148.8 °C. ^1^H-NMR (*d*_6_-DMSO, 400 MHz) δ 7.56 (d, *J* = 6.9 Hz, 3H), 7.42–7.36 (m, 4H), 7.15 (bs, 1H), 6.63 (d, *J* = 15.9 Hz 1H); ^13^C-NMR (*d*_6_-DMSO, 100 MHz) δ 166.7, 139.1, 134.9, 129.4, 128.9, 127.5, 122.3.

*Phenyl(o-tolyl)methanone* (**2aa**): Known compound. 144.4 mg, 63% yield. Yellow solid. m.p.: 160.9–162.5 °C. ^1^H-NMR (*d*_6_-DMSO, 400 MHz) δ 7.35 (bs, 1H), 6.98 (bs, 1H), 4.80 (t, *J* = 1.8 Hz, 2H), 4.36 (t, *J* = 1.8 Hz, 2H), 4.20 (s, 5H); ^13^C-NMR (*d*_6_-DMSO, 100 MHz) δ 171.5, 76.9, 70.4, 69.8, 69.0. 

*Furan-2-carboxamide* (**2a′**): Known compound. 79.0 mg, 71% yield. White solid. m.p.: 140.1–141.3 °C. ^1^H-NMR (*d*_6_-DMSO, 400 MHz) δ 7.83 (t, *J* = 0.7 Hz, 1H), 7.80 (bs, 1H), 7.41 (bs,1H), 7.14 (d, *J* = 3.4 Hz, 1H), 6.62 (q, *J* = 1.7 Hz, 1H); ^13^C-NMR (*d*_6_-DMSO, 100 MHz) δ 159.4, 148.0, 145.0, 113.6, 111.8. 

*Thiophene-2-carboxamide* (**2b′**): Known compound. 101.6 mg, 80% yield. White solid. m.p.: 178.2–179.3 °C. ^1^H-NMR (*d*_6_-DMSO, 400 MHz) δ 7.95 (bs, 1H), 7.74 (s, 2H), 7.37 (bs, 1H), 7.13 (t, *J* = 3.9 Hz, 1H); ^13^C-NMR (*d*_6_-DMSO, 100 MHz) δ 162.8, 140.3, 130.9, 128.6, 127.8.

*Thiazole-2-carboxamide* (**2c′**): Known compound. 78.1 mg, 61% yield. White solid. m.p.: 119.0–122.1 °C. ^1^H-NMR (*d*_6_-DMSO, 400 MHz) δ 8.21 (bs, 1H), 8.06 (d, *J* = 3.1 Hz, 1H), 8.03 (d, *J* = 3.1 Hz, 1H), 7.88 (bs, 1H); ^13^C-NMR (*d*_6_-DMSO, 100 MHz) δ 164.3, 160.9, 143.9, 125.9.

*Picolinamide* (**2d′**): Known compound. 83.3 mg, 69% yield. White solid. m.p.: 106.3–108.8 °C. ^1^H-NMR (CDCl_3_, 400 MHz) δ 8.56 (d, *J* = 4.7 Hz, 1H), 8.19 (d, *J* = 7.8 Hz, 1H), 7.90 (bs, 1H), 7.83 (td, *J* = 7.7 and 1.0 Hz, 1H), 7.44–7.41 (m, 1H), 6.41 (bs, 1H); ^13^C-NMR (CDCl_3_, 100 MHz) δ 167.2, 149.7, 148.4, 137.4, 126.5, 122.5.

*Nicotinamide* (**2e’**): Known compound. 91.2 mg, 75% yield. White solid. m.p.: 134.4–137.5 °C. ^1^H-NMR (*d*_6_-DMSO, 400 MHz) δ 9.03 (d, *J* = 1.4 Hz, 1H), 8.69 (dd, *J* = 4.7 and 1.4 Hz, 1H), 8.22–8.19 (m, 1H), 8.18 (bs, 1H), 7.63 (bs, 1H), 7.49 (dd, *J* = 7.8 and 4.8 Hz, 1H); ^13^C-NMR (*d*_6_-DMSO, 100 MHz) δ 166.5, 151.9, 148.7, 135.2, 129.7, 123.4.

*Isonicotinamide* (**2f′**): Known compound. 104.8 mg, 86% yield. White solid. m.p.: 151.1–153.9 °C. ^1^H-NMR (*d*_6_-DMSO, 400 MHz) δ 8.64 (dd, *J* = 4.3 and 1.5 Hz, 2H), 7.78 (dd, *J* = 4.3 and 1.6 Hz, 2H); ^13^C-NMR (*d*_6_-DMSO, 100 MHz) δ 167.3, 149.6, 144.6, 123.1.

*1H-Indole-4-carboxamide* (**2g′**): Known compound. 130.9 mg, 82% yield. Pale yellow solid. m.p.: 143.2–145.7 °C. ^1^H-NMR (*d*_6_-DMSO, 400 MHz) δ 11.3 (bs, 1H), 7.75 (bs, 1H), 7.58 (d, *J* = 8.0 Hz, 1H), 7.51 (d, *J* = 7.3 Hz, 1H), 7.46 (t, *J* = 2.8 Hz, 1H), 7.25 (bs, 1H), 7.16 (t, *J* = 7.7 Hz, 1H), 6.96 (t, *J* = 2.0 Hz, 1H); ^13^C-NMR (*d*_6_-DMSO, 100 MHz) δ 169.8, 136.6, 126.4, 126.2, 126.1, 120.0, 118.9, 114.2, 102.0.

*Phenylacetamide* (**2a″**): Known compound. 109.4 mg, 81% yield. White solid. m.p.: 152.6–155.1 °C. ^1^H-NMR (CDCl_3_, 400 MHz) δ 7.38–7.34 (m, 2H), 7.32–7.27 (m, 3H), 5.82 (bs, 1H), 5.41 (bs, 1H), 3.58 (s, 2H); ^13^C-NMR (CDCl_3_, 100 MHz) δ 173.7, 135.0, 129.5, 129.2, 127.6, 43.5.

*1,2,3,4-Tetrahydronaphthalene-1-carboxamide* (**2b″**): Known compound. 130.3 mg, 74% yield. White solid. m.p.: > 300 °C. ^1^H-NMR (*d*_6_-DMSO, 400 MHz) δ 7.49 (bs, 1H), 7.15–7.09 (m, 4H), 6.99 (bs, 1H), 3.64 (t, *J* = 6.8 Hz, 1H), 2.75–2.72 (m, 2H), 1.97–1.91 (m, 3H), 1.69–1.60 (m, 1H); ^13^C-NMR (*d*_6_-DMSO, 100 MHz) δ 176.2, 137.0, 135.3, 128.9, 128.4, 126.0, 125.4, 45.0, 28.8, 26.9, 20.6.

*Benzothioamide* (**3a**): Known compound. 99.8 mg, 73% yield. Yellow solid. m.p.: 114.5–115.7 °C. ^1^H-NMR (CDCl_3_, 400 MHz) δ 7.95 (bs, 1H), 7.87–7.86 (m, 1H), 7.85–7.84 (m, 1H), 7.52–7.48 (m, 1H), 7.42–7.37 (m, 2H), 7.30 (bs, 1H); ^13^C-NMR (CDCl_3_, 100 MHz) δ 202.9, 139.2, 132.1, 128.6, 127.0. 

*2-Benzoylquinazolin-4(3H)-one* (**3c**): Known compound. 188.0 mg, 75% yield. White solid. m.p.: 182.5–183.9 °C. ^1^H-NMR (CDCl_3_, 400 MHz) δ 10.5 (bs, 1H), 8.52–8.49 (m, 2H), 8.39 (dd, *J* = 7.9 and 1.4 Hz, 1H), 7.93–7.91 (m, 1H), 7.86–7.82 (m, 1H), 7.69–7.61 (m, 2H), 7.56–7.52 (m, 2H); ^13^C-NMR (CDCl_3_, 100 MHz) δ 185.7, 161.1, 147.6, 146.1, 134.9, 134.4, 134.1, 131.9, 129.5 x 2, 128.5, 127.0, 123.4.

## 4. Conclusions

In conclusion, we have developed an environmentally friendly and practical methodology for the hydrolysis of nitriles to amides in WEPPA with transition metal catalysts, external bases, and organic solvent-free conditions. A variety of substrates including aryl, heteroaryl, vinyl, and alkyl nitriles with high functional group compatibility were tolerated to deliver the desired products with moderate to excellent yields. This hydrolysis reaction could easily be scaled up to 10 or even 100 mmol with good yields and WEPPA could be reused at least four times. This work opens the way for the reclamation of agricultural waste. Further applications of AWEs in other organic reactions are ongoing in our laboratory and will be reported on in due course.

## Figures and Tables

**Table 1:** ICP-AES analysis of the pomelo peel ash.

Entry	Element	wt %
1	K	21.36
2	Na	0.12
3	Ca	7.41
4	Mg	1.92
5	Cu	0.0054
6	Fe	0.018
7	Mn	0.012

**Table 2:** XPS analysis.

Element	Start BE	Peak BE	End BE	Height CPS	FWHM eV	Area (P) CPS.eV	Area (N)	Atomic %
O	537.48	531.34	524.28	461018.18	2.4	1200937.11	6963.79	33.76
K	298.68	293.22	290.76	270622.92	2.4	704963.77	2127.97	10.32
C	290.69	284.78	277.28	342691.77	2.05	760946.07	10670.16	51.73
Ca	360.08	347.26	343.68	60779.2	2.4	158327.81	387.4	1.88
P	138.08	133.08	124.88	7161.84	2.4	18656.35	176.39	0.86
S	174.68	168.95	159.88	6485.17	2.4	16893.65	117.31	0.57
Cl	210.08	198.79	188.08	5350.36	2.4	13937.51	67.6	0.33
Si	105.68	102.24	93.28	3240.34	2.4	8440.99	117.89	0.57

**Table 3:** Optimization of reaction conditions for the hydration of benzonitrile **1a** in AWEs ^a^.

Entry	AWEs	T (°C)	t (h)	Conv. (%) ^b^
1	WEWSA	100	12	7
2	WEHMPA	100	12	19
3	WEAA	100	12	Trace
4	WEBSA	100	12	13
5	WEPPA	100	12	41
6	WEPA	100	12	7
7 ^c^	WEPPA(C)	100	12	N.R.
8 ^c^	WEWSA(C)	100	12	N.R.
9	WEPPA	100	24	43
10	WEPPA	100	36	50
11	WEPPA	120	24	83
12	WEPPA	130	24	93
13	WEPPA	140	24	97
14	WEPPA	100	0.5	49
15	WEPPA	120	0.5	56
16	WEPPA	140	0.5	88
17 ^d^	WEPPA	150	0.5	>99 (94)
18	WEPPA	150	0.25	88
19 ^e^	------	150	0.5	N.R.

^a^ Reaction conditions: **1a** (1.0 mmol), AWEs (2.0 mL), for entries 1–13 reacted in the oil bath, for entries 14–19 reacted in the closed vessel synthesis reactor. ^b^ Determined by GC analysis. ^c^ The dried walnut shell and pomelo peel were obtained by calcination at 300 °C for 3 h with a rising rate of 0.5 °C min^−1^. ^d^ Isolated yield of **2a** in parentheses. ^e^ Distilled water. WEWSA: water extract of walnut shell ash, WEHMPA: water extract of Hami melon peel ash, WEAA: water extract of alfalfa ash, WEBSA: water extract of badam shell ash, WEPPA: water extract of pomelo peel ash, WEPA: water extract of pomegranate ash. N.R. = no reaction.

**Table 4:**
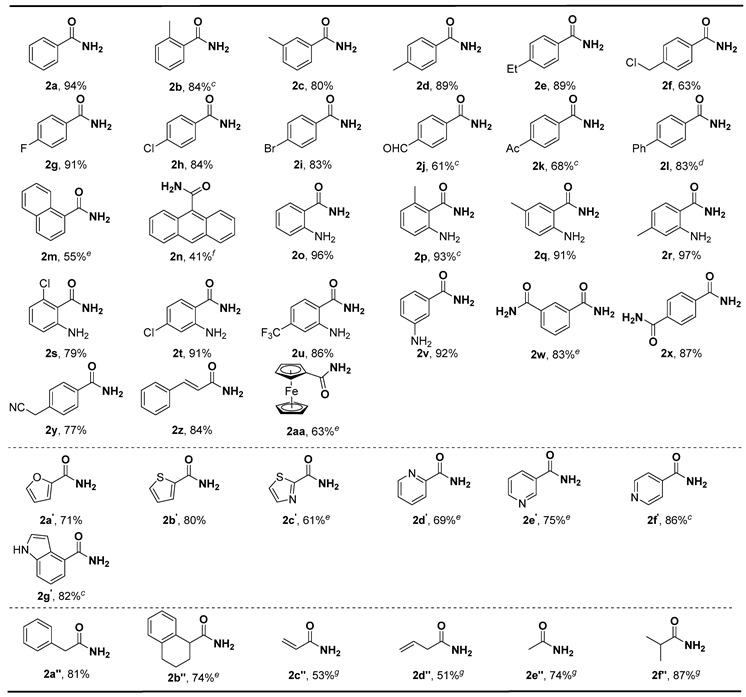
The substrate scope for the hydration of nitriles **1** in WEPPA ^a,b^.

^a^ Reaction conditions: **1** (1.0 mmol), WEPPA (2.0 mL), 150 °C, 0.5 h, closed vessel synthesis reactor. ^b^ Isolated yields. ^c^ 0.75 h. ^d^ 2 h. ^e^ 1 h. ^f^ 4 h. ^g^ GC yields.

**Table 5:**
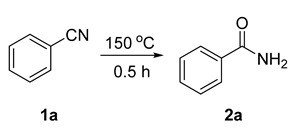
The conversions of **1a** in the water solutions of different inorganic carbonates or oxides ^a^.

Entry	Compound	Loading (g/10 mL)	GC yields (%) ^b^
1	K_2_CO_3_	0.2136	17
2	Na_2_CO_3_	1.2 × 10^−3^	N.R
3	CaCO_3_	0.0741	N.R
4	MgCO_3_	0.0192	Trace
5	CuCO_3_	0.054 × 10^−3^	N.R
6	MnCO_3_	0.012	N.R
7	CaO	0.0741	40
8	MgO	0.0192	N.R
9	CuO	0.054 × 10^−3^	N.R
10	Fe_2_O_3_	0.18 × 10^−3^	N.R
11	MnO_2_	0.012	N.R
12 ^c^	mixture		26

^a^ Reaction conditions: **1a** (1.0 mmol), water solutions of inorganic carbonates or oxides (2.0 mL), 150 °C, 0.5 h. ^b^ Determined by GC analysis. ^c^ According to the ICP analysis, water solution of all these inorganic carbonates and oxides entries 1–11.

## Supplementary Materials

Supplementary materials are available online.
