# Supplementary material for: Trash to Treasure: Eco-Friendly and Practical Synthesis of Amides by Nitriles Hydrolysis in WEPPA
Source: Molecules. 2019 Oct 24;24(21):3838. doi: 10.3390/molecules24213838 (PMC6864965; doi:10.3390/molecules24213838)

Supporting Information

Trash to Treasure: Eco-Friendly and Practical Synthesis of Amides by Nitriles Hydrolysis in WEPPA

Yajun Sun, Weiwei Jin,* and Chenjiang Liu*

The Key Laboratory of Oil and Gas Fine Chemicals, Ministry of Education & Xinjiang Uygur Autonomous Region, Urumqi Key Laboratory of Green Catalysis and Synthesis Technology, School of Chemistry and Chemical Engineering, Xinjiang University, Urumqi 830046, P. R. China.
E-mail: wwjin0722@163.com; pxylcj@126.com

Table of Contents

1. General and materials S2
2. General procedure for the preparation of AWEs S3
3. XPS spectrum of the pomelo peel ash S3
4. General procedure for the hydrolysis of nitriles in WEPPA S4
5. Gram-scale experiments S4
6. Recycling experiments S5
7. Comparative experiments S5
8. Analytical data S7
9. References S17
10. Copies of NMR spectra S19

1. General and materials

General. ^{1}H, ^{13}C and ^{19}F NMR spectra were recorded on a Varian Inova-400 (400 MHz, 100 MHz and 376 MHz, respectively) spectrometer. ^{1}H and ^{13}C NMR chemical shifts were determined relative to internal standard TMS at δ 0.0 or $CDCl_3$ (δ(^{1}H), 7.26 ppm; δ(^{13}C), 77.16 ppm) or d_6-DMSO (δ(^{1}H), 2.54 ppm; δ(^{13}C), 39.50 ppm) and ^{19}F NMR chemical shifts were determined relative to $CFCl_3$ as internal standard. Chemical shifts (δ) are reported in ppm, and coupling constants (*J*) are in Hertz (Hz). The following abbreviations are used to explain the multiplicities: s = singlet, d = doublet, t = triplet, q = quartet, m = multiplet, bs = broad singlet. pH values were detected by PHS-3C acidometer. Inductively coupled plasma atomic emission spectroscopy (ICP-AES) analysis was carried out on a Varian VISTA-PRO spectrometer. X-Ray photoelectron spectroscopy (XPS) was detected on a Thermo Scientific K-Alpha+X spectrometer. Energy dispersive X-ray (EDX) was recorded on the SU8010 cold field emission ultra-high resolution scanning electron microscope. The melting point was recorded on BÜCHI (M-560) and uncorrected. Analytical thin layer chromatography (TLC) was performed on 0.25 mm silica gel 60 F254 plates and viewed by UV light (254 nm). Column chromatographic purification was performed using 200-300 mesh silica gel.

Materials. All the chemical reagents were purchased from commercial sources and used as received unless otherwise indicated.

2. General procedure for the preparation of AWEs (taking WEPPA as an example)

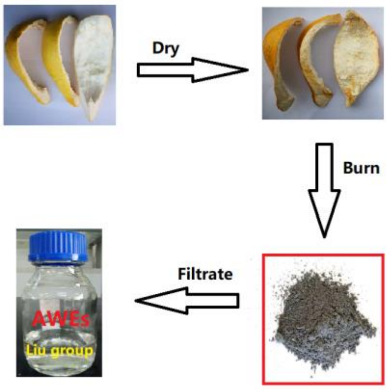

The pomelo peel was obtained and dried naturally. The dried pomelo peel was burned to get its ash. Then, one gram pomelo peel ash was suspended into 10.0 mL of distilled water at room temperature for 30 min with constant stiring. The suspension was then filtered to obtain a pale yellow extract which named as WEPPA.

3. XPS spectrum of the pomelo peel ash

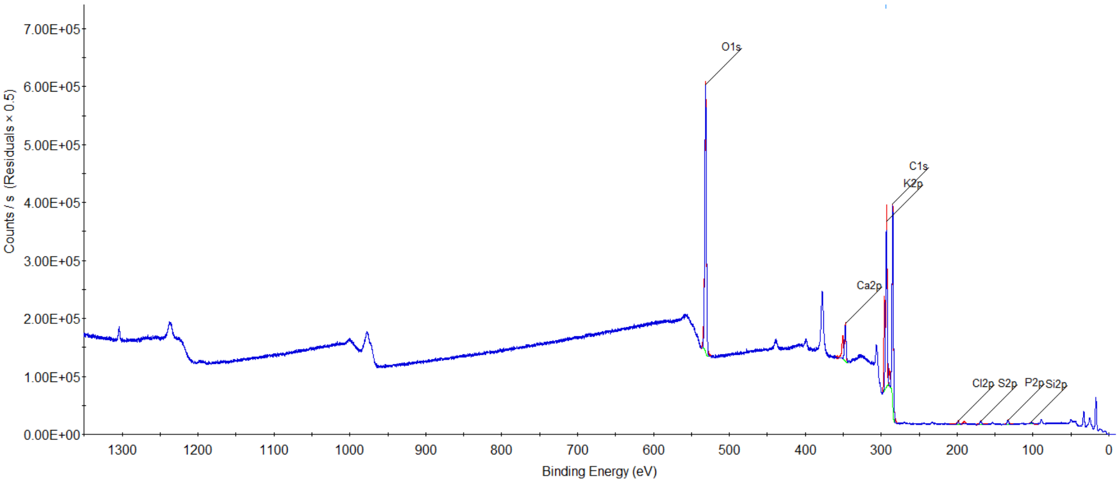

Figure S1 XPS spectrum of the pomelo peel ash

Table S1 XPS analysis

Element	Start BE	Peak BE	End BE	Height CPS	FWHM eV	Area (P) CPS.eV	Area (N)	Atomic %
O	537.48	531.34	524.28	461018.18	2.4	1200937.11	6963.79	33.76
K	298.68	293.22	290.76	270622.92	2.4	704963.77	2127.97	10.32
C	290.69	284.78	277.28	342691.77	2.05	760946.07	10670.16	51.73
Ca	360.08	347.26	343.68	60779.2	2.4	158327.81	387.4	1.88

P	138.08	133.08	124.88	7161.84	2.4	18656.35	176.39	0.86
S	174.68	168.95	159.88	6485.17	2.4	16893.65	117.31	0.57
Cl	210.08	198.79	188.08	5350.36	2.4	13937.51	67.6	0.33
Si	105.68	102.24	93.28	3240.34	2.4	8440.99	117.89	0.57

4. General procedure for the hydrolysis of nitriles in WEPPA (taking 1a as an example)

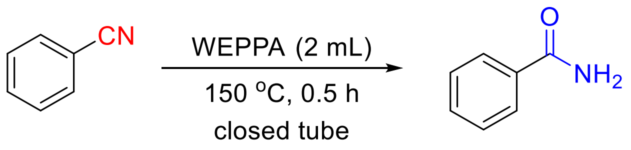

1a **2a**

Under atmosphere, benzonitrile **1a** (103 mg, 1.0 mmol) and WEPPA (2.0 mL) were added into a 10 mL closed tube with a stir bar. Then the reaction was stirred in a closed vessel synthesis reactor at 150 °C for 0.5 h. After cooling to ambient temperature, the resulting precipitate was collected by filtration, washed with ice water and further dried in the vacuum drying oven. The filtrate was evaporated under reduced pressure. The resultant residue was purified by silica gel column chromatography (eluent: petroleum ether (35-60 °C)/EtOAc = 2:1 to 0:1, v/v). Finally, cmbining these two parts to afford the desired benzamide **2a** in 85% yield.

5. Gram-scale experiments (taking 1o at 100 mmol as an example)

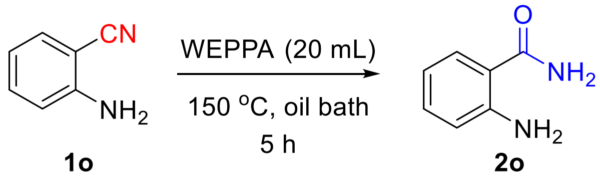

1o (11.8 g, 100 mmol) **2o** (11.6 g, 85% yield)

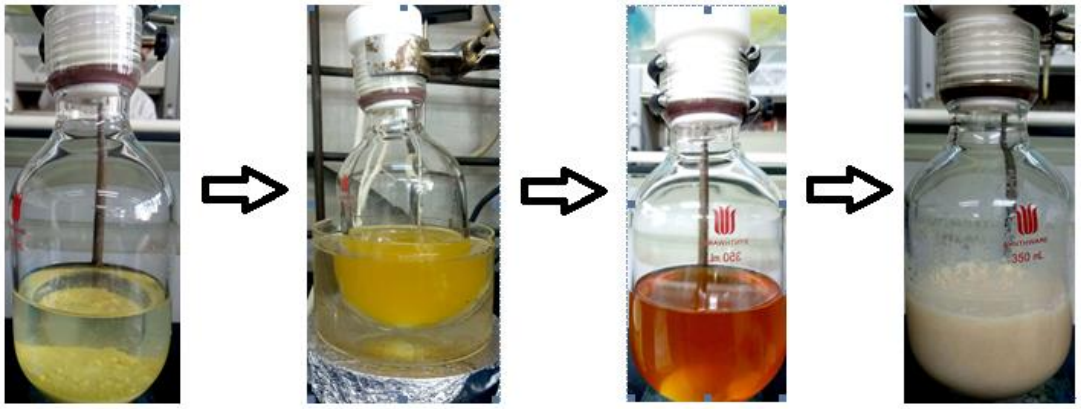

before the reaction **during the reaction** **after the reaction** **after the reaction for a while**

Under atmosphere, 2-aminobenzonitrile **1o** (11.8 g, 100.0 mmol) and WEPPA (150.0 mL) were added into a 300 mL closed tube with a stir bar. Then the reaction was stirred in an oil bath at 150 °C for 5 h. After cooling to ambient temperature, large amount of white solid precipitated out and was collected by filtration, washed with ice water and further dried in the vacuum drying oven. The filtrate was evaporated under reduced pressure to get the residual product. Finally, cmbining these two parts to afford the desired 2-aminobenzamide **2o** (11.6 g) in 85% yield.

6. Recycling experiments

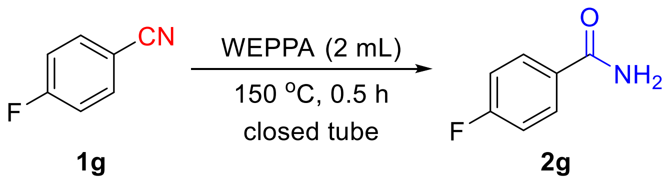

Under atmosphere, 4-fluorobenzamide **1g** (121 mg, 1.0 mmol) and WEPPA (2.0 mL) were added into a 10 mL closed tube with a stir bar. Then the reaction was stirred in a closed vessel synthesis reactor at 150 °C for 0.5 h. After cooling to ambient temperature, the resulting precipitate was collected by filtration, washed with ice water and further dried in the vacuum drying oven. The WEPPA filtrate could be reused at least four times in good yields (89%, 88%, 84% and 75%).

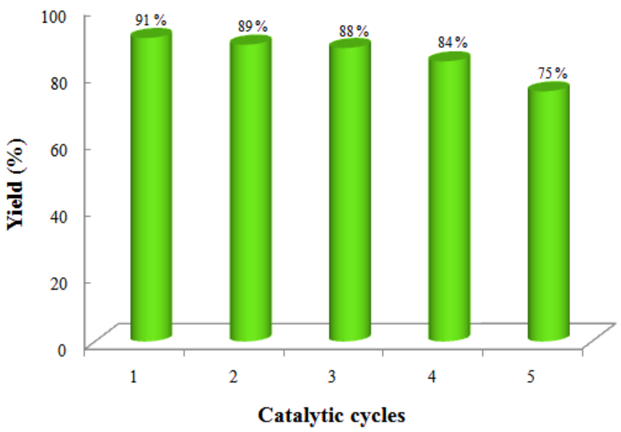

7. Comparative experiments

Table S2 The conversions of 1a in the water solutions of different inorganic carbonates or oxides*[a]*

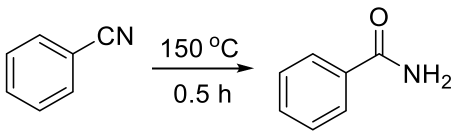

1a **2a**

Entry	Compound	Loading (g/10 mL)	GC yields (%)[b]
1	K_2CO_3	0.2136	17
2	Na_2CO_3	$1.2*10^{-3}$	N.R
3	$CaCO_3$	0.0741	N.R
4	$MgCO_3$	0.0192	Trace
5	$CuCO_3$	$0.054*10^{-3}$	N.R
6	$MnCO_3$	0.012	N.R
7	CaO	0.0741	40
8	MgO	0.0192	N.R
9	CuO	$0.054*10^{-3}$	N.R
10	Fe_2O_3	$0.18*10^{-3}$	N.R
11	MnO_2	0.012	N.R
12[c]	mixture		26

[a] Reaction conditions: **1a** (1.0 mmol), water solutions of inorganic carbonates or oxides (2.0 mL), 150 °C, 0.5 h. [b] Determined by GC analysis. [c] According to the ICP analysis, water solution of all these inorganic carbonates and oxides entries 1-11.

8. Analytical data

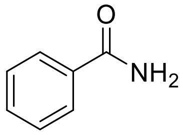

Benzamide (2a)[1]: Known compound. 114.2 mg, 94% yield. White solid. m.p.: 127.3-129.1 °C. **^{1}H NMR** ($CDCl_3$, 400 MHz) δ 7.83-7.80 (m, 2H), 7.54-7.50 (m, 1H), 7.46-7.42 (m, 2H), 6.26 (bs, 2H); **^{13}C NMR** ($CDCl_3$, 100 MHz) δ 169.7, 133.5, 132.1, 128.8, 127.5.

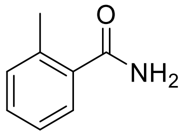

2-Methylbenzamide (2b)[1]: Known compound. 114.1 mg, 84% yield. White solid. m.p.: 140.1-142.9 °C. **^{1}H NMR** ($CDCl_3$, 400 MHz) δ 7.43 (d, *J* = 7.6 Hz, 1H), 7.32 (td, *J* = 7.6 and 1.3 Hz, 1H), 7.21 (q, *J* = 7.2 Hz, 2H), 6.28 (bs, 1H), 5.86 (bs, 1H), 2.49 (s, 3H); **^{13}C NMR** ($CDCl_3$, 100 MHz) δ 172.4, 136.4, 135.4, 131.3, 130.4, 127.1, 125.8, 20.1.

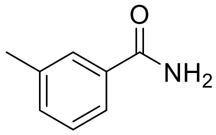

3-Methylbenzamide (2c)[1]: Known compound. 108.2 mg, 80% yield. White solid. m.p.: 90.1-91.1 °C. **^{1}H NMR** ($CDCl_3$, 400 MHz) δ 7.65 (s, 1H), 7.60-7.58 (m, 1H), 7.33-7.29 (m, 2H), 6.30 (bs, 2H), 2.39 (s, 3H); **^{13}C NMR** ($CDCl_3$, 100 MHz) δ 170.0, 138.6, 133.5, 132.8, 128.6, 128.2, 124.4, 21.4.

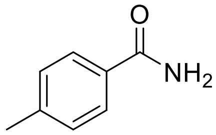

4-Methylbenzamide (2d)[1]: Known compound. 120.2 mg, 89% yield. White solid. m.p.: 148.1-148.8 °C. **^{1}H NMR** ($CDCl_3$, 400 MHz) δ 7.71 (d, *J* = 8.2 Hz, 2H), 7.25 (d, *J* = 9.0 Hz, 2H), 5.93 (bs, 2H), 2.42 (s, 3H); **^{13}C NMR** ($CDCl_3$, 100 MHz) δ 169.5, 142.7, 130.6, 129.4, 127.5, 21.6.

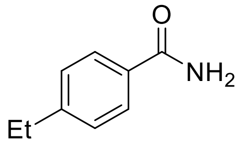

4-Ethylbenzamide (2e)[2]: Known compound. 132.6 mg, 89% yield. White solid. m.p.: 160.2 -162.5 °C. **^{1}H NMR** ($CDCl_3$, 400 MHz) δ 7.74 (d, *J* = 8.3 Hz, 2H), 7.27 (d, *J* = 8.3 Hz, 2H), 6.06 (bs, 2H), 2.70 (q, *J* = 7.6 Hz, 2H), 1.25 (t, *J* = 7.6 Hz, 3H); **^{13}C NMR** ($CDCl_3$, 100 MHz) δ 169.7, 148.8, 130.9, 128.2, 127.6, 28.9, 15.4.

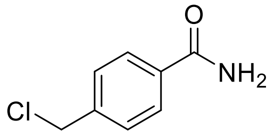

4-(Chloromethyl)benzamide (2f)[3]: Known compound. 107.2 mg, 63% yield. White solid. m.p.: 133.3 -135.1 °C. **^{1}H NMR** (d_6-DMSO, 400 MHz) δ 7.92 (bs, 1H), 7.84 (d, *J* = 8.2 Hz, 2H), 7.38 (d, *J* = 8.0 Hz, 2H), 7.30 (bs, 1H), 4.56 (s, 2H); **^{13}C NMR** (d_6-DMSO, 100 MHz) δ 167.8, 145.9, 132.6, 127.3, 125.9, 62.5.

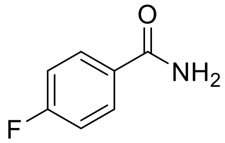

4-Fluorobenzamide (2g)[1]: Known compound. 127.0 mg, 91% yield. White solid. m.p.: 155.3-155.5 °C. **^{1}H NMR** (d_6-DMSO, 400 MHz) δ 8.03 (bs, 1H), 8.00-7.96 (m, 2H), 7.43 (bs, 1H), 7.34-7.28 (m, 2H); **^{13}C NMR** (d_6-DMSO, 100 MHz) δ 166.8, 163.9 (d, *J* = 245.8 Hz), 130.7 (d, *J* = 11.5 Hz), 130.1 (d, *J* = 9.0 Hz), 115.1 (d, *J* = 21.6 Hz); **^{19}F NMR** (d_6-DMSO, 376 MHz) δ -109.6.

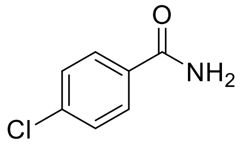

4-Chlorobenzamide (2h)[1]: Known compound. 130.1 mg, 84% yield. White solid. m.p.: 177.4-178.8 °C. **^{1}H NMR** ($CDCl_3$, 400 MHz) δ 7.77-7.74 (m, 2H), 7.45-7.42 (m, 2H), 5.85 (bs, 2H); **^{13}C NMR** ($CDCl_3$, 100 MHz) δ 168.3, 138.5, 131.8, 129.1, 128.9.

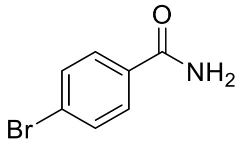

4-Bromobenzamide (2i)[1]: Known compound. 165.0 mg, 83% yield. White solid. m.p.: 188.9-191.6 °C. **^{1}H NMR** (d_6-DMSO, 400 MHz) δ 8.08 (bs, 1H), 7.87-7.84 (m, 2H), 7.72-7.68 (m, 2H), 7.49 (bs, 1H); **^{13}C NMR** (d_6-DMSO, 100 MHz) δ 166.9, 133.4, 131.2, 129.6, 125.0.

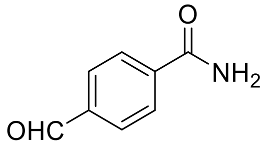

4-Formylbenzamide (2j)[4]: Known compound. 91.0 mg, 61% yield. White solid. m.p.: 178.9-182.1 °C. **^{1}H NMR** (d_6-DMSO, 400 MHz) δ 10.09 (bs, 1H), 8.19 (bs, 1H), 8.07 (d, J = 7.9 Hz, 2H), 7.99 (d, J = 8.0 Hz, 2H), 7.62 (bs, 1H); **^{13}C NMR** (d_6-DMSO, 100 MHz) δ 192.9, 167.0, 139.3, 137.8, 129.3, 128.1.

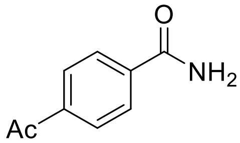

4-Acetylbenzamide (2k)[1]: Known compound. 110.8 mg, 68% yield. Yellow solid. m.p.: 192.5-194.1 °C. **^{1}H NMR** (d_6-DMSO, 400 MHz) δ 8.15 (bs, 1H), 8.04-7.98 (m, 4H), 7.57 (bs, 1H), 2.62 (s, 3H); **^{13}C NMR** (d_6-DMSO, 100 MHz) δ 197.7, 167.1, 138.6, 138.1, 128.1, 127.7, 26.9.

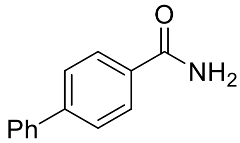

[1,1'-Biphenyl]-4-carboxamide (2l)[5]: Known compound. 164.1 mg, 83% yield. White solid. m.p.: 232.1-234.5 °C. **^{1}H NMR** (d_6-DMSO, 400 MHz) δ 8.08 (bs, 1H), 8.02 (d, J = 8.4 Hz, 2H), 7.80 (bs, 1H), 7.78-7.75 (m, 3H), 7.53 (t, J = 7.3 Hz, 2H), 7.44 (t, J = 7.2 Hz, 2H); **^{13}C NMR** (d_6-DMSO, 100 MHz) δ 167.5, 142.7, 139.2, 133.1, 129.0, 128.1, 128.0, 126.8, 126.4.

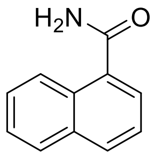

1-Naphthamide (2m)[1]: Known compound. 94.6 mg, 55% yield. White solid. m.p.: 204.8-206.2 °C. **^{1}H NMR** (d_6-DMSO, 400 MHz) δ 8.36 (d, J = 7.3 Hz, 1H), 8.05-8.00

(m, 3H), 7.70-7.55 (m, 5H); ^{13}**C NMR** (d_6-DMSO, 100 MHz) δ 170.5, 134.6, 133.2, 129.7 x 2, 128.1, 126.6, 126.1, 125.6, 125.1, 124.9.

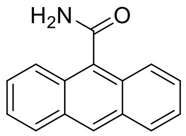

Anthracene-9-carboxamide (2n)[6]: Known compound. 90.7 mg, 41% yield. Yellow solid. m.p.: 186.2-188.6 °C. ^{1}**H NMR** (d_6-DMSO, 400 MHz) δ 8.68 (bs, 1H), 8.30 (bs, 1H), 8.16 (d, J = 7.9 Hz, 2H), 8.08 (d, J = 8.8 Hz, 3H), 7.64-7.57 (m, 4H); ^{13}**C NMR** (d_6-DMSO, 100 MHz) δ 170.2, 133.7, 130.7, 128.3, 126.8 x 2, 126.2, 125.5, 125.4.

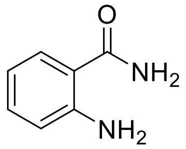

2-Aminobenzamide (2o)[1]: Known compound. 130.5 mg, 96% yield. Yellow solid. m.p.: 110.1-111.5 °C. ^{1}**H NMR** ($CDCl_3$, 400 MHz) δ 7.36 (dd, J = 7.9 and 1.3 Hz, 1H), 7.25-7.20 (m, 1H), 6.68 (d, J = 8.2 Hz, 1H), 6.66-6.62 (m, 1H), 5.90 (bs, 2H), 5.67 (bs, 2H); ^{13}**C NMR** ($CDCl_3$, 100 MHz) δ 171.8, 149.6, 133.1, 128.1, 117.6, 116.5, 114.1.

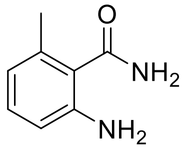

2-Amino-6-methylbenzamide (2p)[7]: Known compound. 139.9 mg, 93% yield. White solid. m.p.: 143.7-144.8 °C. ^{1}**H NMR** (d_6-DMSO, 400 MHz) δ 7.63 (bs, 1H), 7.42 (bs, 1H), 6.92 (t, J = 7.7 Hz, 1H), 6.51 (d, J = 7.9 Hz, 1H), 6.39 (d, J = 7.2 Hz, 1H), 4.90 (bs, 2H), 2.21 (s, 3H); ^{13}**C NMR** (d_6-DMSO, 100 MHz) δ 170.5, 145.4, 134.2, 128.7, 123.0, 117.9, 112.7, 19.9.

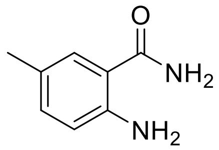

2-Amino-5-methylbenzamide (2q)[7]: Known compound. 135.9 mg, 90% yield. Yellow solid. m.p.: 172.6-174.3 °C. ^{1}**H NMR** (d_6-DMSO, 400 MHz) δ 7.65 (bs, 1H), 7.34 (bs, 1H), 6.95 (dd, J = 8.2 and 1.5 Hz, 2H), 6.59 (d, J = 8.2 Hz, 1H), 6.31 (bs,

2H), 2.15 (s, 3H); **^{13}C NMR** (*d_6*-DMSO, 100 MHz) δ 171.3, 147.8, 132.7, 128.6, 122.7, 116.5, 113.7, 20.0.

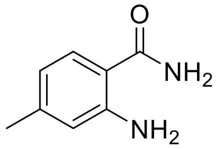

2-Amino-4-methylbenzamide (2r)[7]: Known compound. 146.1 mg, 97% yield. White solid. m.p.: 148.9-149.5 °C. **^{1}H NMR** (*d_6*-DMSO, 400 MHz) δ 7.62 (bs, 1H), 7.43 (d, *J* = 8.1 Hz, 1H), 6.92 (bs, 1H), 6.53 (bs, 2H), 6.47 (s, 1H), 6.29 (d, *J* = 8.5 Hz, 1H), 2.16 (s, 3H); **^{13}C NMR** (*d_6*-DMSO, 100 MHz) δ 171.2, 150.3, 141.6, 128.8, 116.4, 115.6, 111.1, 21.0.

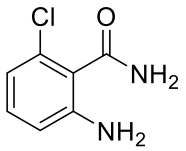

2-**Amino-6-chlorobenzamide (2s)**[8]: Known compound. 135.6 mg, 79% yield. White solid. m.p.: 131.6-132.3 °C. **^{1}H NMR** (*d_6*-DMSO, 400 MHz) δ 7.81 (bs, 1H), 7.58 (bs, 1H), 7.01 (t, *J* = 8.0 Hz, 1H), 6.64 (d, *J* = 8.1 Hz, 1H), 6.58 (d, *J* = 7.8 Hz, 1H), 5.21 (bs, 2H); **^{13}C NMR** (*d_6*-DMSO, 100 MHz) δ 167.5, 147.0, 130.0, 129.8, 121.7, 116.1, 113.6.

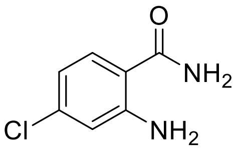

2-Amino-4-chlorobenzamide (2t)[9]: Known compound. 155.2 mg, 91% yield. White solid. m.p.: 179.7-180.6 °C. **^{1}H NMR** (*d_6*-DMSO, 400 MHz) δ 7.81 (bs, 1H), 7.57 (d, *J* = 8.5 Hz, 1H), 7.19 (bs, 1H), 6.86 (bs, 2H), 6.77 (d, *J* = 2.2 Hz, 1H), 6.52 (dd, *J* = 8.5 and 2.2 Hz, 1H); **^{13}C NMR** (*d_6*-DMSO, 100 MHz) δ 170.4, 151.5, 136.3, 130.6, 115.1, 114.0, 112.4.

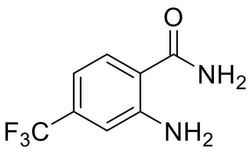

2-Amino-4-(trifluoromethyl)benzamide (2u)[10]: Known compound. 175.8 mg, 86% yield. White solid. m.p.: 150.8-151.1 °C. **^{1}H NMR** (*d_6*-DMSO, 400 MHz) δ 7.96 (bs,

1H), 7.73 (d, *J* = 8.2 Hz, 1H), 7.36 (s, 1H), 7.07 (d, *J* = 1.1 Hz, 1H), 6.91 (bs, 2H), 6.78 (dd, *J* = 8.2 and 1.7 Hz, 1H); **^{13}C NMR** (*d_6*-DMSO, 100 MHz) δ 170.2, 150.2, 131.8 (q, *J* = 31.0 Hz), 129.9, 124.0 (d, *J* = 271.1 Hz), 116.7, 112.5 (d, *J* = 4.0 Hz), 109.9 (d, *J* = 3.6 Hz).

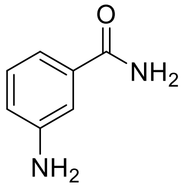

3-Aminobenzamide (2v)[10]: Known compound. 125.5 mg, 92% yield. Yellow solid. m.p.: 112.1-112.7 ℃. **^{1}H NMR** (*d_6*-DMSO, 400 MHz) δ 7.74 (bs, 1H), 7.15 (bs, 1H), 7.10-7.07 (m, 2H), 7.02-7.00 (m, 1H), 6.72-6.70 (m, 1H), 5.21 (bs 2H); **^{13}C NMR** (*d_6*-DMSO, 100 MHz) δ 168.7, 148.5, 135.2, 128.5, 116.5, 114.7, 113.1.

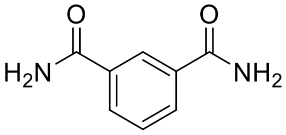

Isophthalamide (2w)[11]: Known compound. 136.1 mg, 83% yield. Pale yellow solid. m.p.: > 300 ℃. **^{1}H NMR** (*d_6*-DMSO, 400 MHz) δ 8.42 (bs, 1H), 8.13 (bs, 2H), 8.03 (dd, *J* = 7.7 and 1.7 Hz, 2H), 7.57 (t, *J* = 7.7 Hz, 1H), 7.50 (bs, 2H); **^{13}C NMR** (*d_6*-DMSO, 100 MHz) δ 167.5, 134.4, 130.1, 128.2, 126.8.

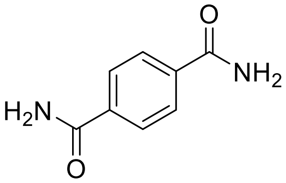

Terephthalamide (2x)[6]: Known compound. 143.0 mg, 87% yield. Pale yellow solid. m.p.: > 300 ℃. **^{1}H NMR** (*d_6*-DMSO, 400 MHz) δ 8.11 (bs, 2H), 7.97 (s, 4H), 7.52 (bs, 2H); **^{13}C NMR** (*d_6*-DMSO, 100 MHz) δ 167.3, 136.5, 127.3.

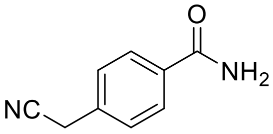

4-(Cyanomethyl)benzamide (2y)[12]: Known compound. 123.6 mg, 77% yield. White solid. m.p.: > 300 ^{o}C. **^{1}H NMR** (*d_6*-DMSO, 400 MHz) δ 8.02 (bs, 1H), 7.94-7.92 (m, 1H), 7.92 (t, *J* = 1.8 Hz, 1H), 7.46 (d, *J* = 8.4 Hz, 2H), 7.43 (bs, 1H), 4.15 (s, 2H); **^{13}C**

NMR (d_6-DMSO, 100 MHz) δ 167.3, 134.4, 133.6, 128.1, 127.9, 118.9, 22.2.

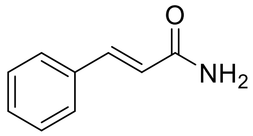

Cinnamamide (2z)[1] Known compound. 123.0 mg, 84% yield. White solid. m.p.: 148.2-148.8 °C. **^{1}H NMR** (d_6-DMSO, 400 MHz) δ 7.56 (d, J = 6.9 Hz, 3H), 7.42-7.36 (m, 4H), 7.15 (bs, 1H), 6.63 (d, J = 15.9 Hz 1H); **^{13}C NMR** (d_6-DMSO, 100 MHz) δ 166.7, 139.1, 134.9, 129.4, 128.9, 127.5, 122.3.

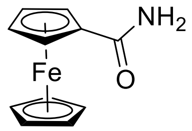

Phenyl(*o*-tolyl)methanone (2aa)[13]: Known compound. 144.4 mg, 63% yield. Yellow solid. m.p.: 160.9-162.5 °C. **^{1}H NMR** (d_6-DMSO, 400 MHz) δ 7.35 (bs, 1H), 6.98 (bs, 1H), 4.80 (t, J = 1.8 Hz, 2H), 4.36 (t, J = 1.8 Hz, 2H), 4.20 (s, 5H); **^{13}C NMR** (d_6-DMSO, 100 MHz) δ 171.5, 76.9, 70.4, 69.8, 69.0.

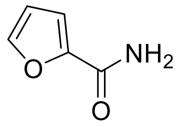

Furan-2-carboxamide (2a')[14]: Known compound. 79.0 mg, 71% yield. White solid. m.p.: 140.1-141.3 °C. **^{1}H NMR** (d_6-DMSO, 400 MHz) δ 7.83 (t, J = 0.7 Hz, 1H), 7.80 (bs, 1H), 7.41 (bs,1H), 7.14 (d, J = 3.4 Hz, 1H), 6.62 (q, J = 1.7 Hz, 1H); **^{13}C NMR** (d_6-DMSO, 100 MHz) δ 159.4, 148.0, 145.0, 113.6, 111.8.

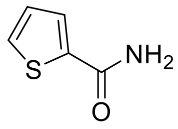

Thiophene-2-carboxamide (2b')[1]: Known compound. 101.6 mg, 80% yield. White solid. m.p.: 178.2-179.3 °C. **^{1}H NMR** (d_6-DMSO, 400 MHz) δ 7.95 (bs, 1H), 7.74 (s, 2H), 7.37 (bs, 1H), 7.13 (t, J = 3.9 Hz, 1H); **^{13}C NMR** (d_6-DMSO, 100 MHz) δ 162.8, 140.3, 130.9, 128.6, 127.8.

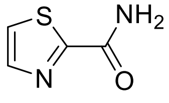

Thiazole-2-carboxamide (2c')[15]: Known compound. 78.1 mg, 61% yield. White solid. m.p.: 119.0-122.1 °C. **^{1}H NMR** (d_6-DMSO, 400 MHz) δ 8.21 (bs, 1H), 8.06 (d, *J* = 3.1 Hz, 1H), 8.03 (d, *J* = 3.1 Hz, 1H), 7.88 (bs, 1H); **^{13}C NMR** (d_6-DMSO, 100 MHz) δ 164.3, 160.9, 143.9, 125.9.

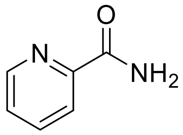

Picolinamide (2d')[1]: Known compound. 83.3 mg, 69% yield. White solid. m.p.: 106.3-108.8 °C. **^{1}H NMR** ($CDCl_3$, 400 MHz) δ 8.56 (d, *J* = 4.7 Hz, 1H), 8.19 (d, *J* = 7.8 Hz, 1H), 7.90 (bs, 1H), 7.83 (td, *J* = 7.7 and 1.0 Hz, 1H), 7.44-7.41 (m, 1H), 6.41 (bs, 1H); **^{13}C NMR** ($CDCl_3$, 100 MHz) δ 167.2, 149.7, 148.4, 137.4, 126.5, 122.5.

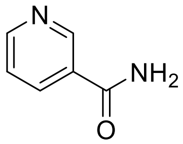

Nicotinamide (2e)'[1]: Known compound. 91.2 mg, 75% yield. White solid. m.p.: 134.4-137.5 °C. **^{1}H NMR** (d_6-DMSO, 400 MHz) δ 9.03 (d, *J* = 1.4 Hz, 1H), 8.69 (dd, *J* = 4.7 and 1.4 Hz, 1H), 8.22-8.19 (m, 1H), 8.18 (bs, 1H), 7.63 (bs, 1H), 7.49 (dd, *J* = 7.8 and 4.8 Hz, 1H); **^{13}C NMR** (d_6-DMSO, 100 MHz) δ 166.5, 151.9, 148.7, 135.2, 129.7, 123.4.

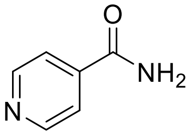

Isonicotinamide. **(2f')**[1]: Known compound. 104.8 mg, 86% yield. White solid. m.p.: 151.1-153.9 °C. **^{1}H NMR** (d_6-DMSO, 400 MHz) δ 8.64 (dd, *J* = 4.3 and 1.5 Hz, 2H), 7.78 (dd, *J* = 4.3 and 1.6 Hz, 2H); **^{13}C NMR** (d_6-DMSO, 100 MHz) δ 167.3, 149.6, 144.6, 123.1.

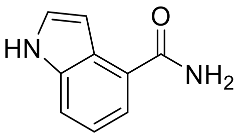

1*H*-Indole-4-carboxamide (2g')[6]: Known compound. 130.9 mg, 82% yield. Pale yellow solid. m.p.: 143.2-145.7 °C. **^{1}H NMR** (d_6-DMSO, 400 MHz) δ 11.3 (bs, 1H),

7.75 (bs, 1H), 7.58 (d, *J* = 8.0 Hz, 1H), 7.51 (d, *J* = 7.3 Hz, 1H), 7.46 (t, *J* = 2.8 Hz, 1H), 7.25 (bs, 1H), 7.16 (t, *J* = 7.7 Hz, 1H), 6.96 (t, *J* = 2.0 Hz, 1H); **^{13}C NMR** (d_6-DMSO, 100 MHz) δ 169.8, 136.6, 126.4, 126.2, 126.1, 120.0, 118.9, 114.2, 102.0.

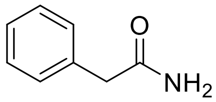

Phenylacetamide (2a'')[1]: Known compound. 109.4 mg, 81% yield. White solid. m.p.: 152.6-155.1 ℃. **^{1}H NMR** ($CDCl_3$, 400 MHz) δ 7.38-7.34 (m, 2H), 7.32-7.27 (m, 3H), 5.82 (bs, 1H), 5.41 (bs, 1H), 3.58 (s, 2H); **^{13}C NMR** ($CDCl_3$, 100 MHz) δ 173.7, 135.0, 129.5, 129.2, 127.6, 43.5.

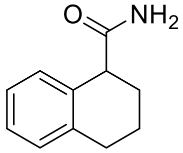

1,2,3,4-Tetrahydronaphthalene-1-carboxamide (2b'')[16]: Known compound. 130.3 mg, 74% yield. White solid. m.p.: > 300 ℃. **^{1}H NMR** (d_6-DMSO, 400 MHz) δ 7.49 (bs, 1H), 7.15-7.09 (m, 4H), 6.99 (bs, 1H), 3.64 (t, *J* = 6.8 Hz, 1H), 2.75-2.72 (m, 2H), 1.97-1.91 (m, 3H), 1.69-1.60 (m, 1H); **^{13}C NMR** (d_6-DMSO, 100 MHz) δ 176.2, 137.0, 135.3, 128.9, 128.4, 126.0, 125.4, 45.0, 28.8, 26.9, 20.6.

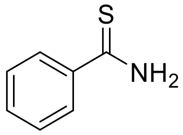

Benzothioamide (3a)[17]: Known compound. 99.8 mg, 73% yield. Yellow solid. m.p.: 114.5-115.7 ℃. **^{1}H NMR** ($CDCl_3$, 400 MHz) δ 7.95 (bs, 1H), 7.87-7.86 (m, 1H), 7.85-7.84 (m, 1H), 7.52-7.48 (m, 1H), 7.42-7.37 (m, 2H), 7.30 (bs, 1H); **^{13}C NMR** ($CDCl_3$, 100 MHz) δ 202.9, 139.2, 132.1, 128.6, 127.0.

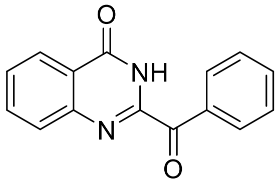

2-Benzoylquinazolin-4(3*H*)-one (3c)[18]: Known compound. 188.0 mg, 75% yield. White solid. m.p.: 182.5-183.9 ℃. **^{1}H NMR** ($CDCl_3$, 400 MHz) δ 10.5 (bs, 1H), 8.52-8.49 (m, 2H), 8.39 (dd, *J* = 7.9 and 1.4 Hz, 1H), 7.93-7.91 (m, 1H), 7.86-7.82 (m,

1H), 7.69-7.61 (m, 2H), 7.56-7.52 (m, 2H); **^{13}C NMR** ($CDCl_3$, 100 MHz) δ 185.7, 161.1, 147.6, 146.1, 134.9, 134.4, 134.1, 131.9, 129.5 x 2, 128.5, 127.0, 123.4.

9. References

[1] H. Chen, W. Dai, Y. Chen, Q. Xu, J. Chen, L. Yu, Y. Zhao, M. Ye and Y. Pan, *Green Chem.*, 2014, **16**, 2136-2141.

[2] F.-L. Yang, X. Zhu, D.-K. Rao, X.-N. Cao, K. Li, Y. Xu, X.-Q. Hao and M.-P. Song, *RSC Adv.*, 2016, **6**, 37093-37098.

[3] M. A. Marx, A.-L. Grillot, C.-T. Louer, K. A. Beaver and P. A. Bartlett, *J. Am. Chem. Soc.*, 1997, **119**, 6153-6167.

[4] C. Battilocchio, J. M. Hawkins and S. V. Ley, *Org. Lett.*, 2014, **16**, 1060-1063.

[5] T. Deng and C.-Z. Wang, *ChemCatChem*, 2017, **9**, 1349-1353.

[6] R. Jamatia, A. Gupta and A. K. Pal, *ACS Sustainable Chem. Eng.*, 2017, **5**, 7604-7612.

[7] X. Cheng, S. Vellalath, R. Goddard and B. List, *J. Am. Chem. Soc.*, 2008, **130**, 15786-15787.

[8] T. Tu, Z. Wang, Z. Liu, X. Feng and Q. Wang, *Green Chem.*, 2012, **14**, 921-924.

[9] G. Wang, X. Chen, Y. Deng, Z. Li and X. Xu, *J. Agric. Food Chem.*, 2015, **63**, 6883-6889.

[10] R. J. Rahaim Jr and R. E. Maleczka Jr, *Synthesis*, 2006, 3316-3340.

[11] T. E. Schmid, A. Gómez-Herrera, O. Songis, D. Sneddon, A. Révolte, F. Nahra and C. S. J. Cazin, *Catal. Sci. Technol.*, 2015, **5**, 2865-2868.

[12] A. D. Robert, P. D. Anthony, S. B. L. Christa, W. Anthony, P. Manoussos and E. M. Paul, *PCT Int. Appl.* 2000039125, 2000.

[13] R. Peters and D. F. Fischer, *Org. Lett.*, 2005, **7**, 4137-4140.

[14] A. Cavarzan, A. Scarso and G. Strukul, *Green Chem.*, 2012, **14**, 921-924.

[15] M. A. Schade, G. Manolikakes and P. Knochel, *Org. Lett.*, 2010, **12**, 3648-3650.

[16] Y. K. Chen, T. Bonaldi, A. Cuomo, J. R. Del Rosario, D. J. Hosfield, T. Kanouni, S. Kao, C. Lai, N. A. Lobo, J. Matuszkiewicz, A. McGeehan, S. M. O'Connell, L. Shi, J. A. Stafford, R. K. Stansfield, J. M. Veal, M. S. Weiss, N. Y. Yuen and M. B. Wallace, *ACS Med. Chem. Lett.*, 2017, **8**, 869-874.

[17] Z. Kaleta, B. T. Makowski, T. Soós and R. Dembinski, *Org. Lett.*, 2006, **8**, 1625-1628.

[18] Y.-P. Zhu, Z. Fei, M.-C. Liu, F.-C. Jia and A.-X. Wu, *Org. Lett.*, 2013, **15**, 378-381.

10. Copies of NMR spectra

^{1}H NMR of product 2a in $CDCl_3$ (400 MHz)

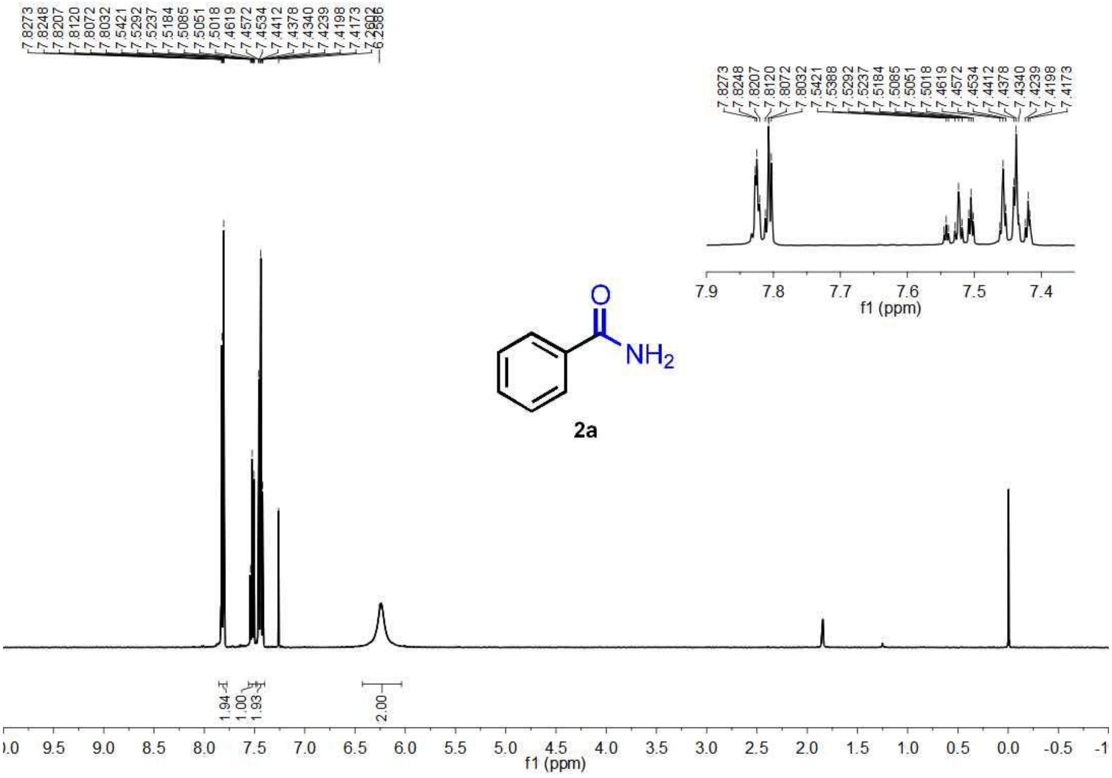

^{13}C NMR of product 2a in $CDCl_3$ (100 MHz)

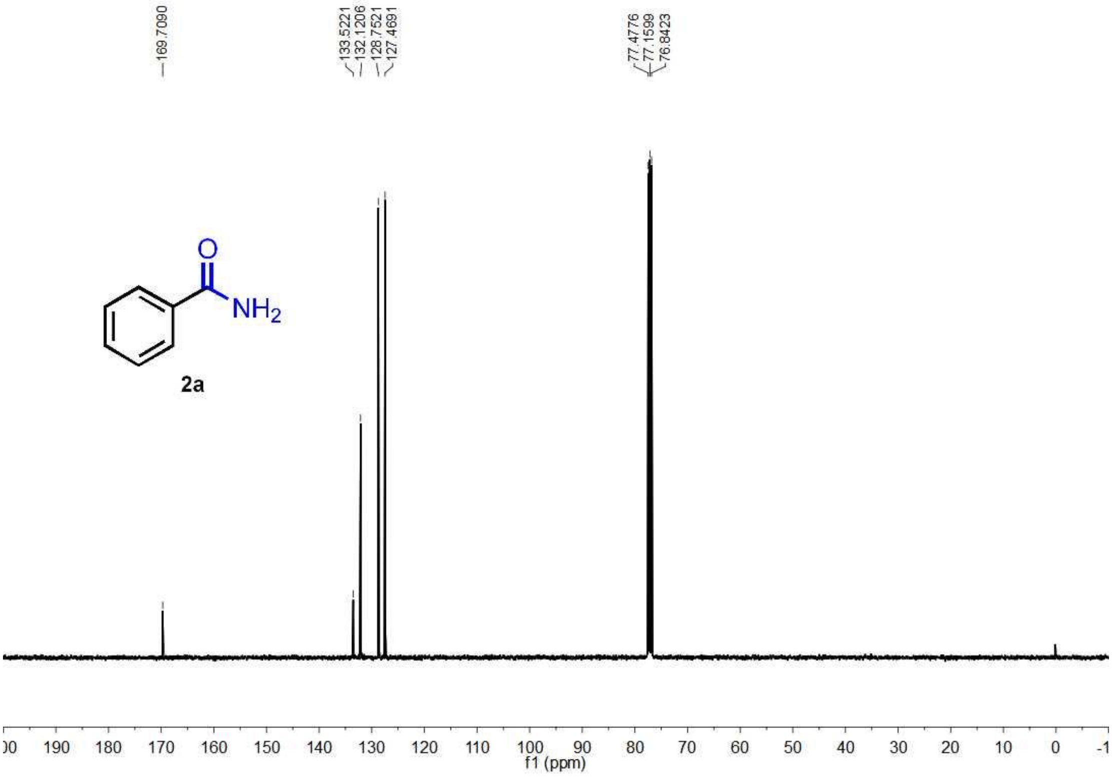

^{1}H NMR of product 2b in $CDCl_3$ (400 MHz)

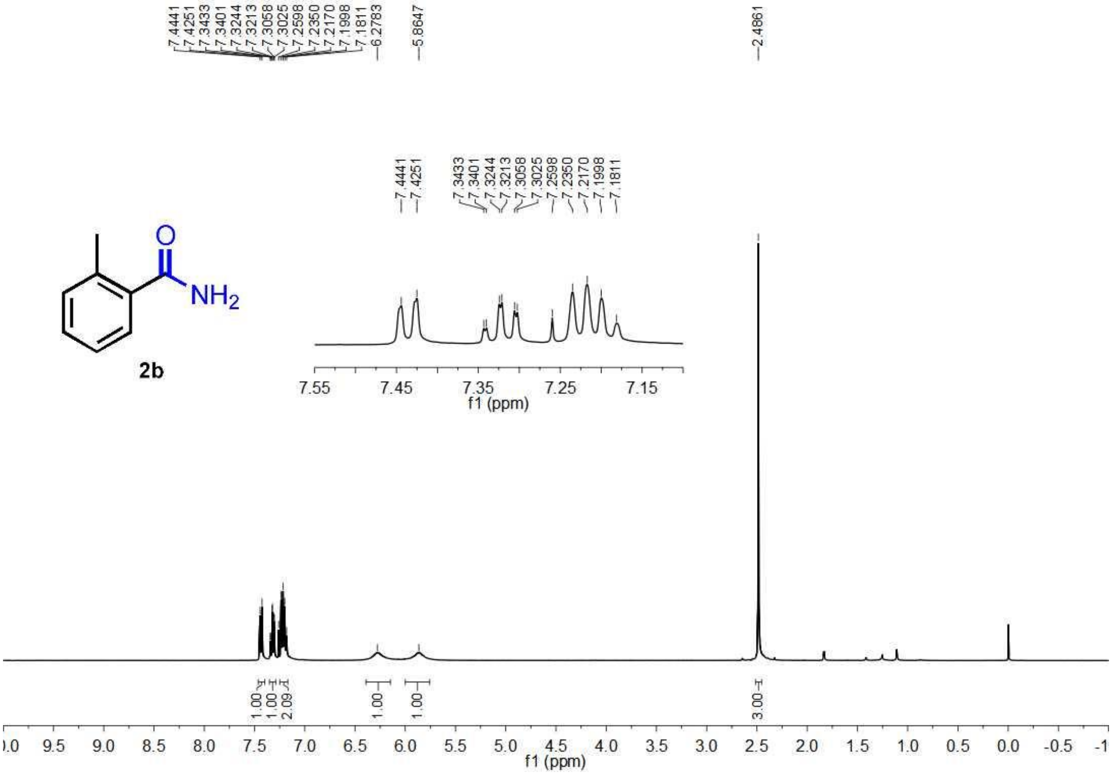

^{13}C NMR of product 2b in $CDCl_3$ (100 MHz)

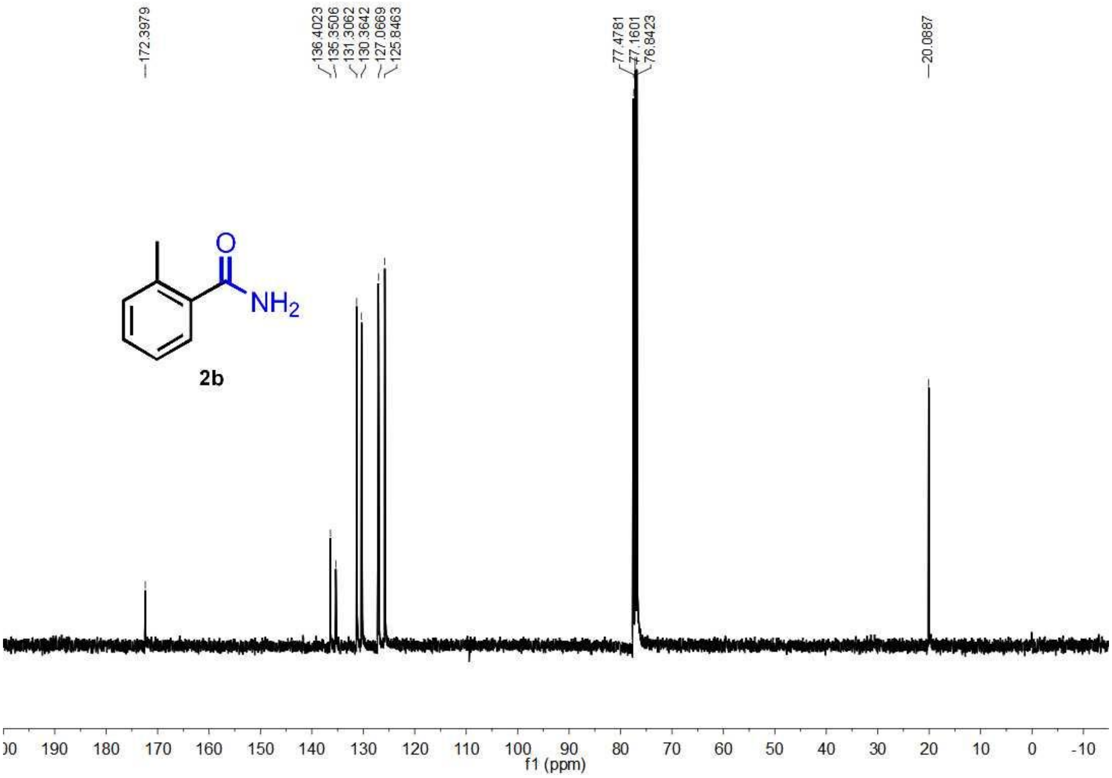

^{1}H NMR of product 2c in $CDCl_3$ (400 MHz)

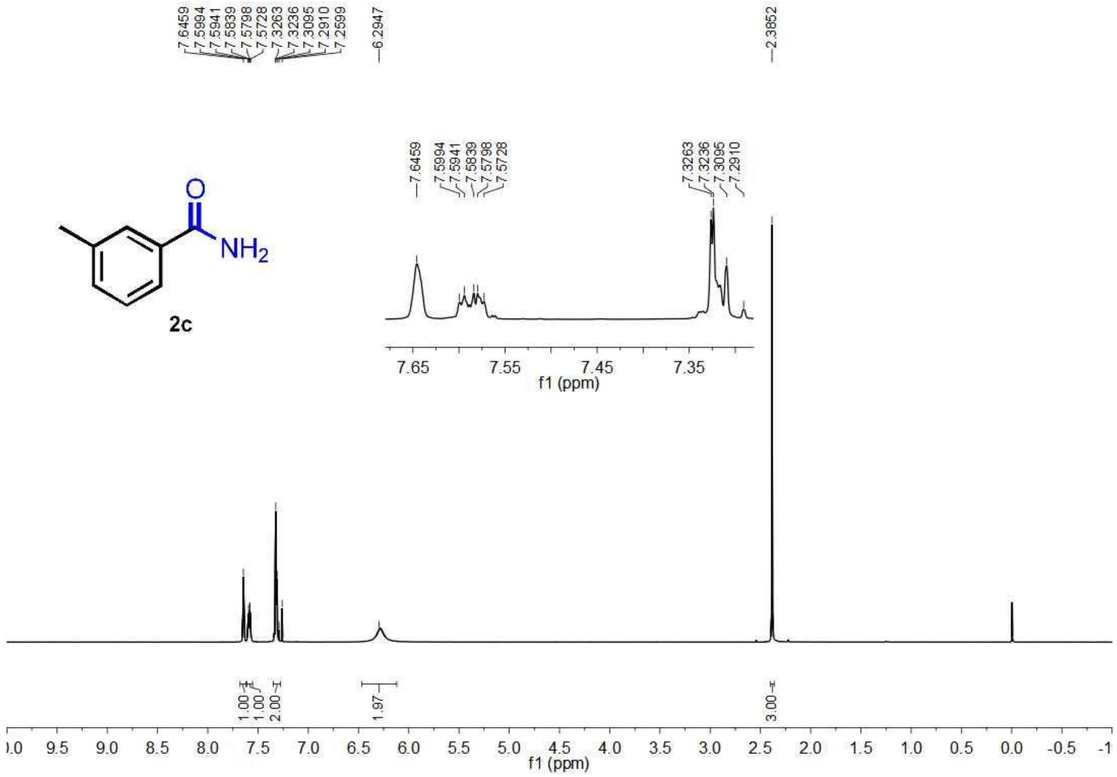

^{13}C NMR of product 2c in $CDCl_3$ (100 MHz)

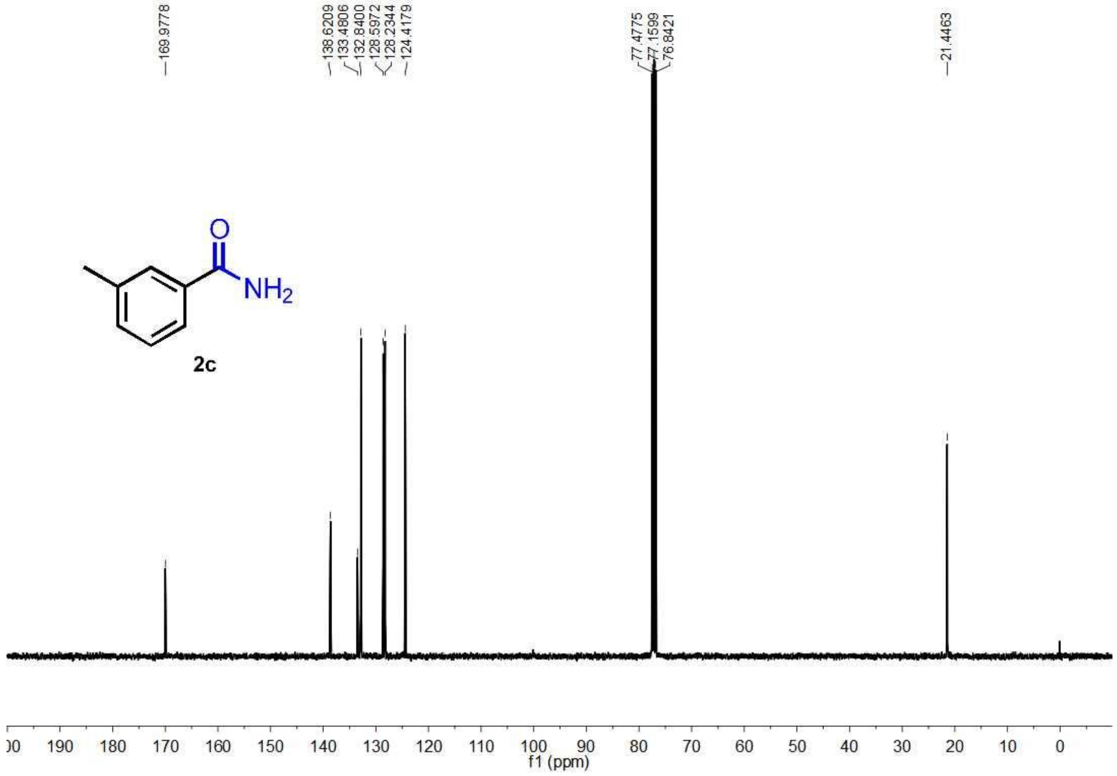

^{1}H NMR of product 2d in $CDCl_3$ (400 MHz)

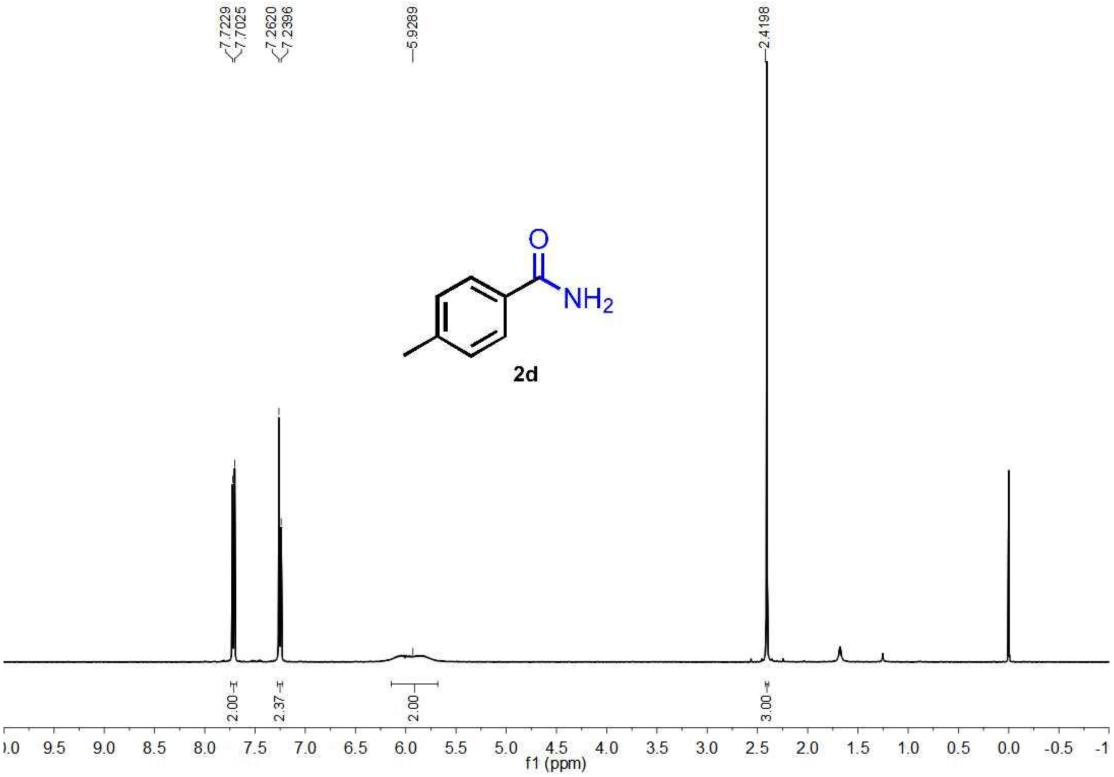

^{13}C NMR of product 2d in $CDCl_3$ (100 MHz)

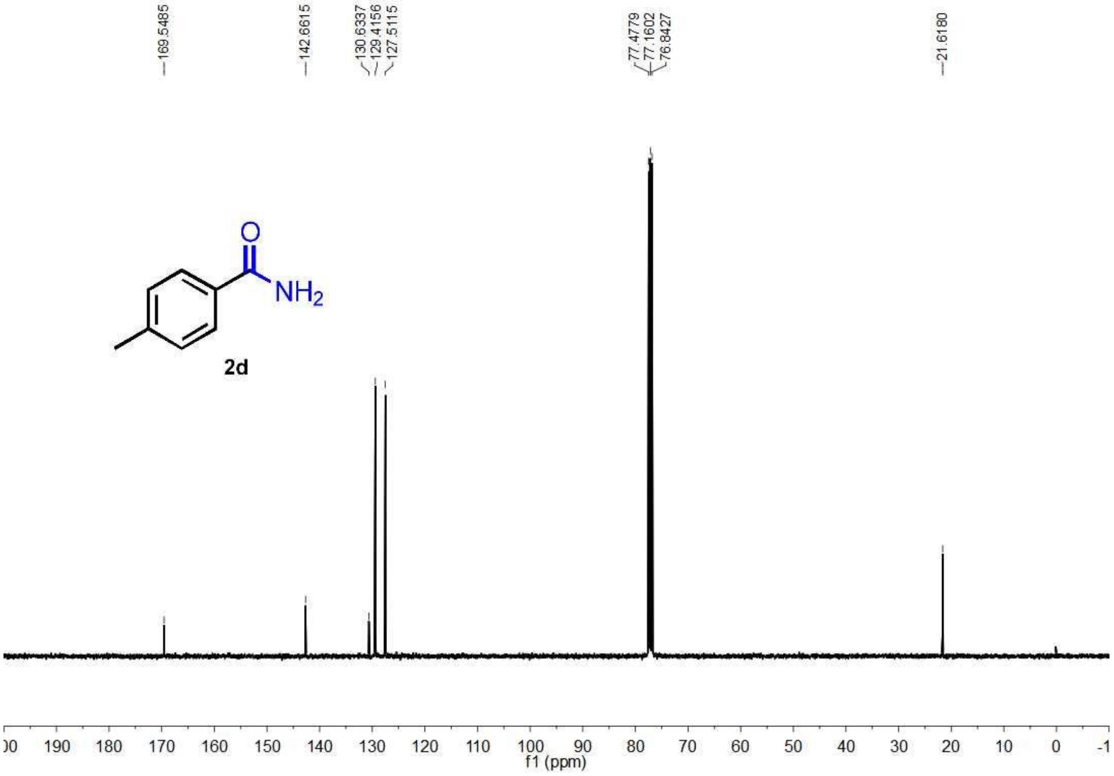

^{1}H NMR of product 2e in $CDCl_3$ (400 MHz)

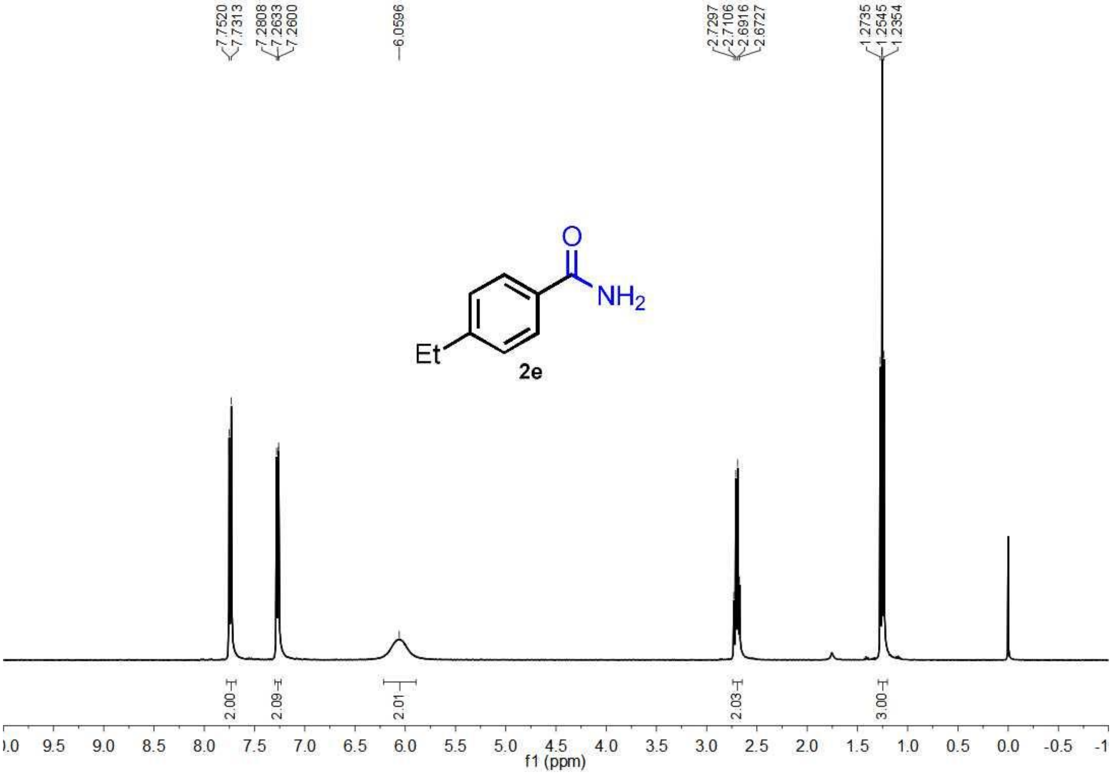

^{13}C NMR of product 2e in $CDCl_3$ (100 MHz)

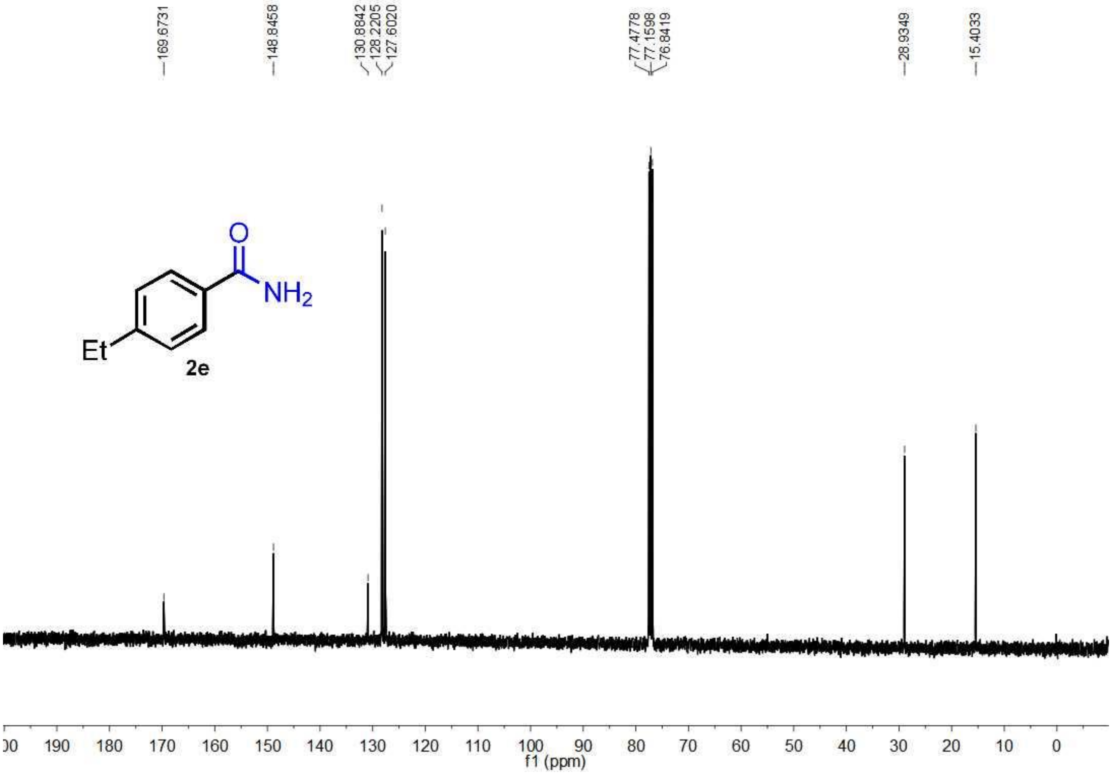

^{1}H NMR of product 2f in d_6-DMSO (400 MHz)

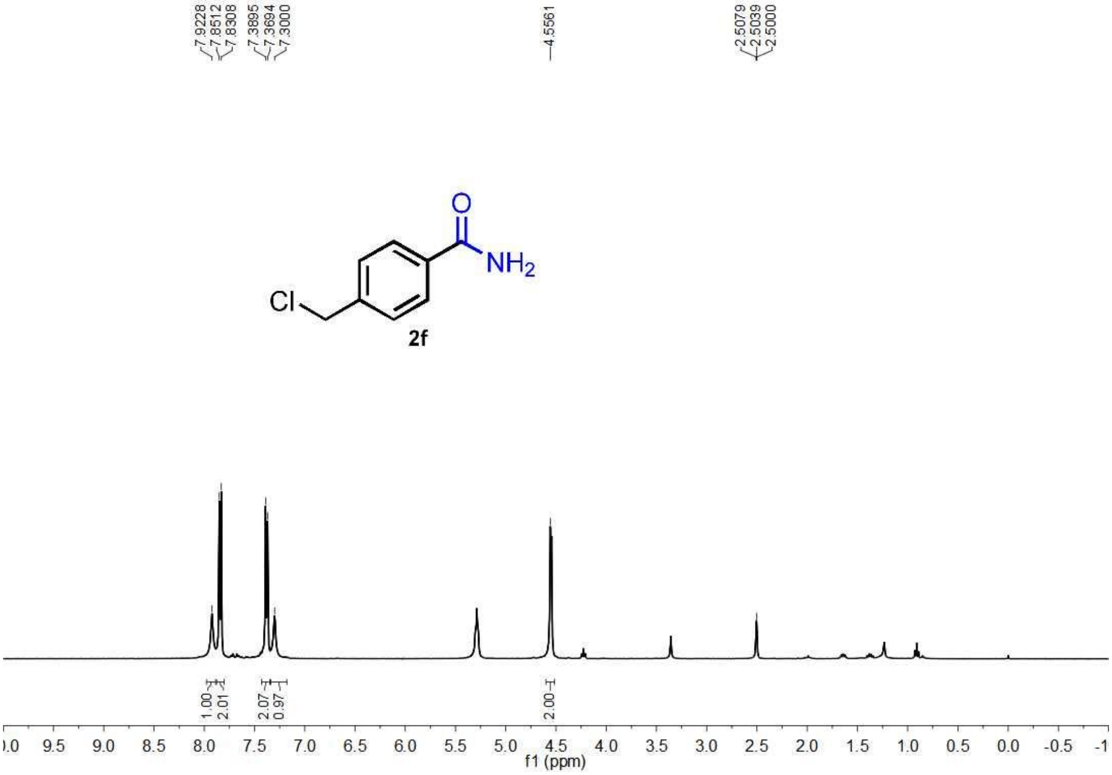

^{13}C NMR of product 2f in d_6-DMSO (100 MHz)

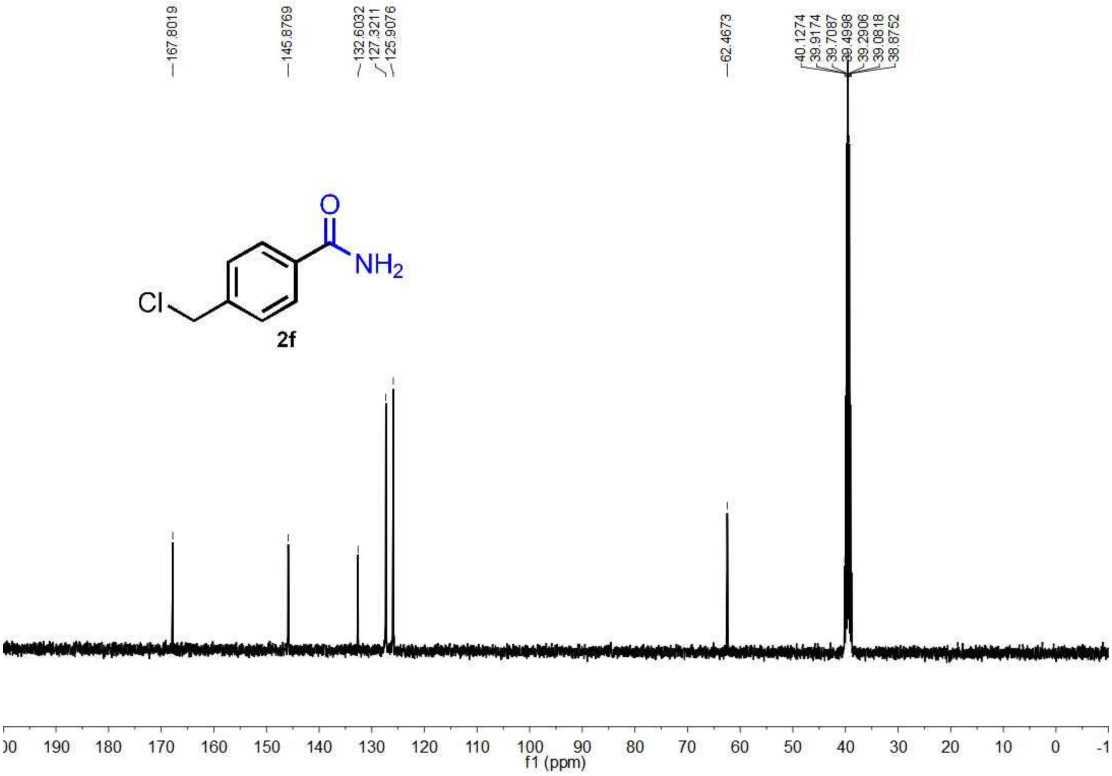

^{1}H NMR of product 2g in d_6-DMSO (400 MHz)

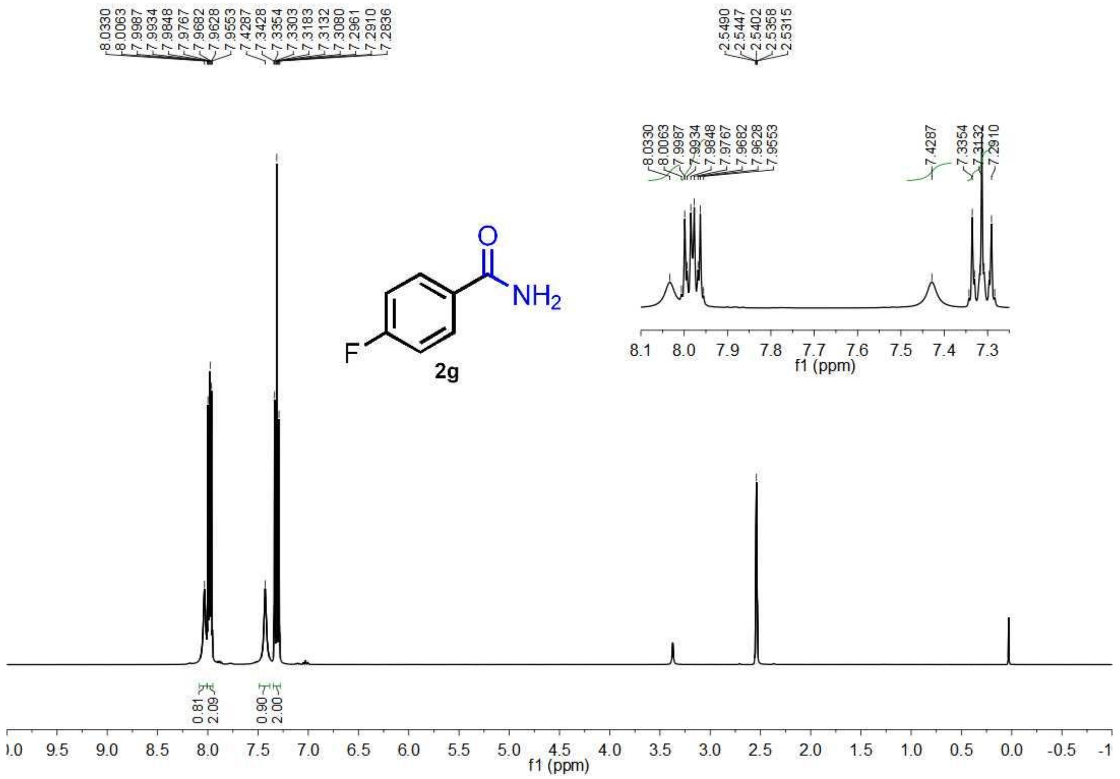

^{13}C NMR of product 2g in d_6-DMSO (100 MHz)

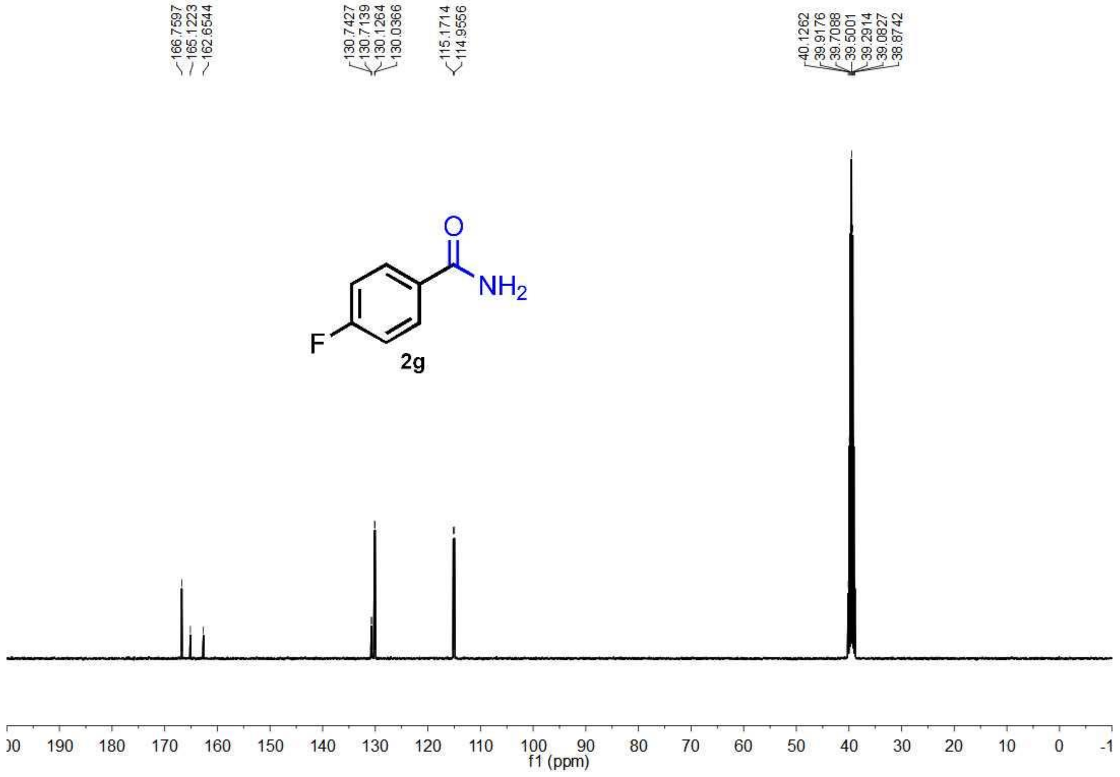

^{19}F NMR of product 2g in d_6-DMSO (376 MHz)

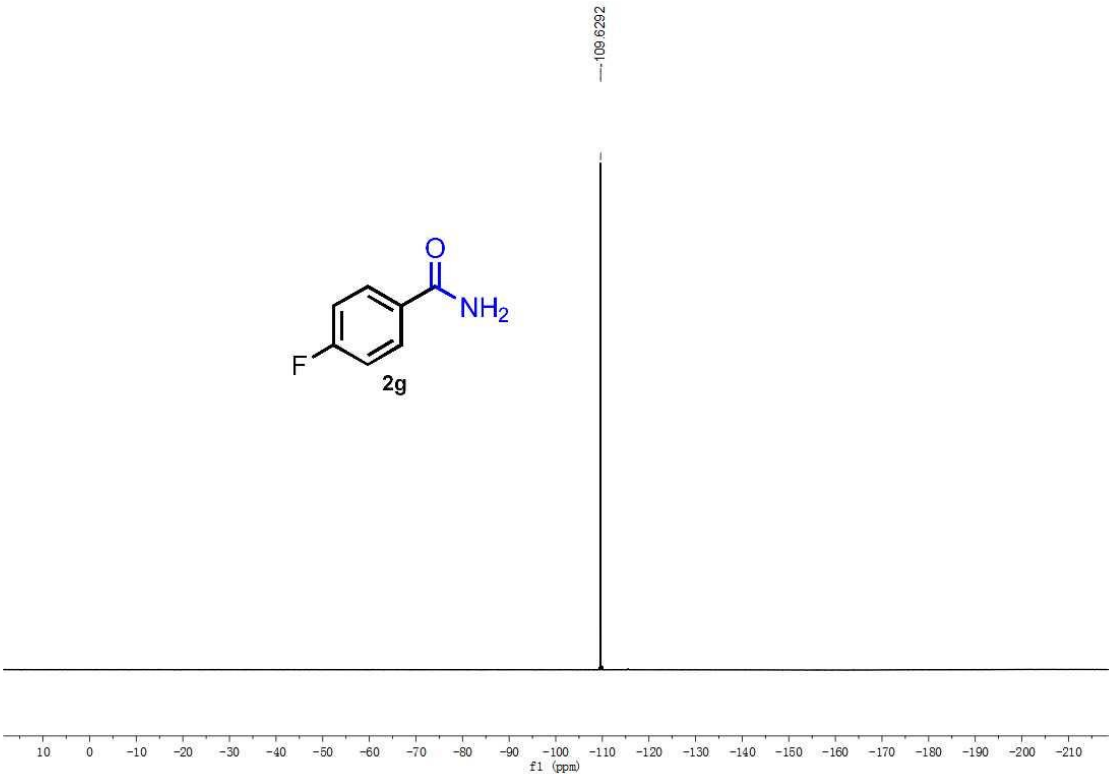

^{1}H NMR of product 2h in $CDCl_3$ (400 MHz)

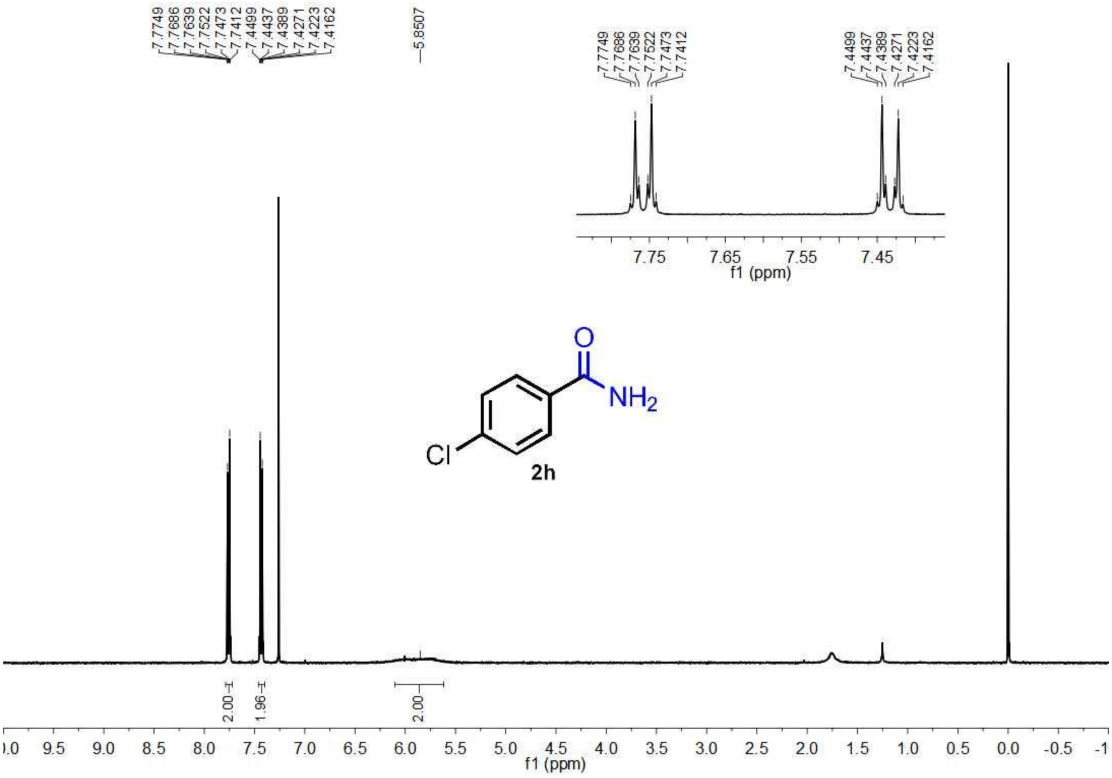

^{13}C NMR of product 2h in $CDCl_3$ (100 MHz)

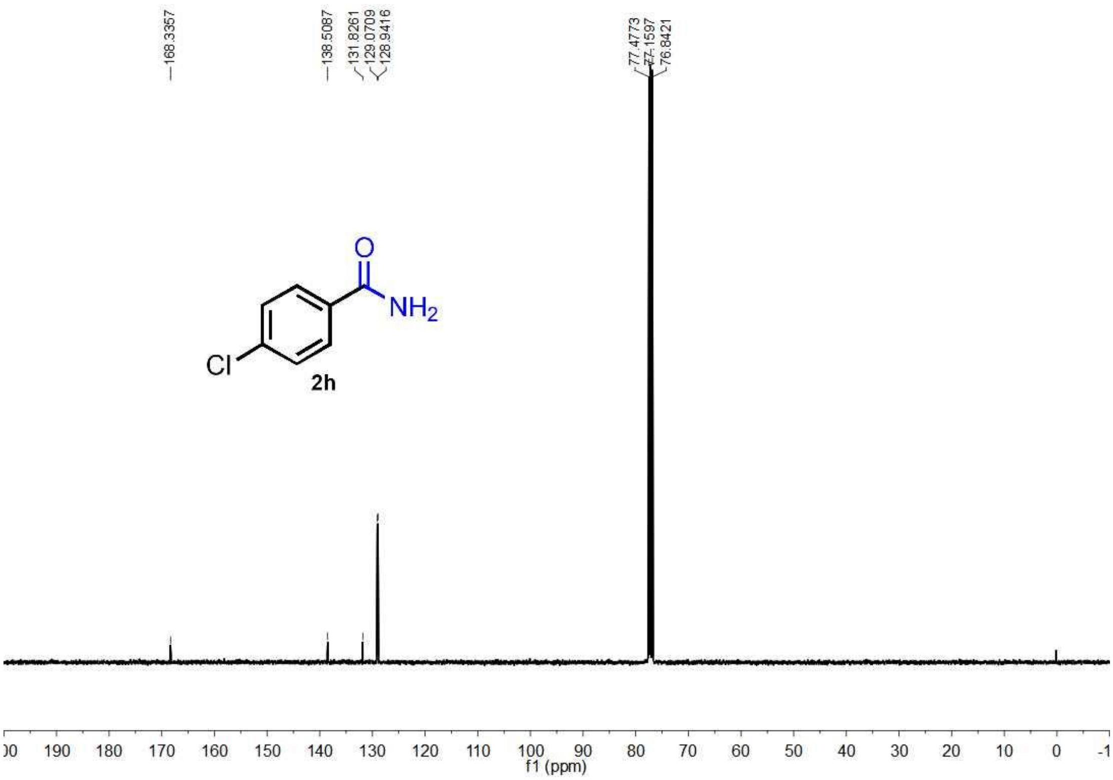

^{1}H NMR of product 2i in d_6-DMSO (400 MHz)

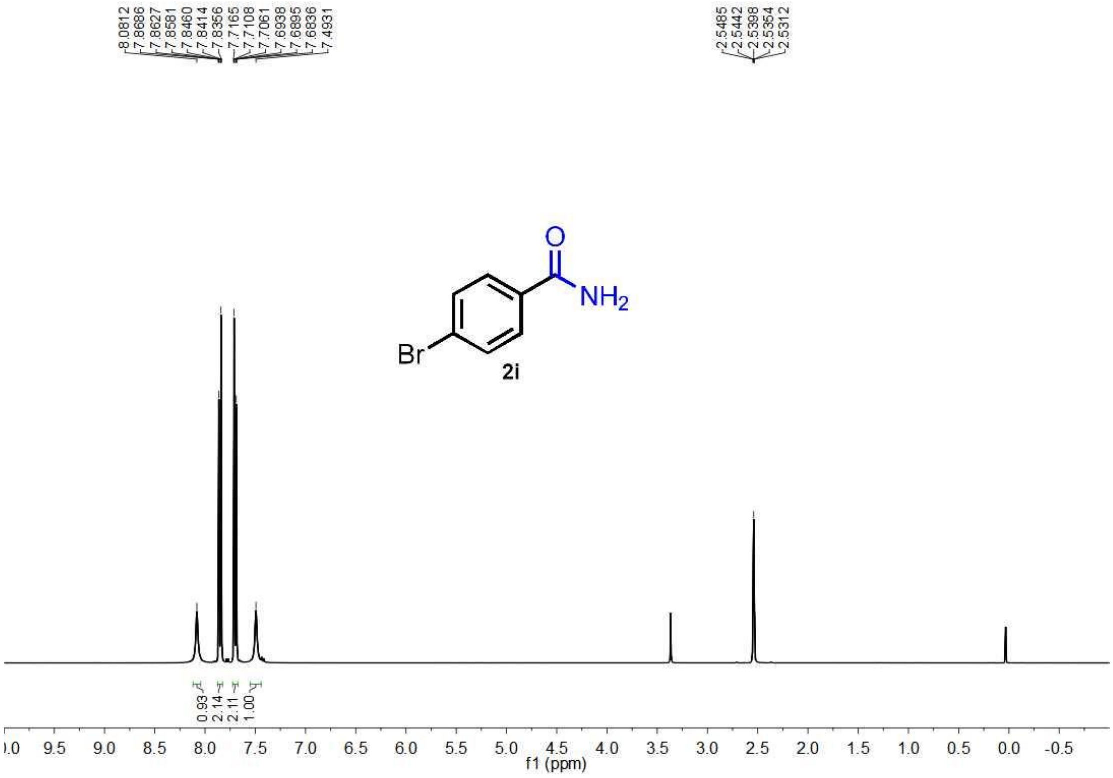

^{13}C NMR of product 2i in *d₆*-DMSO (100 MHz)

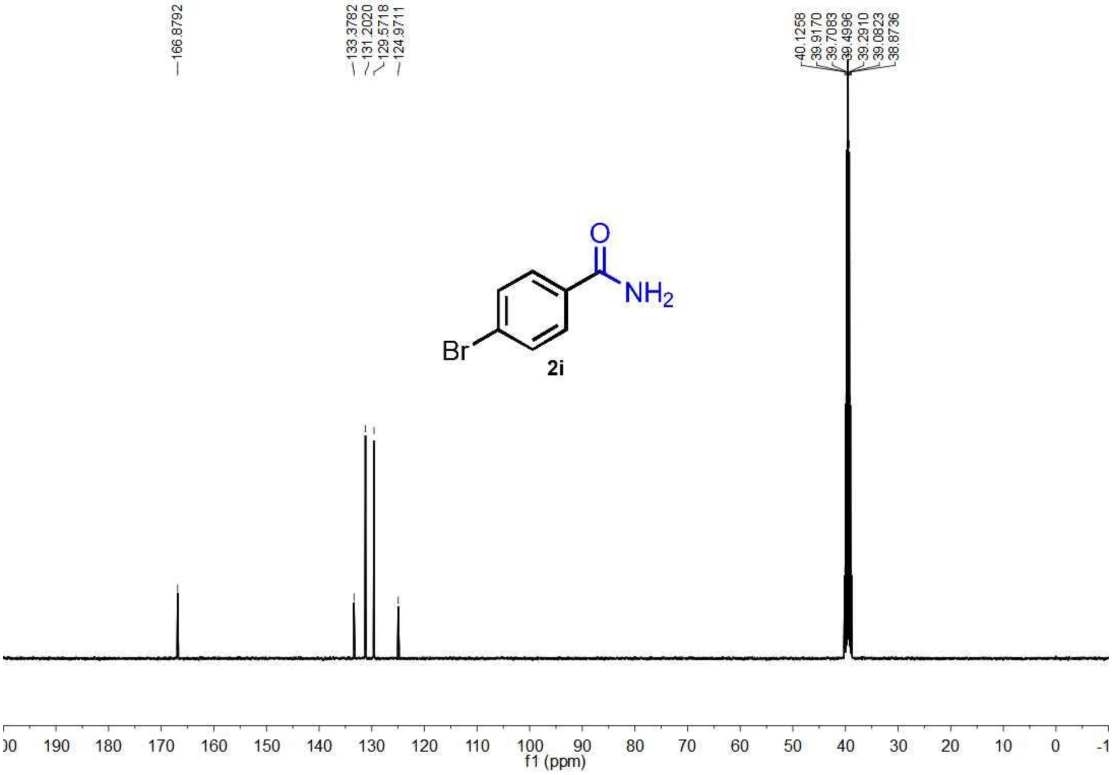

^{1}H NMR of product 2j in *d₆*-DMSO (400 MHz)

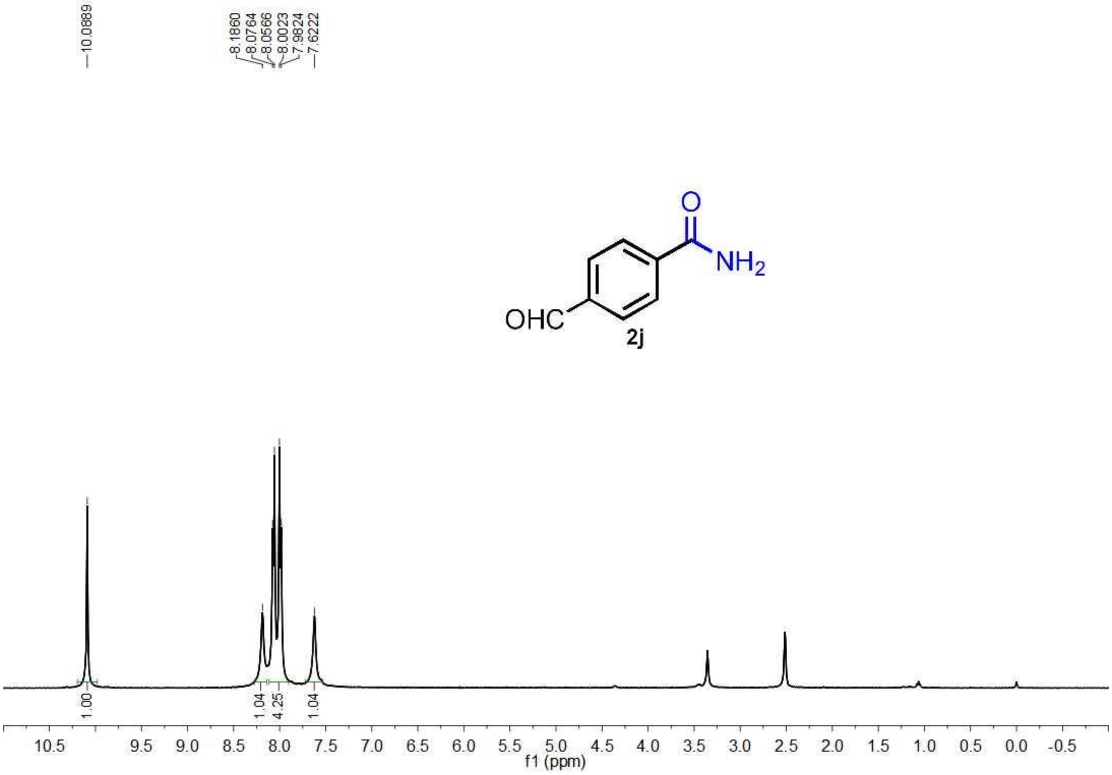

^{13}C NMR of product 2j in d_6-DMSO (100 MHz)

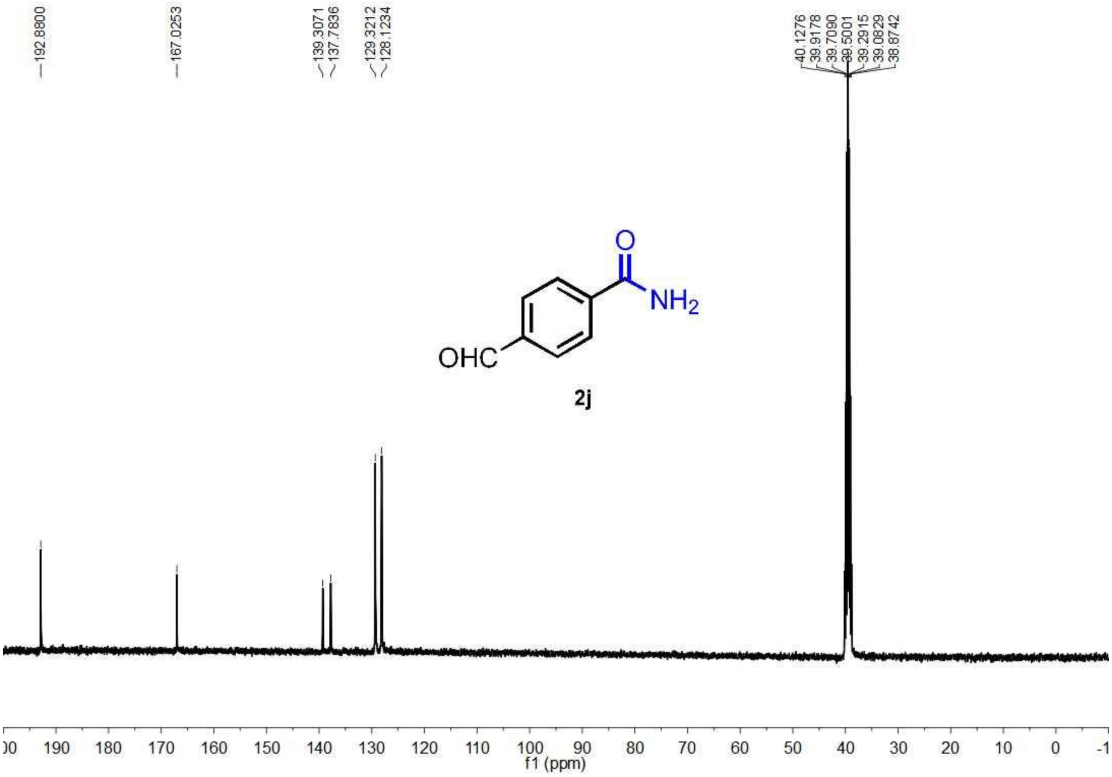

^{1}H NMR of product 2k in d_6-DMSO (400 MHz)

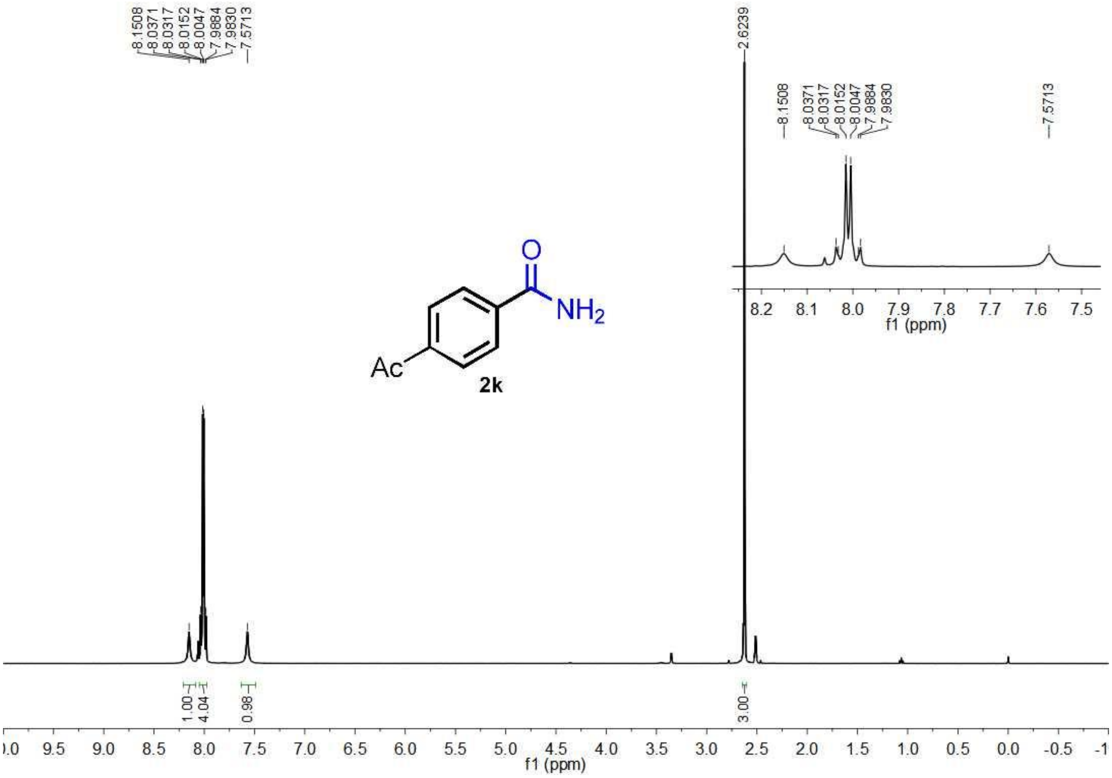

^{13}C NMR of product 2k in d_6-DMSO (100 MHz)

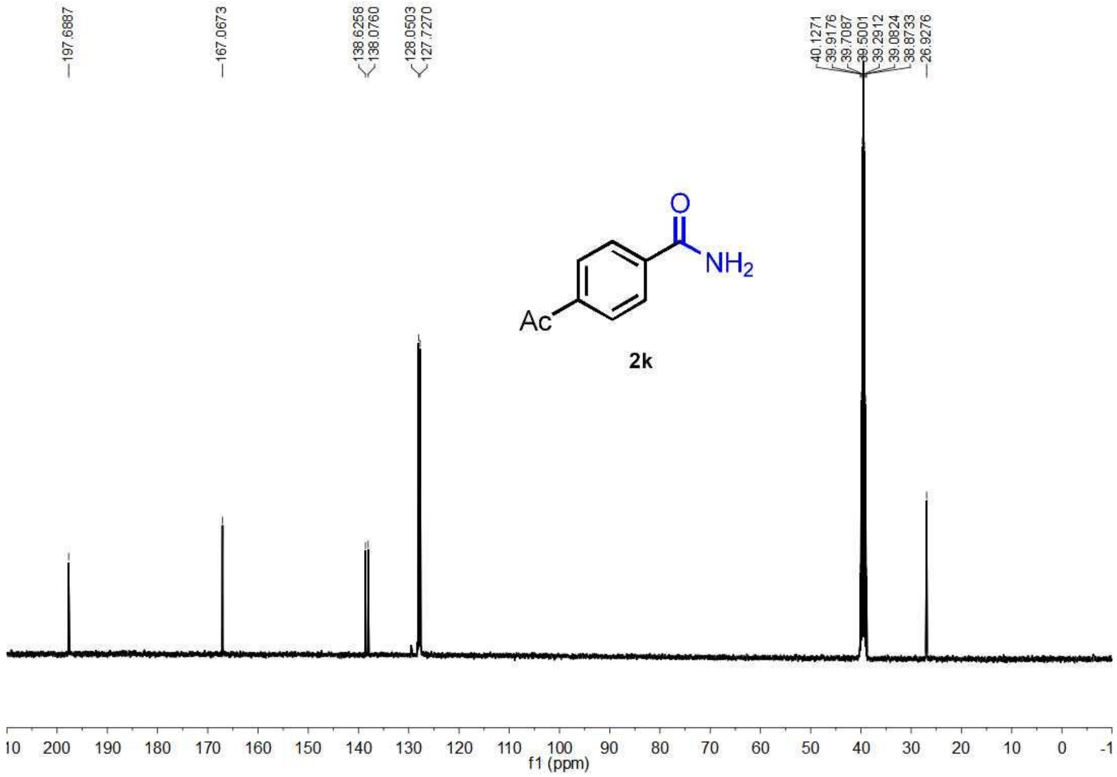

^{1}H NMR of product 2l in d_6-DMSO (400 MHz)

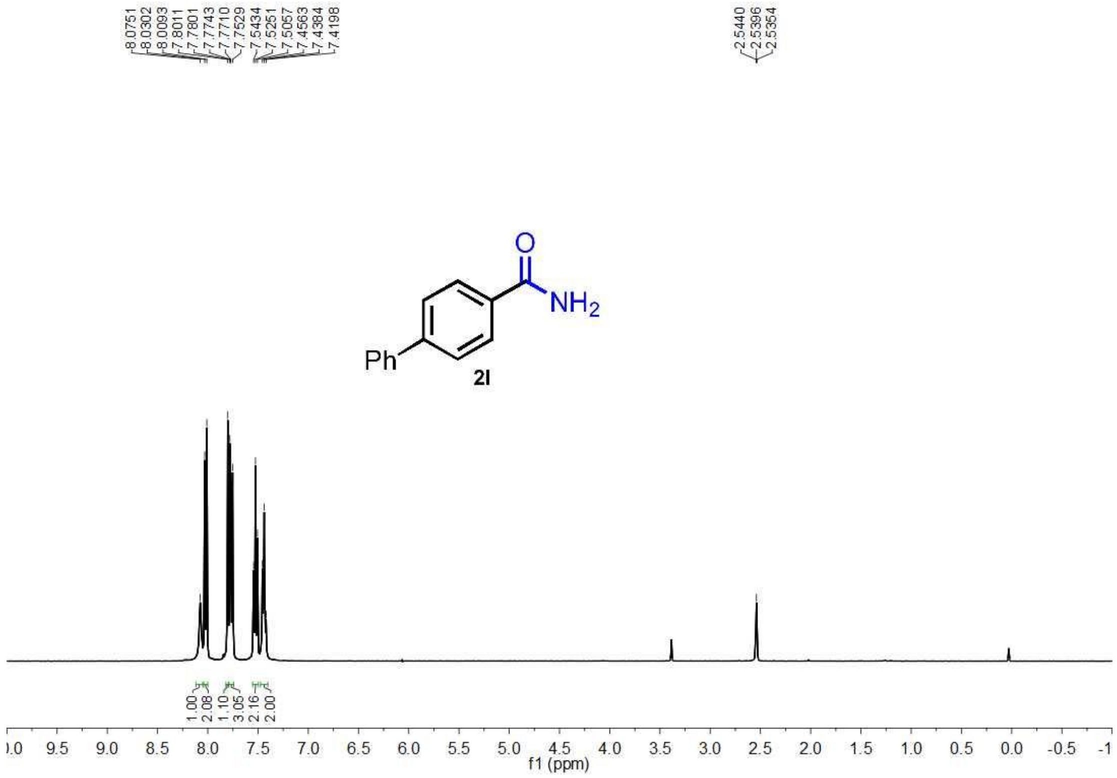

^{13}C NMR of product 2l in *d_6*-DMSO (100 MHz)

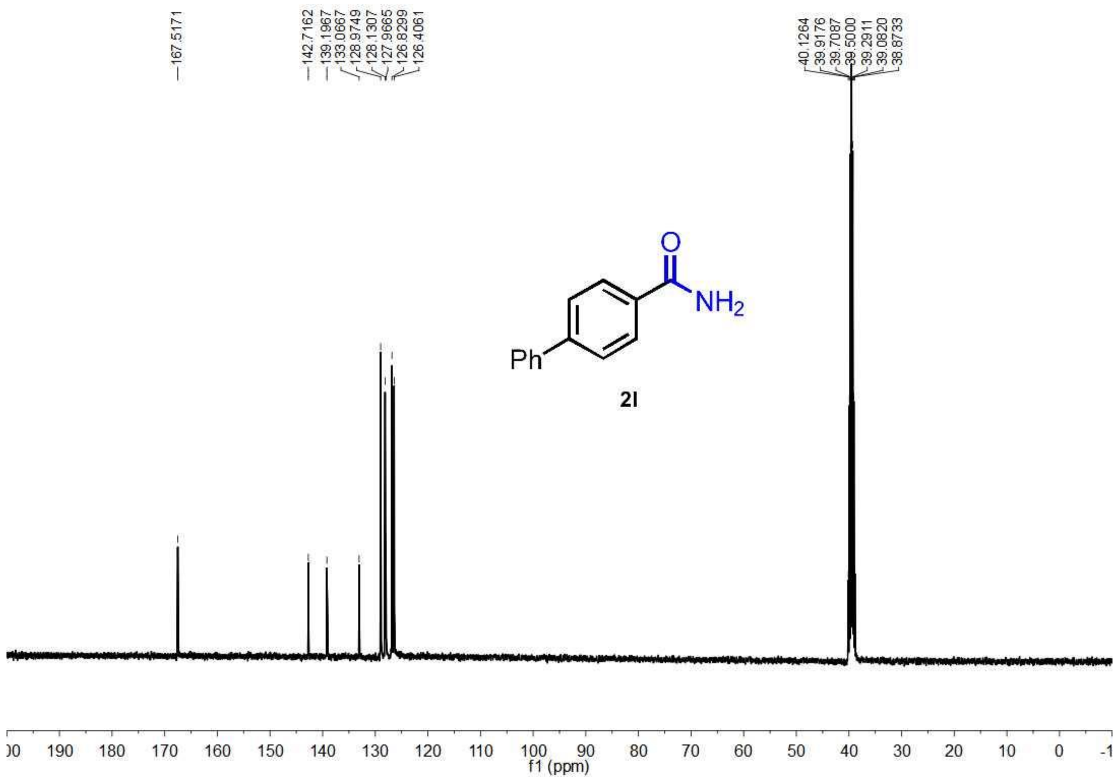

^{1}H NMR of product 2m in *d_6*-DMSO (400 MHz)

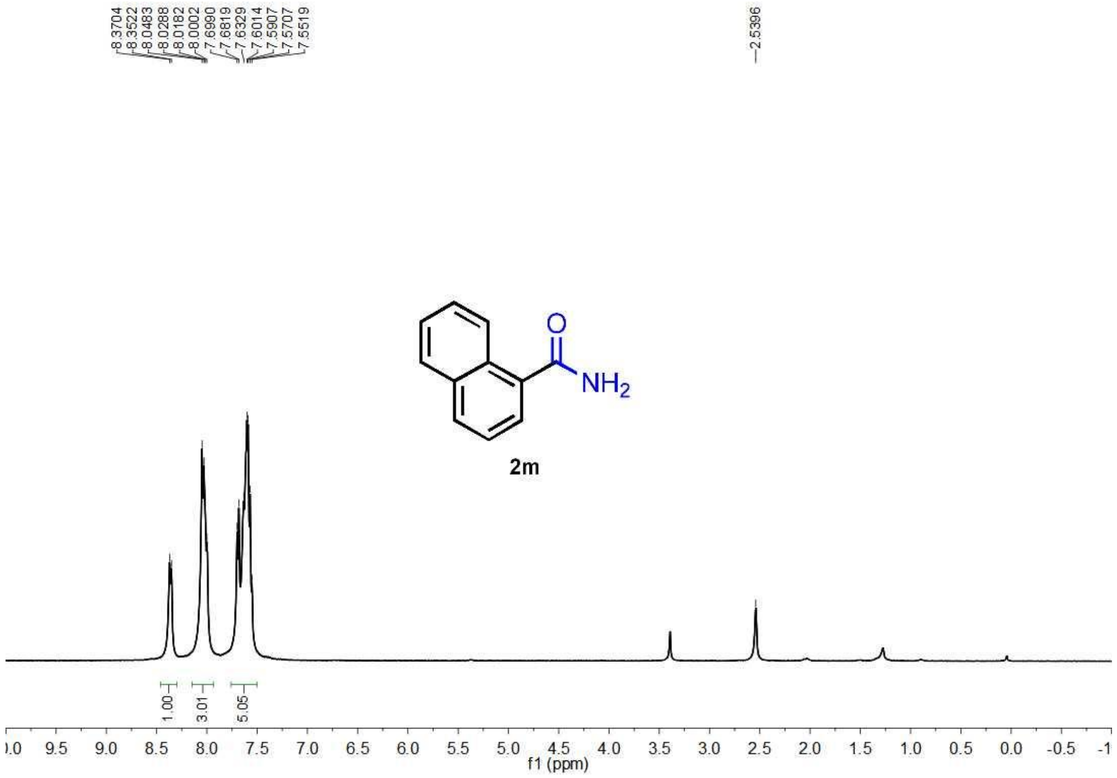

^{13}C NMR of product 2m in *d_6*-DMSO (100 MHz)

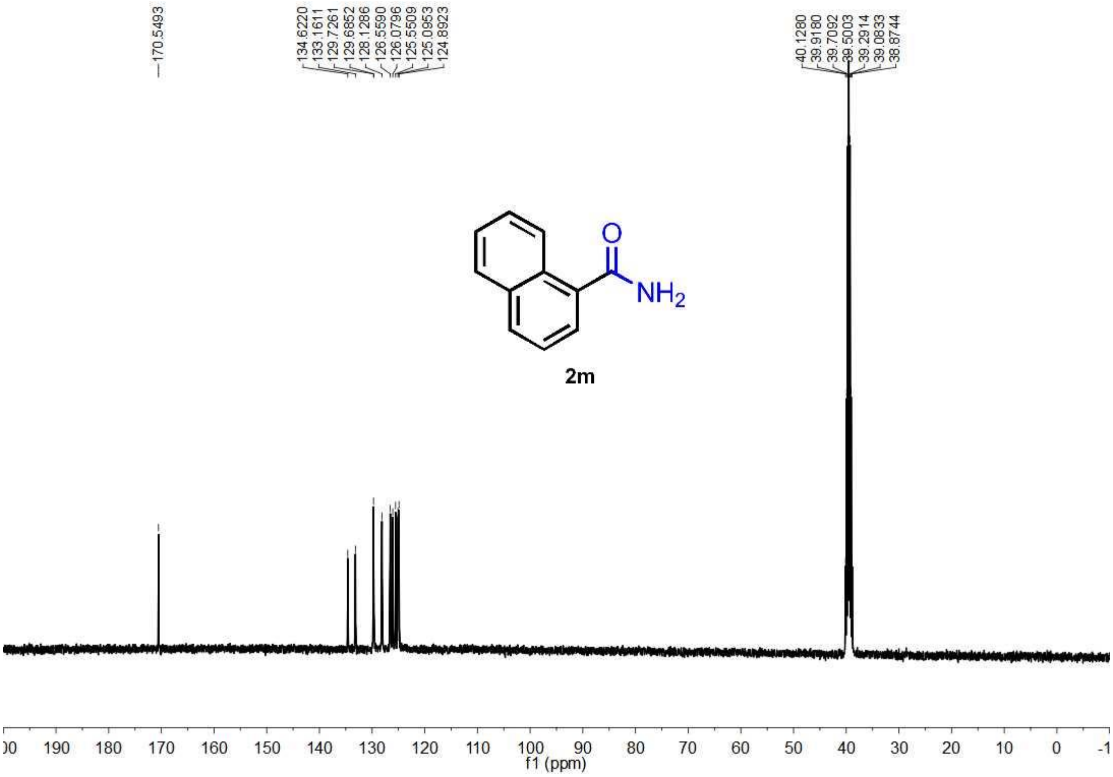

^{1}H NMR of product 2n in *d_6*-DMSO (400 MHz)

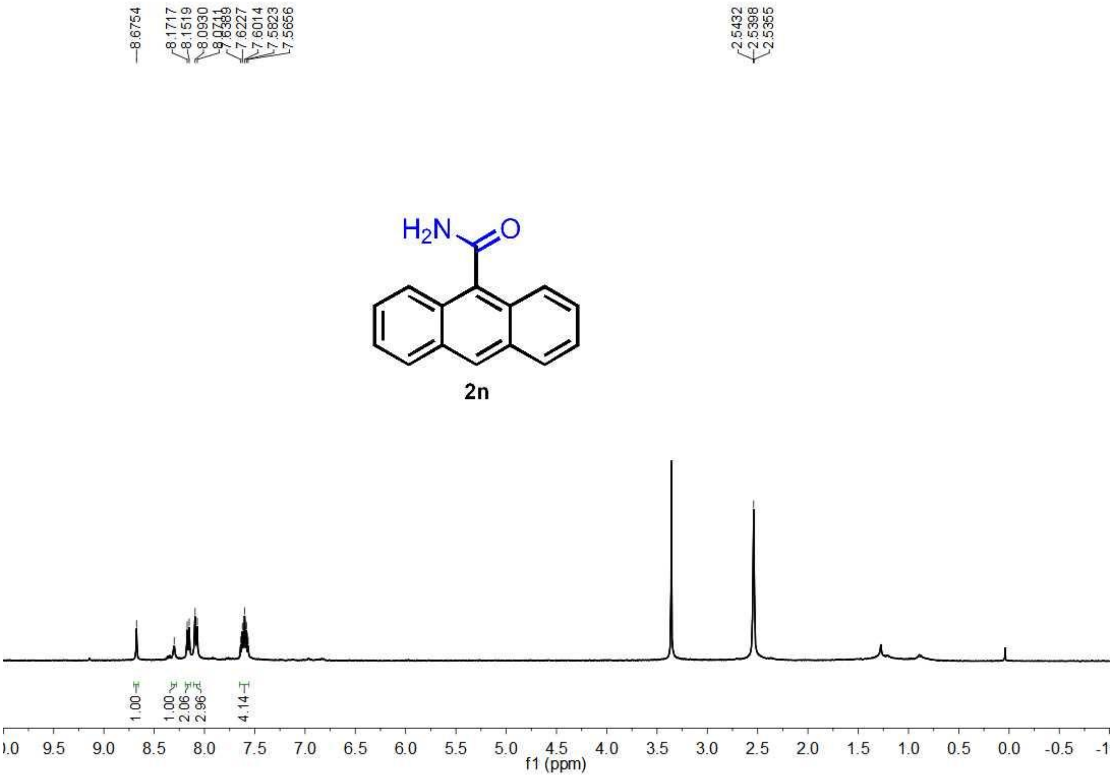

^{13}C NMR of product 2n in *d_6*-DMSO (100 MHz)

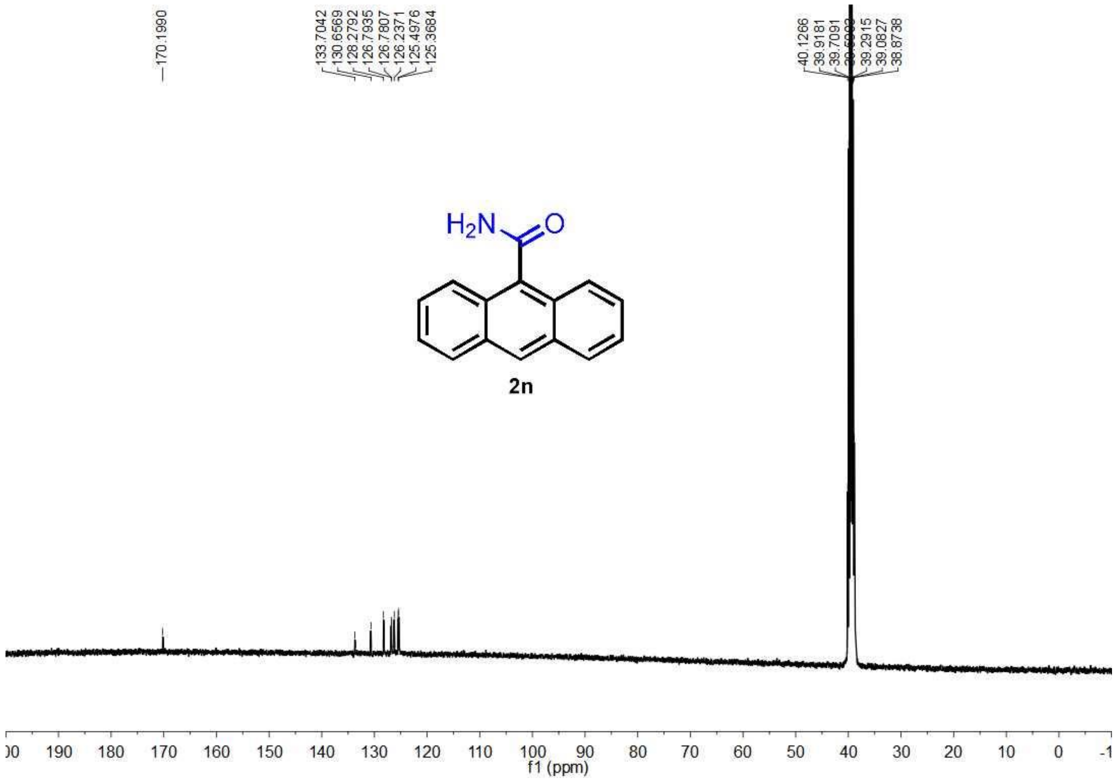

^{1}H NMR of product 2o in $CDCl_3$ (400 MHz)

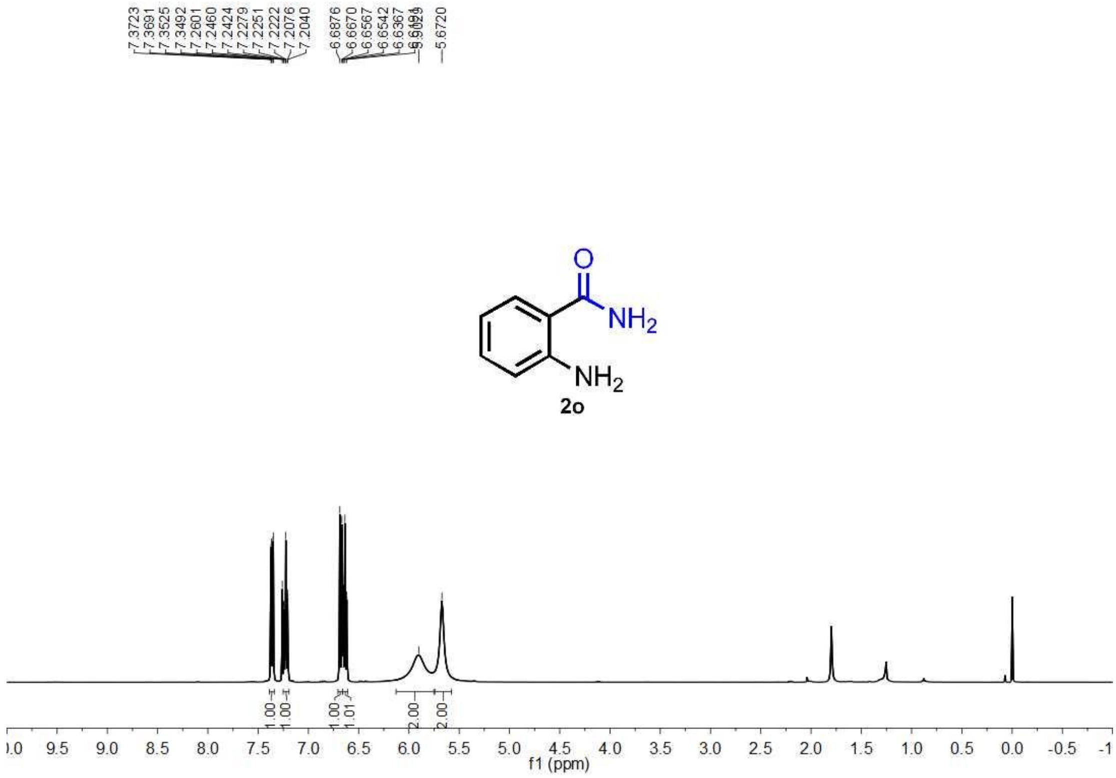

^{13}C NMR of product 2o in $CDCl_3$ (100 MHz)

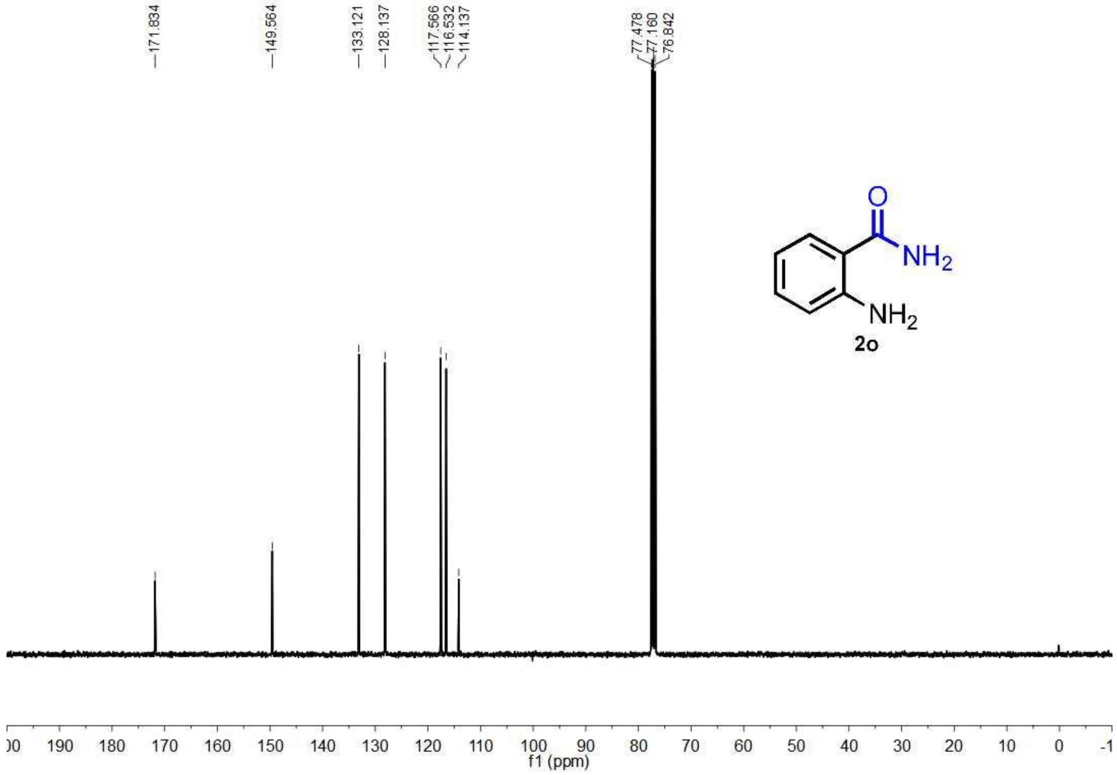

^{1}H NMR of product 2p in d_6-DMSO (400 MHz)

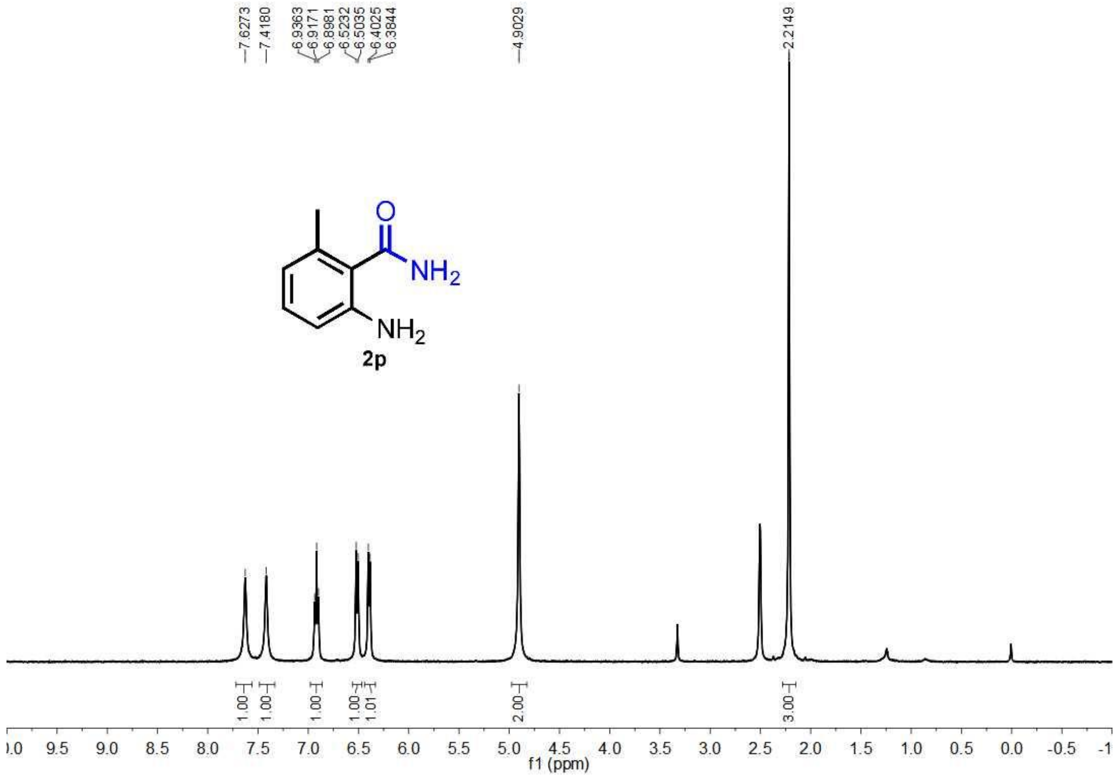

^{13}C NMR of product 2p in d_6-DMSO (100 MHz)

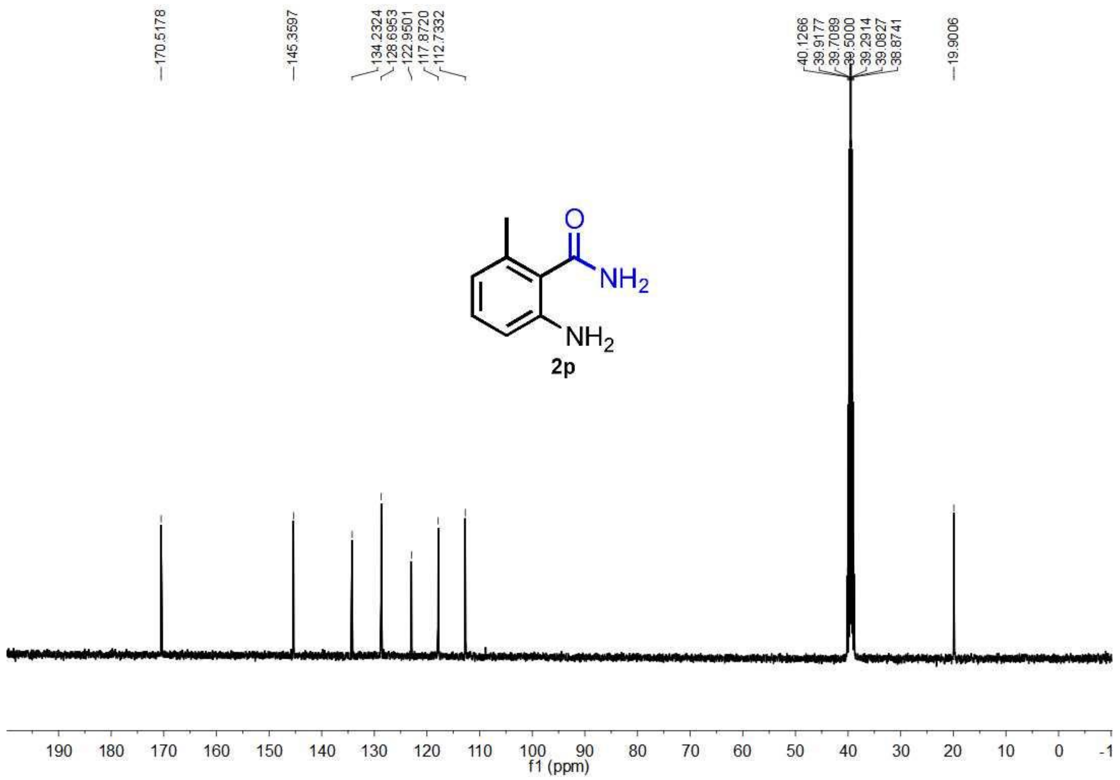

^{1}H NMR of product 2q in d_6-DMSO (400 MHz)

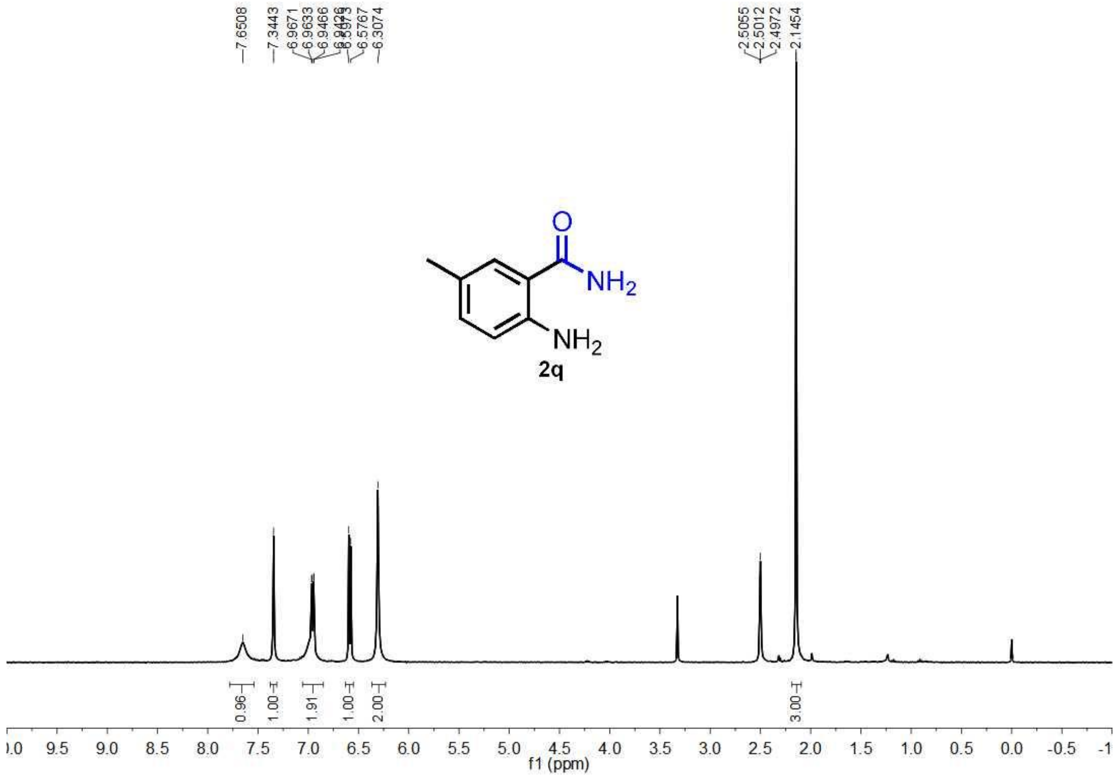

^{13}C NMR of product 2q in *d6*-DMSO (100 MHz)

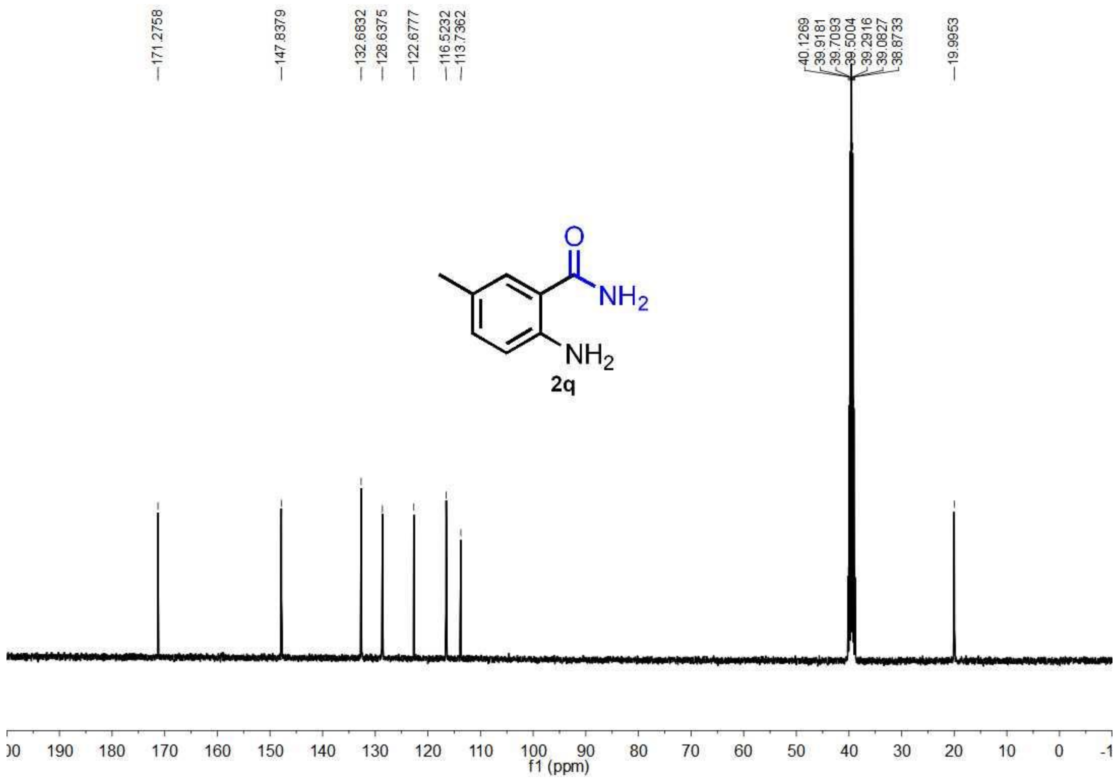

^{1}H NMR of product 2r in *d6*-DMSO (400 MHz)

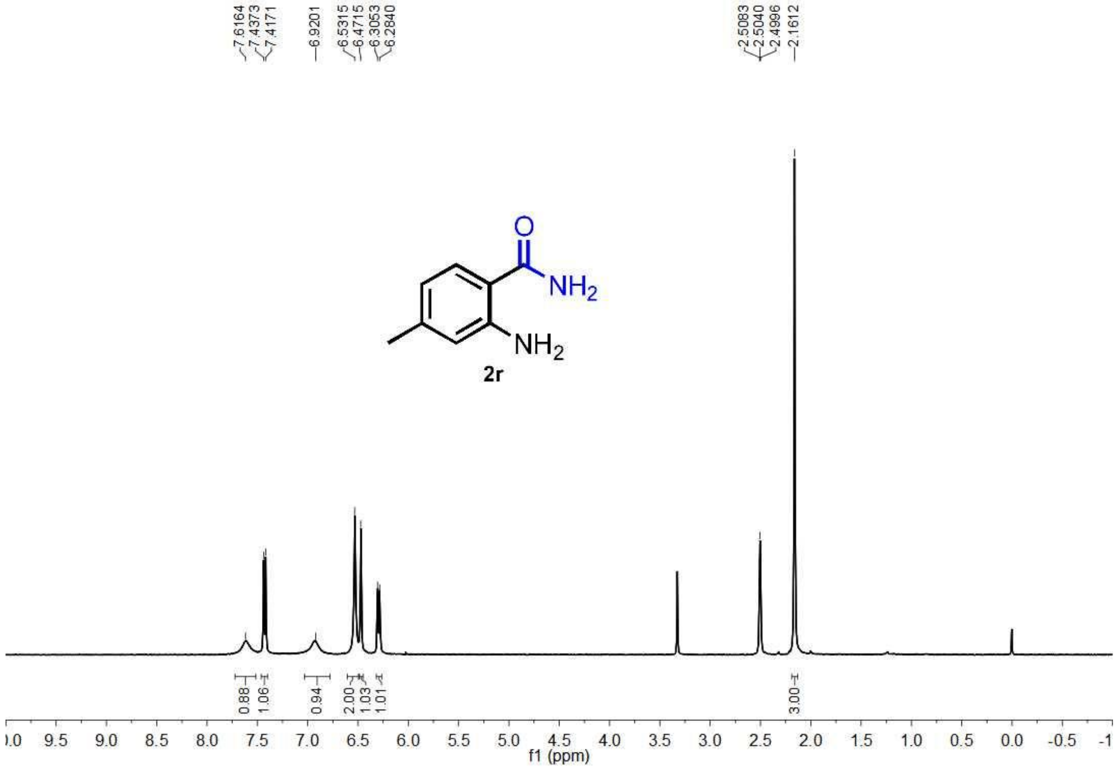

^{13}C NMR of product 2r in *d6*-DMSO (100 MHz)

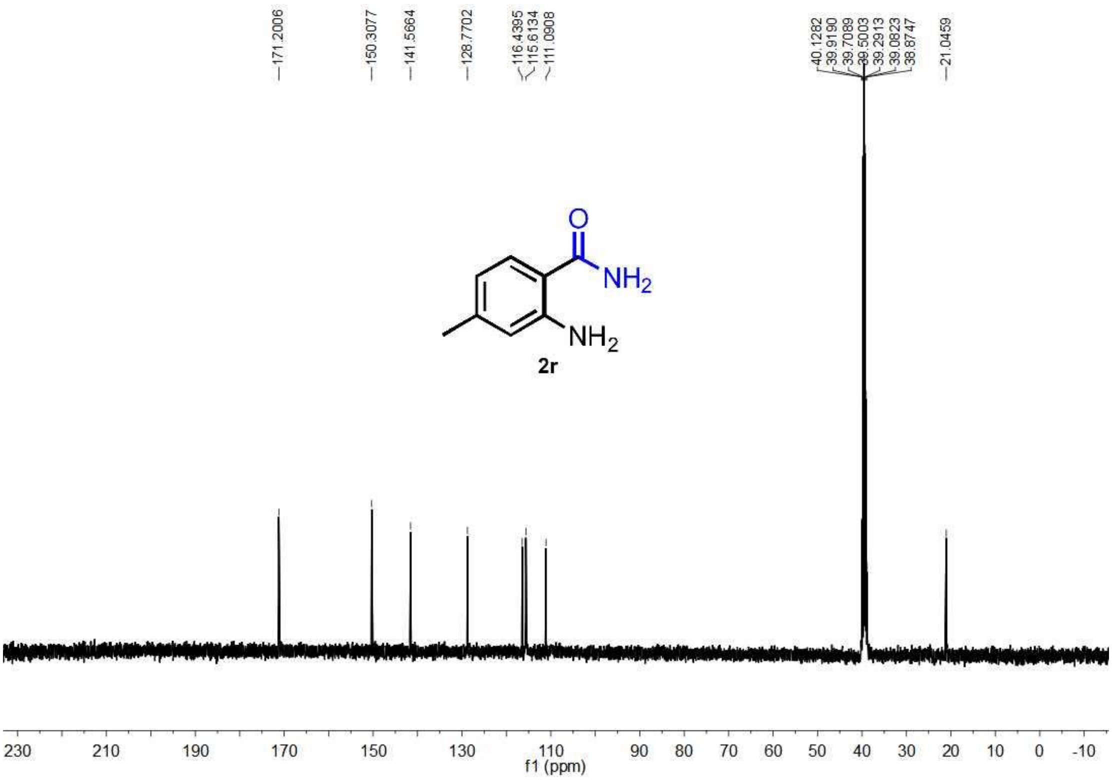

^{1}H NMR of product 2s in *d6*-DMSO (400 MHz)

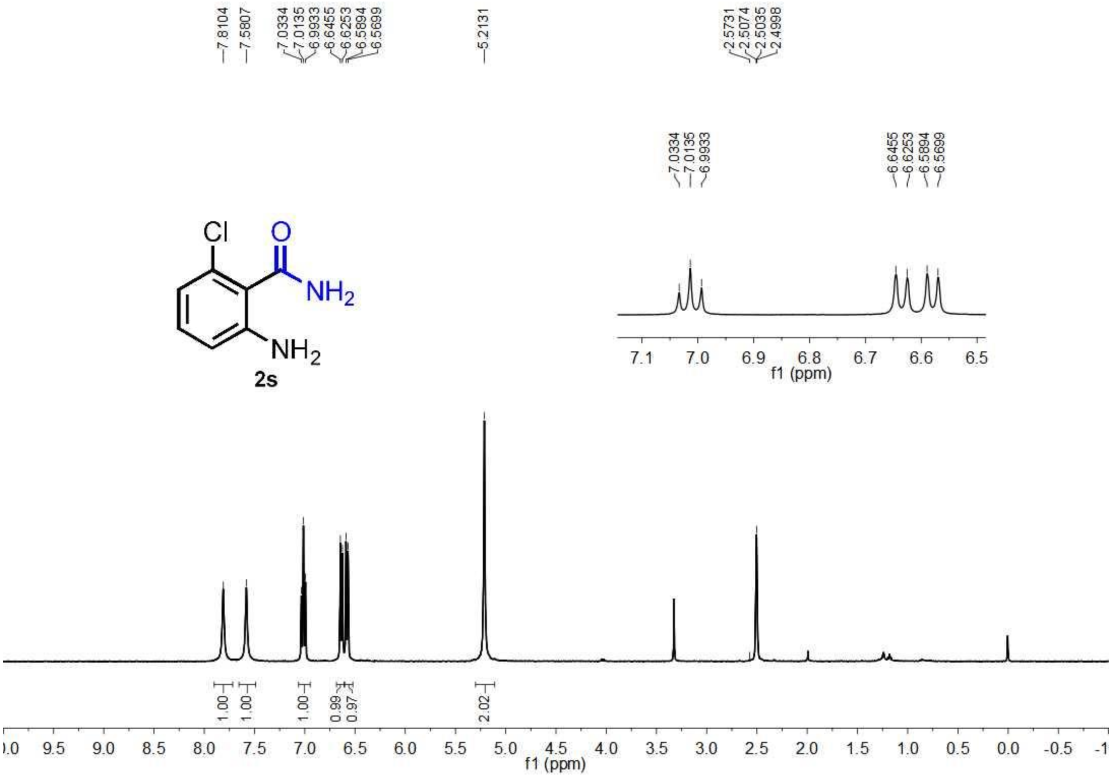

^{13}C NMR of product 2s in d_6-DMSO (100 MHz)

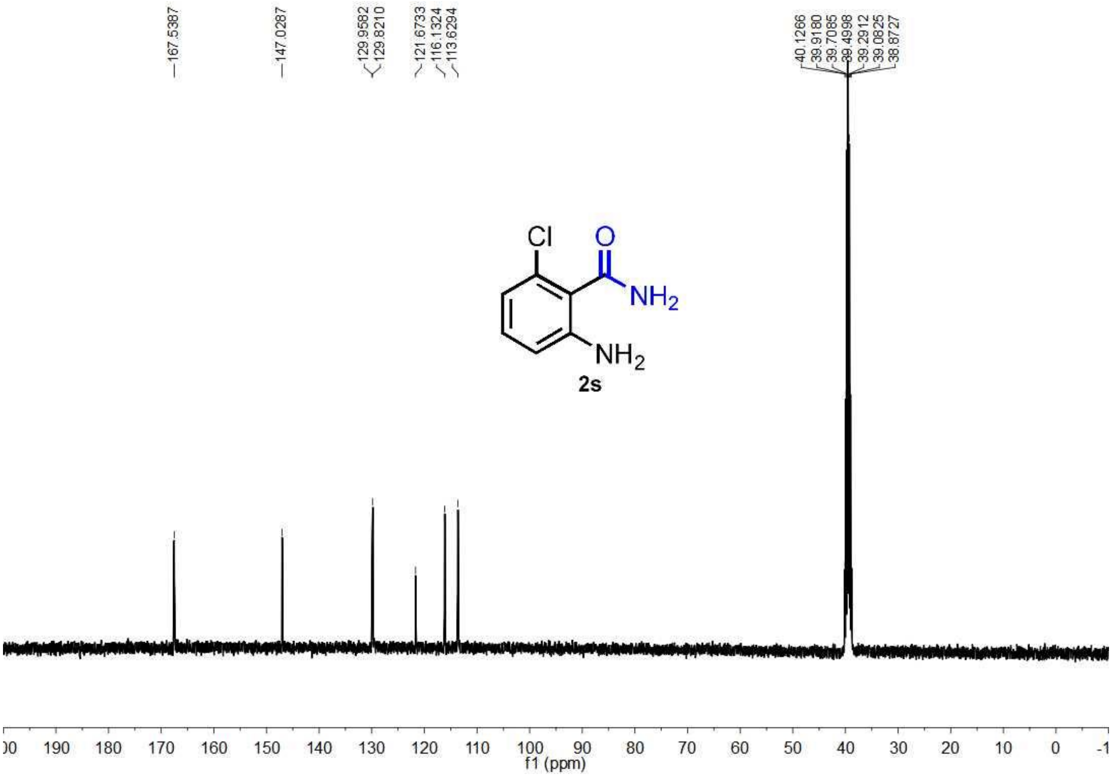

^{1}H NMR of product 2t in d_6-DMSO (400 MHz)

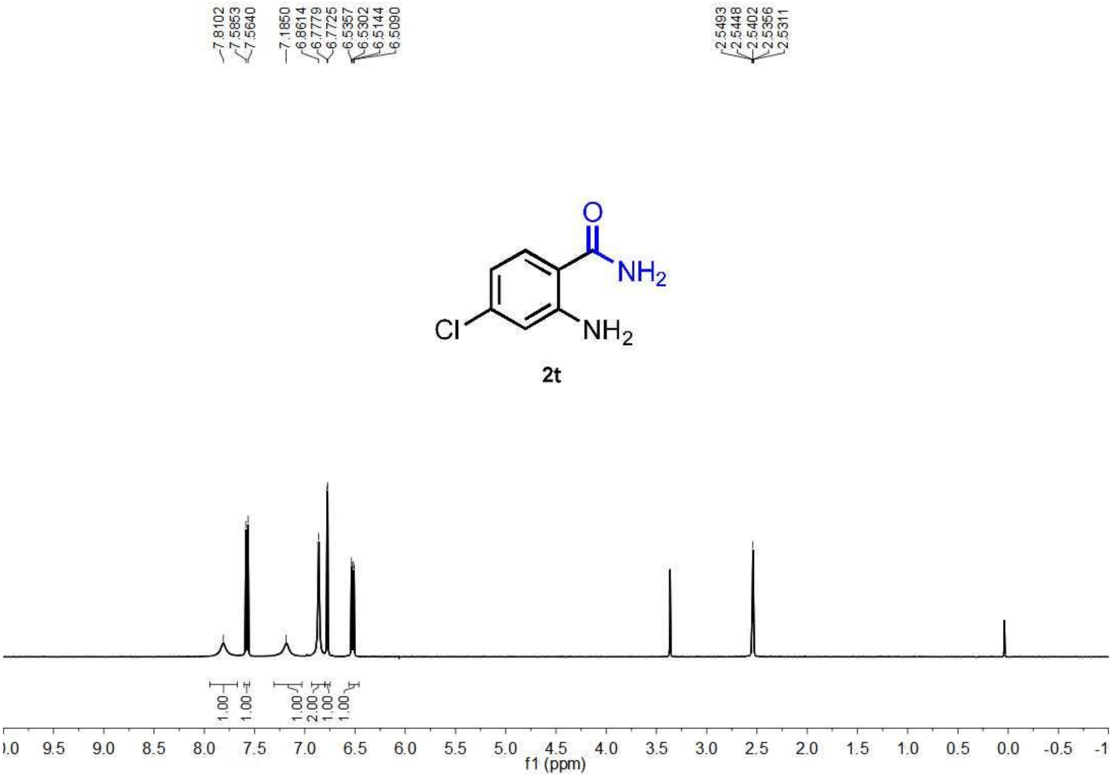

^{13}C NMR of product 2t in d_6-DMSO (100 MHz)

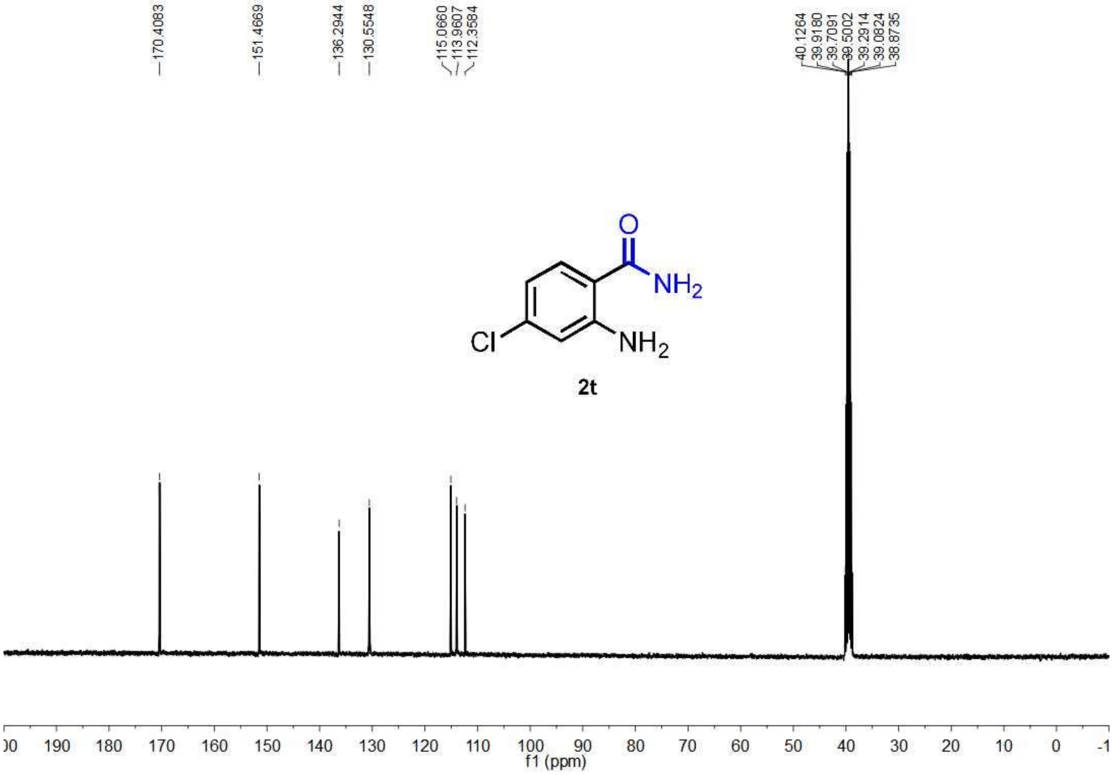

^{1}H NMR of product 2u in d_6-DMSO (400 MHz)

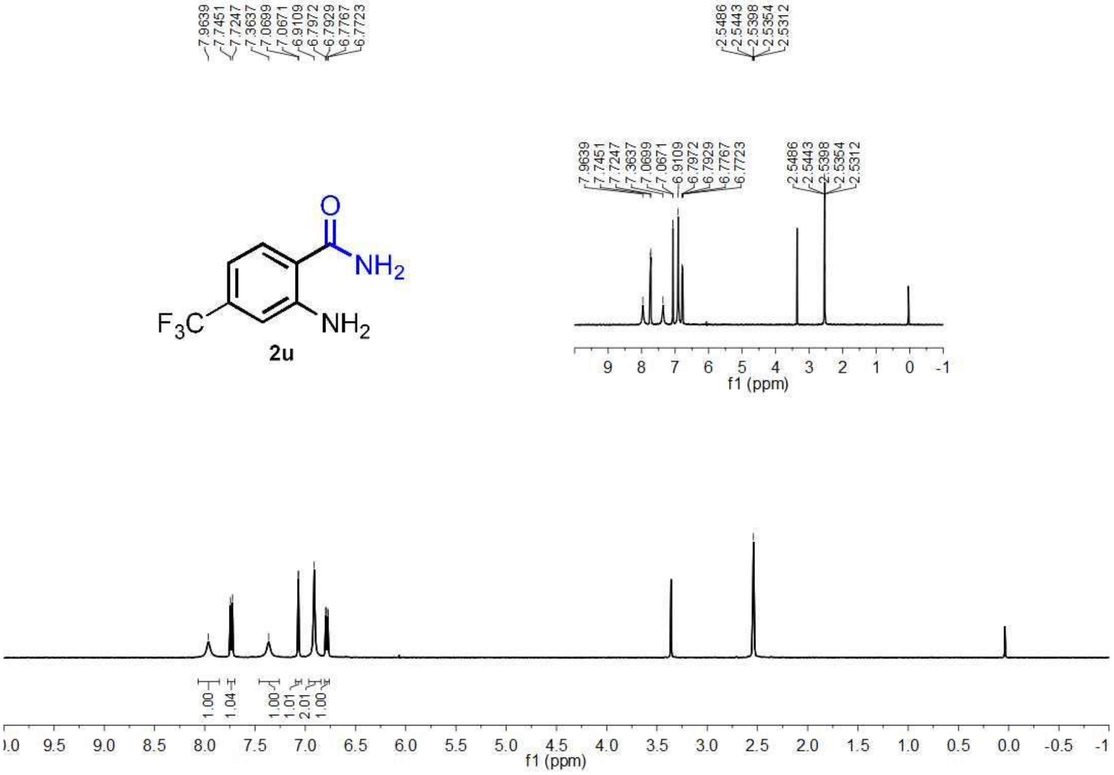

^{13}C NMR of product 2u in d_6-DMSO (100 MHz)

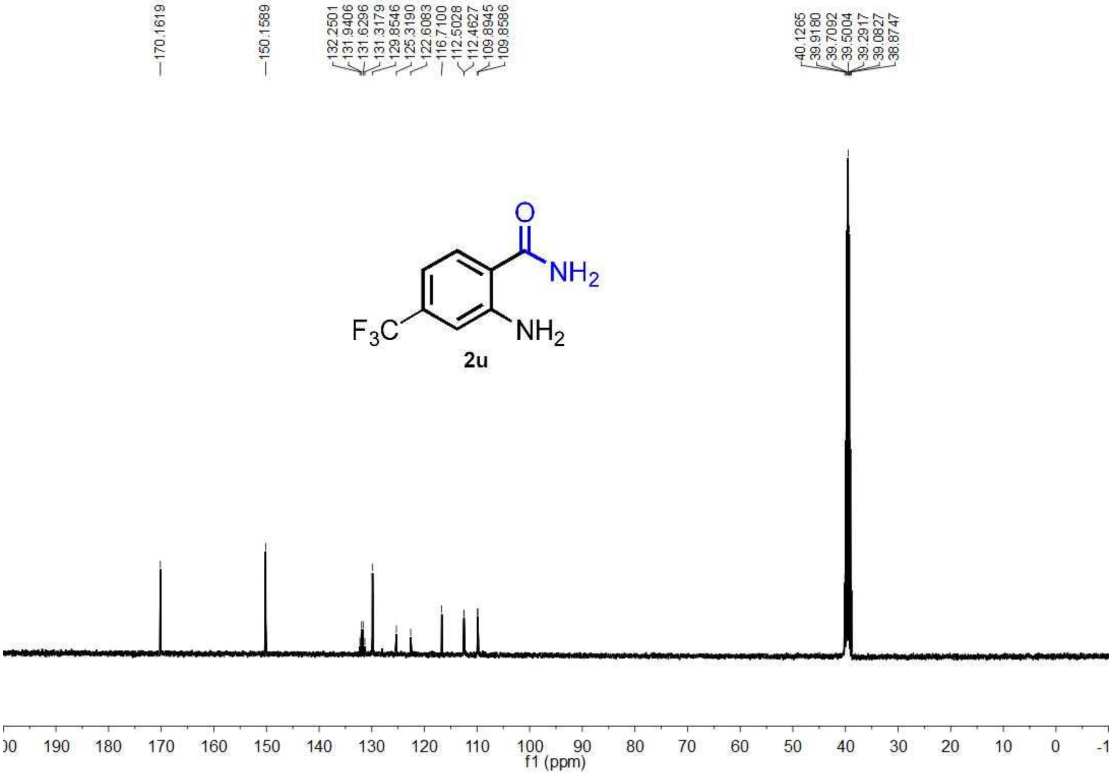

^{1}H NMR of product 2v in d_6-DMSO (400 MHz)

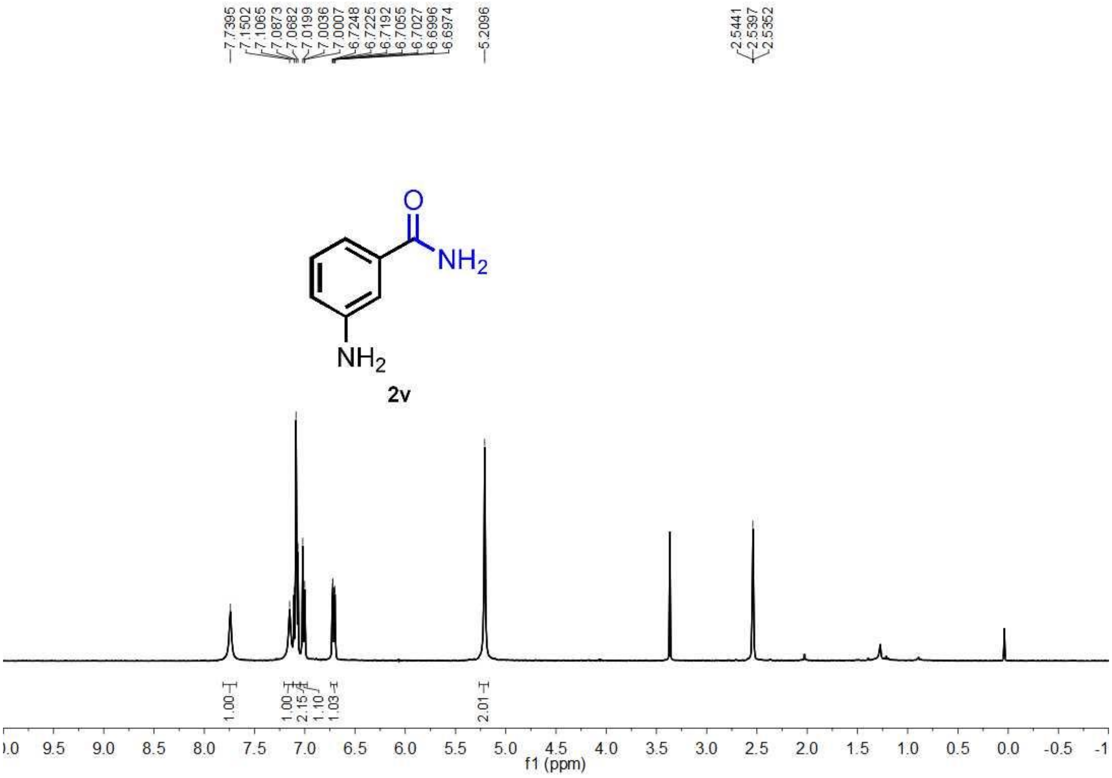

^{13}C NMR of product 2v in d_6-DMSO (100 MHz)

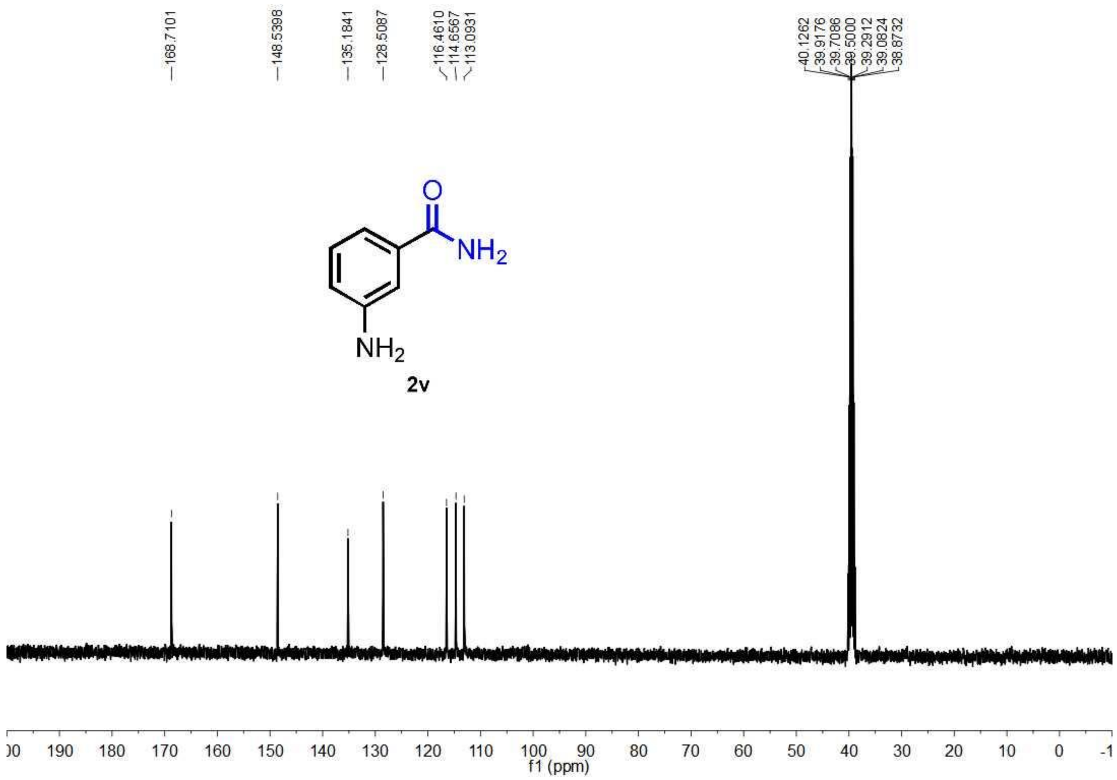

^{1}H NMR of product 2w in d_6-DMSO (400 MHz)

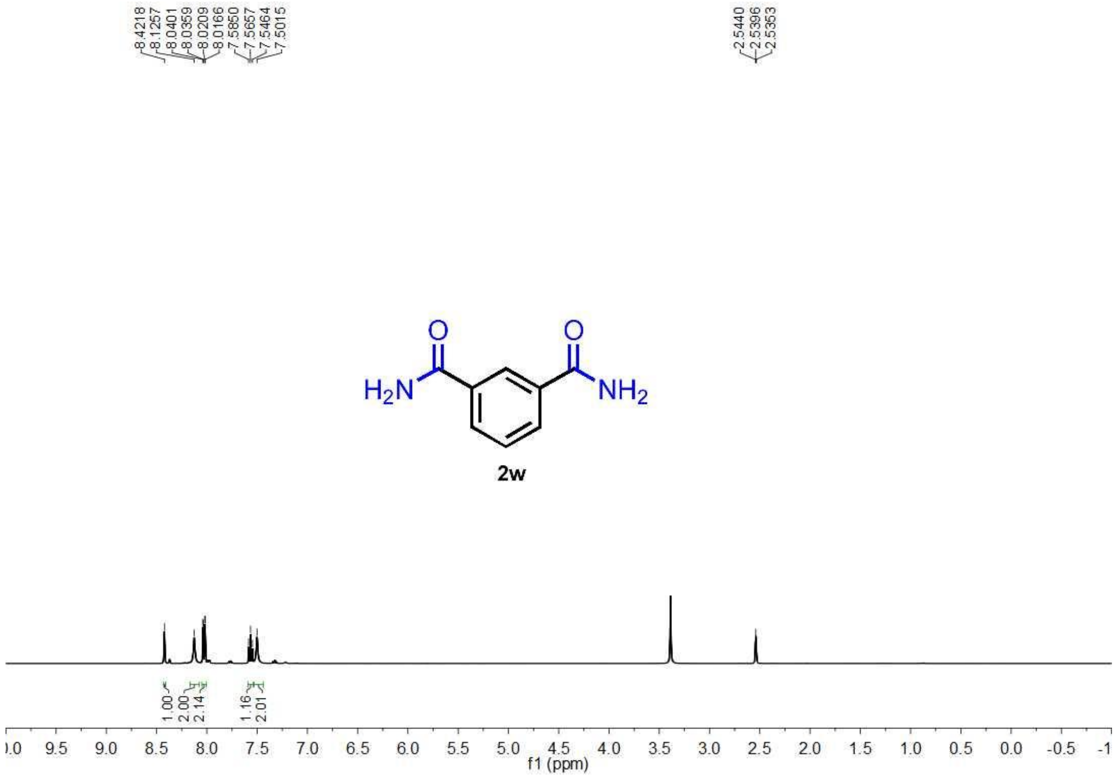

^{13}C NMR of product 2w in *d_6*-DMSO (100 MHz)

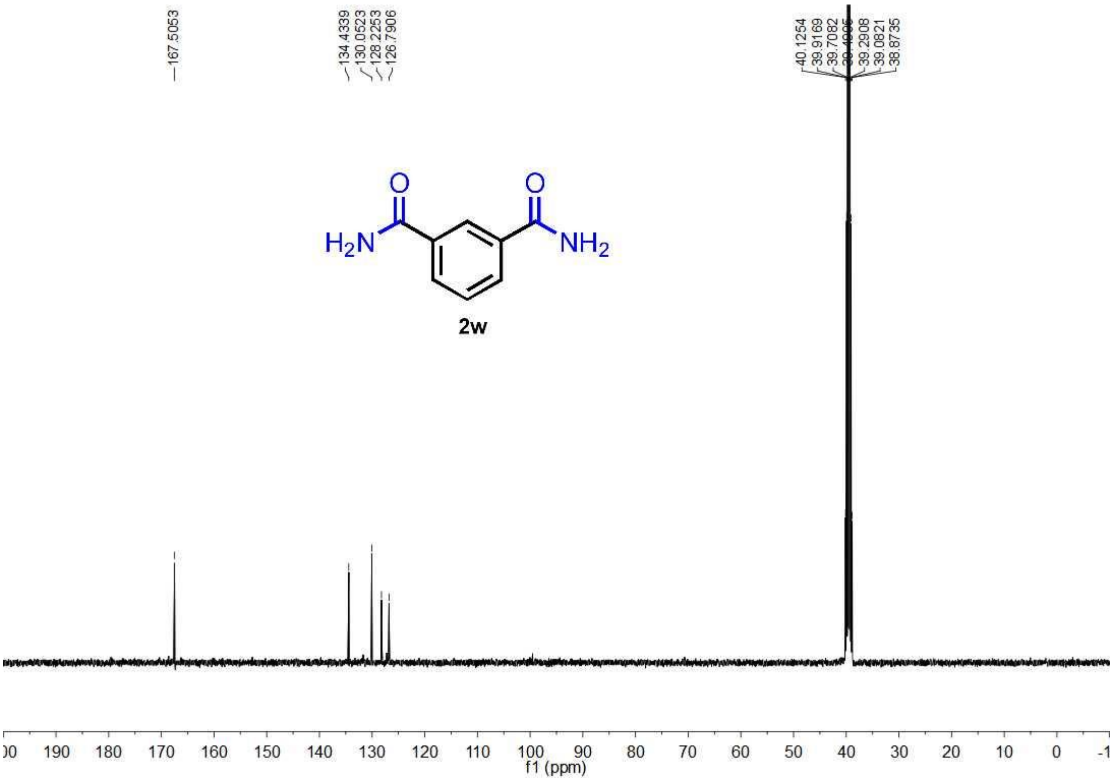

^{1}H NMR of product 2x in *d_6*-DMSO (400 MHz)

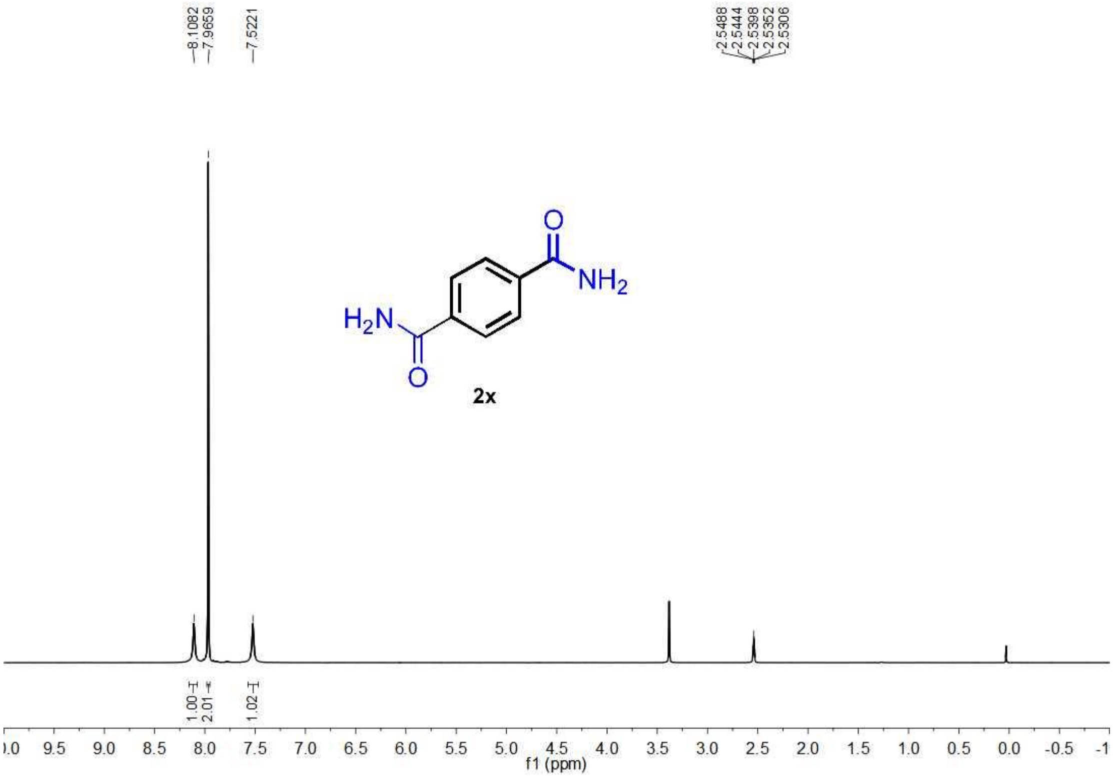

^{13}C NMR of product 2x in *d_6*-DMSO (100 MHz)

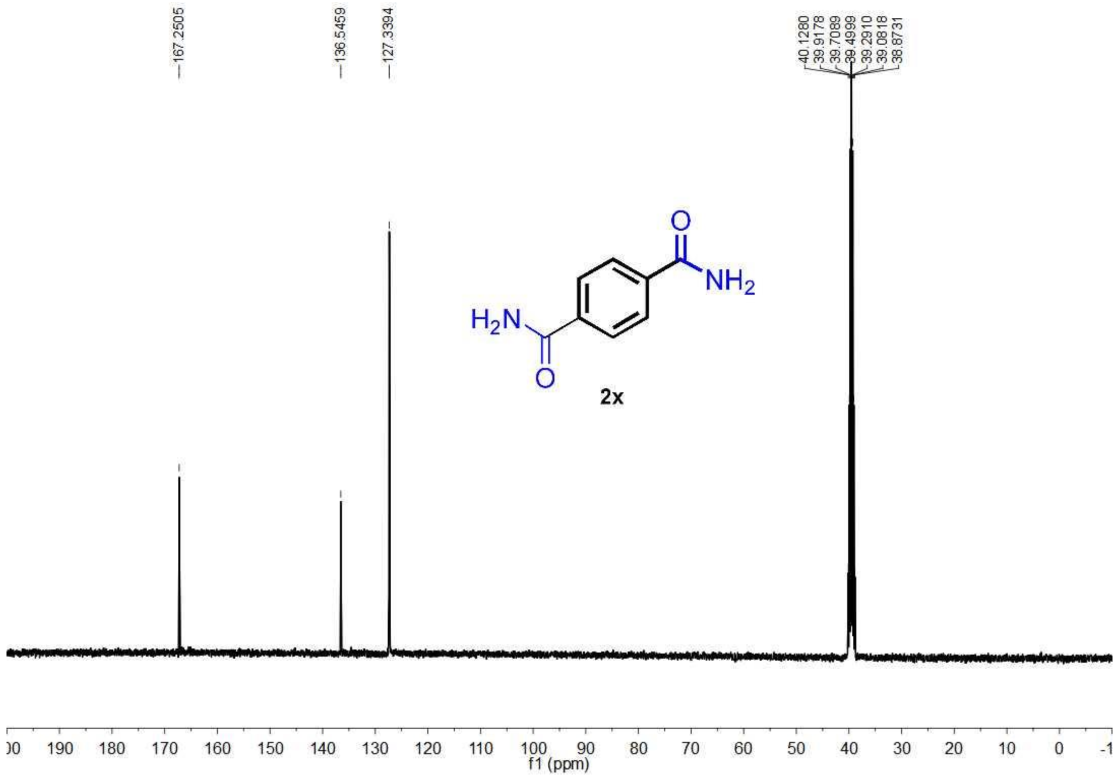

^{1}H NMR of product 2y in *d_6*-DMSO (400 MHz)

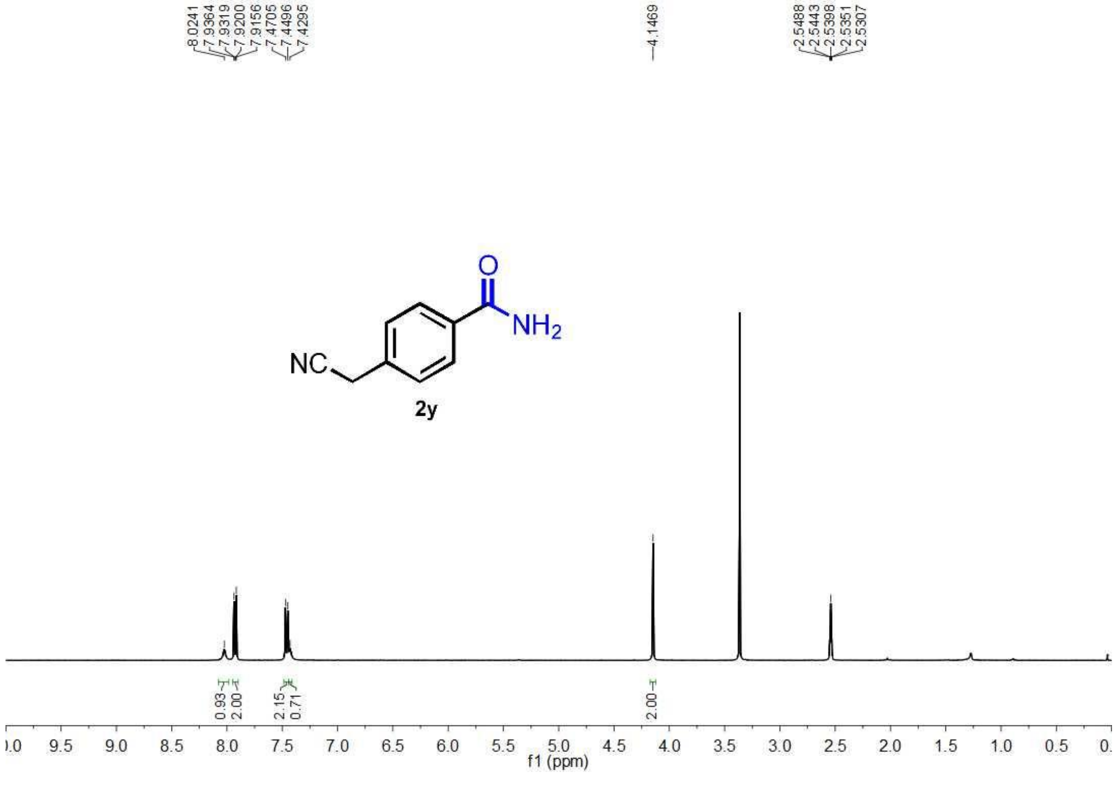

^{13}C NMR of product 2y in *d$_6$*-DMSO (100 MHz)

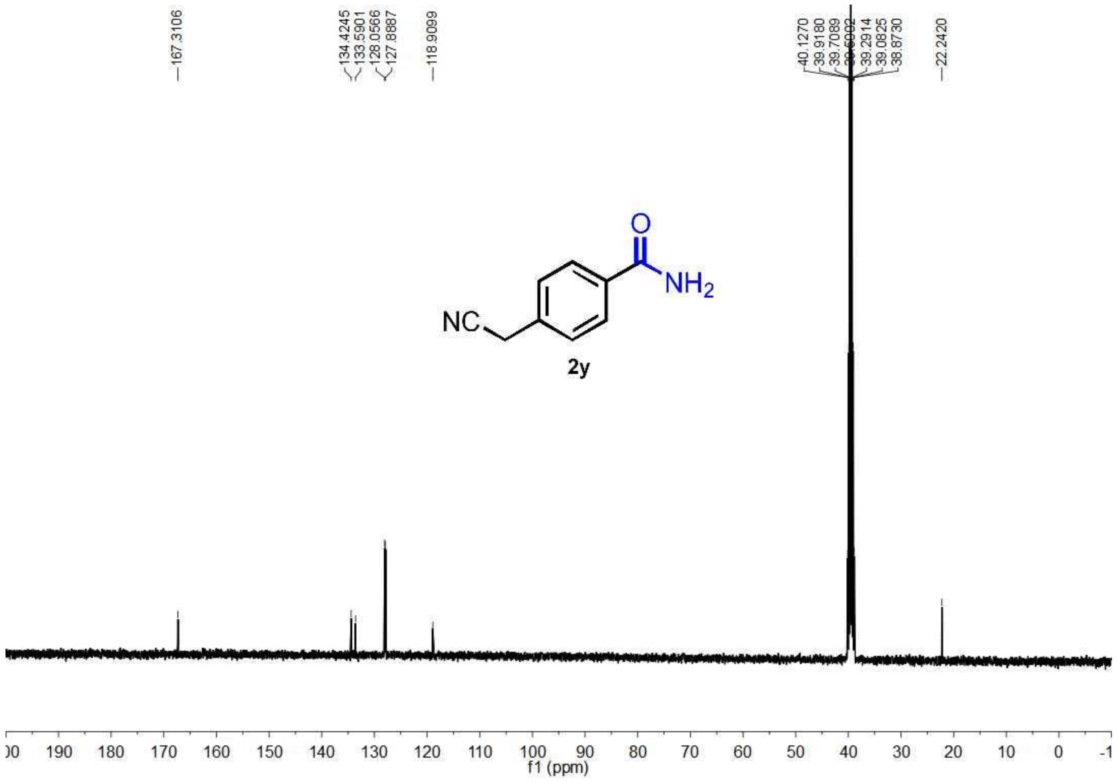

^{1}H NMR of product 2z in *d$_6$*-DMSO (400 MHz)

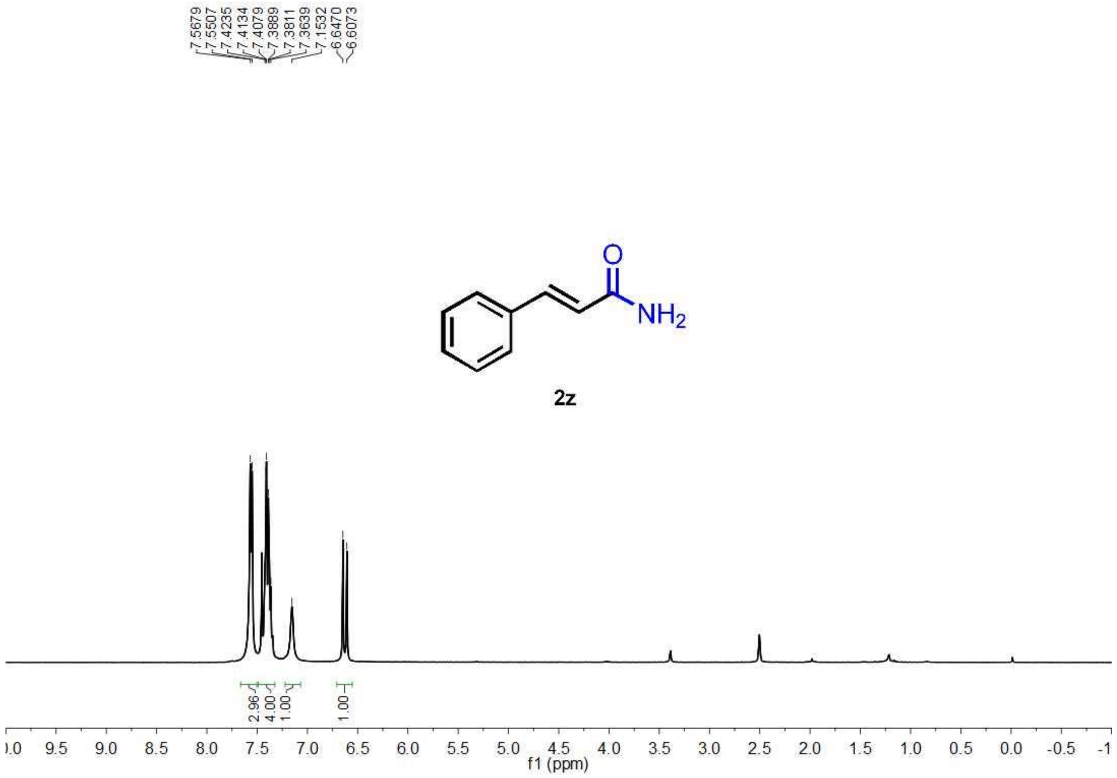

^{13}C NMR of product 2z in *d_6*-DMSO (100 MHz)

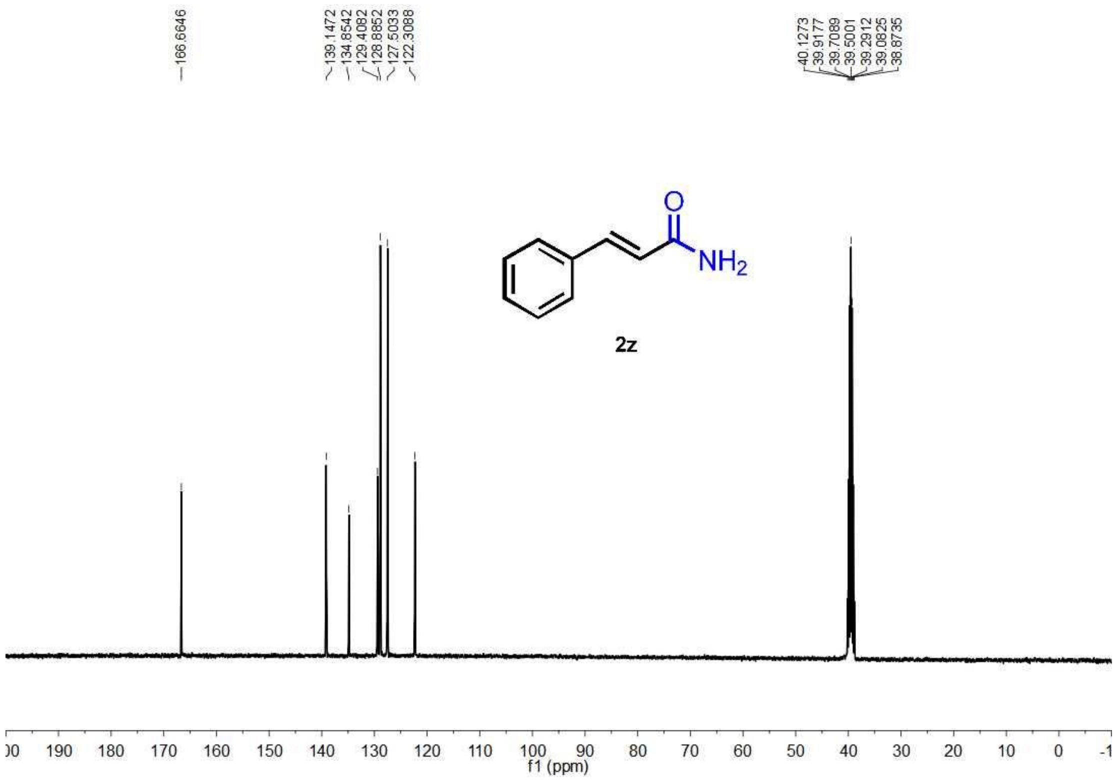

^{1}H NMR of product 2aa in *d_6*-DMSO (400 MHz)

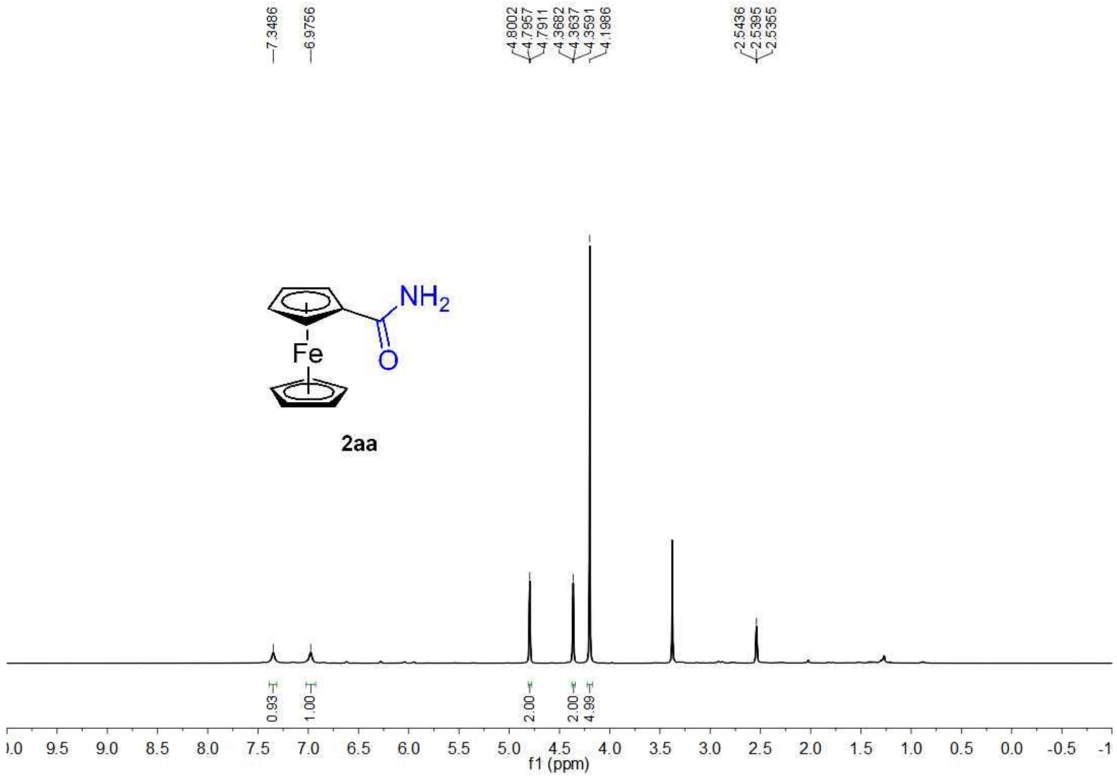

^{13}C NMR of product 2aa in d_6-DMSO (100 MHz)

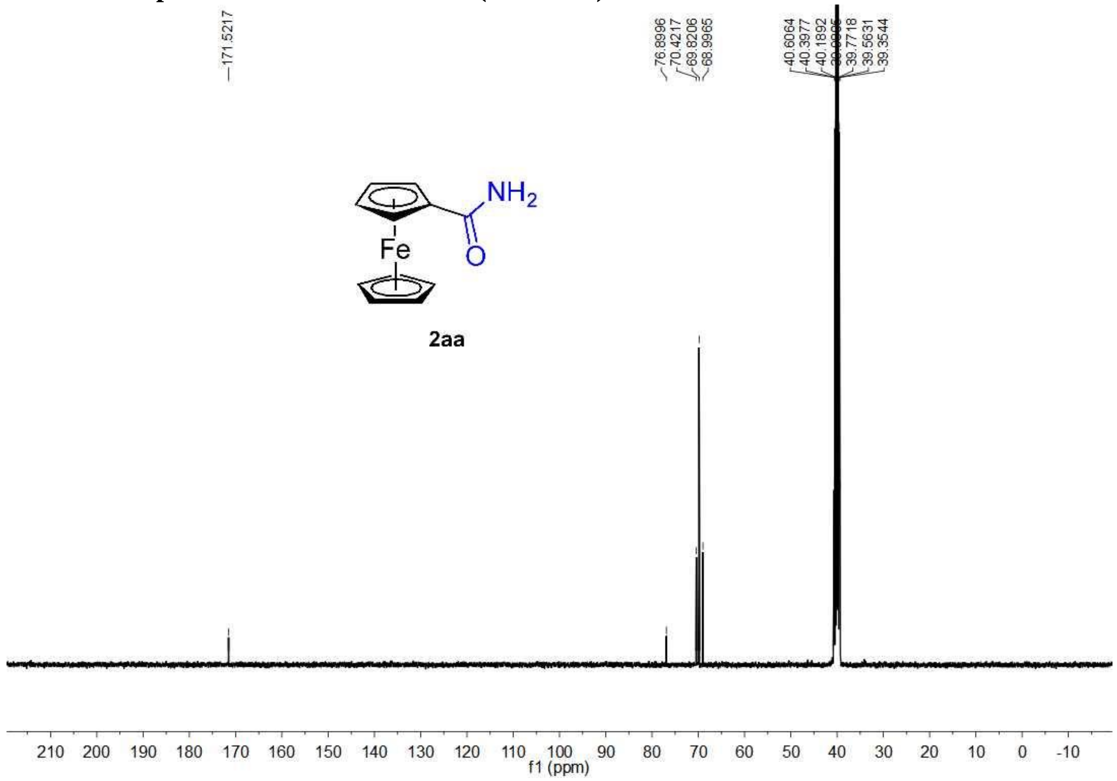

^{1}H NMR of product 2a' in d_6-DMSO (400 MHz)

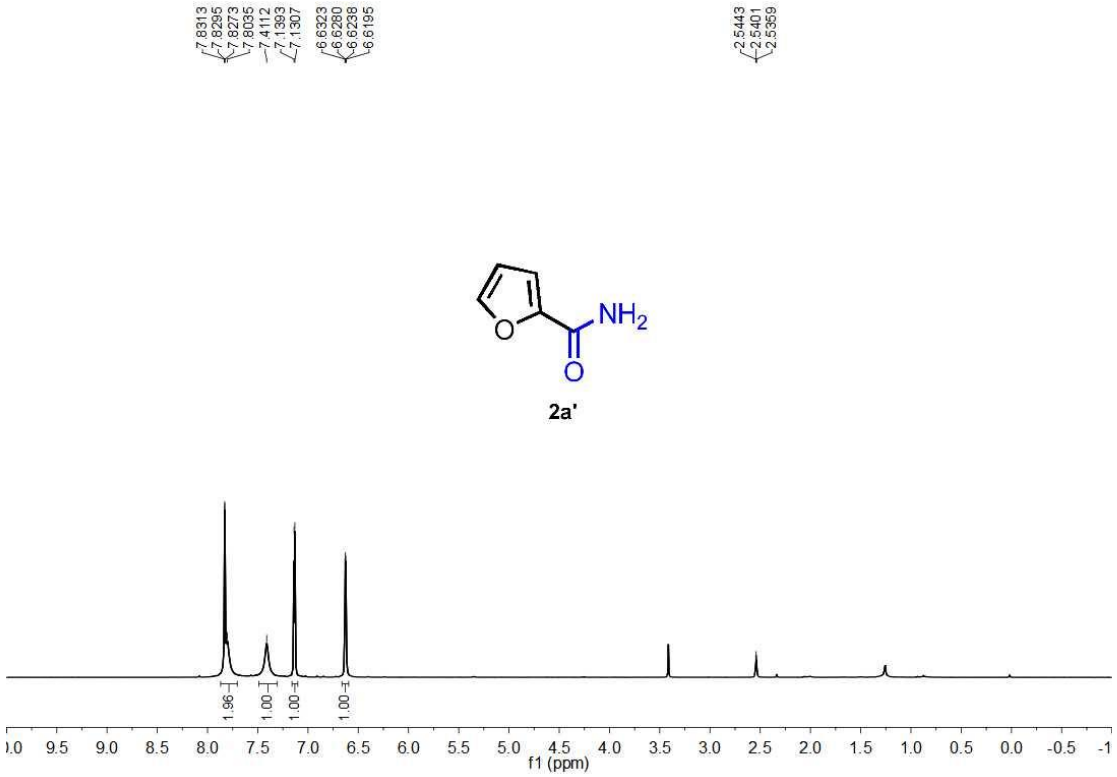

^{13}C NMR of product 2a' in d_6-DMSO (100 MHz)

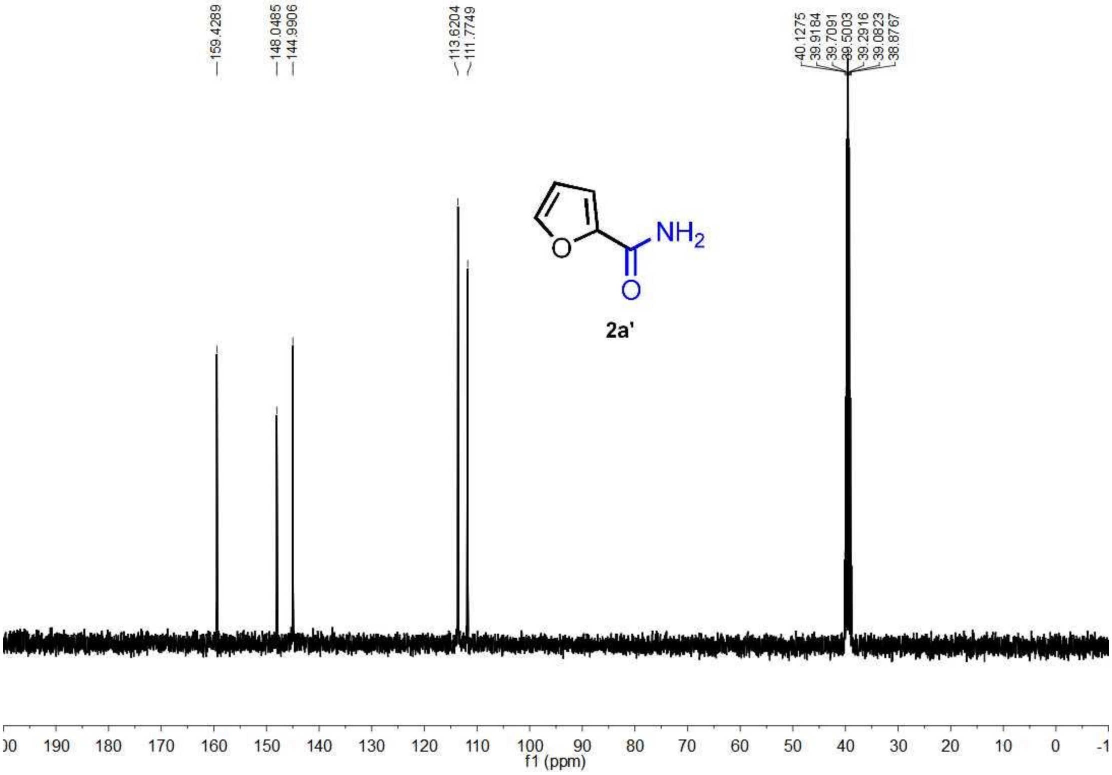

^{1}H NMR of product 2b' in d_6-DMSO (400 MHz)

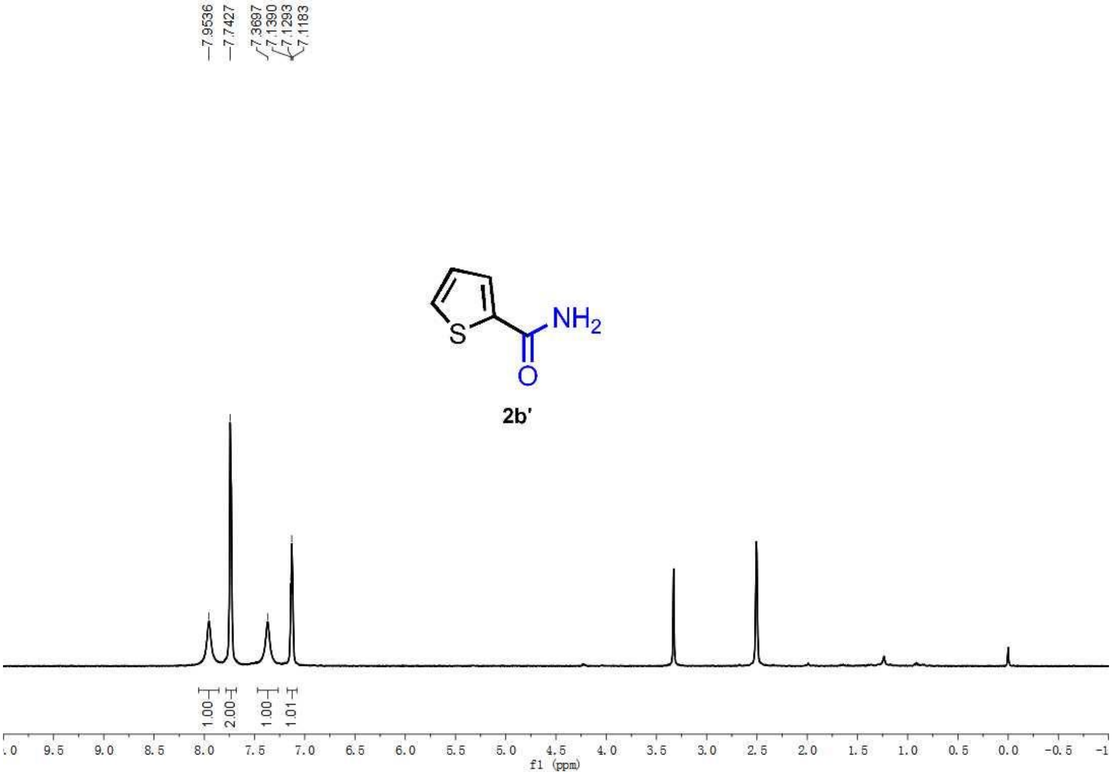

^{13}C NMR of product 2b' in d_6-DMSO (100 MHz)

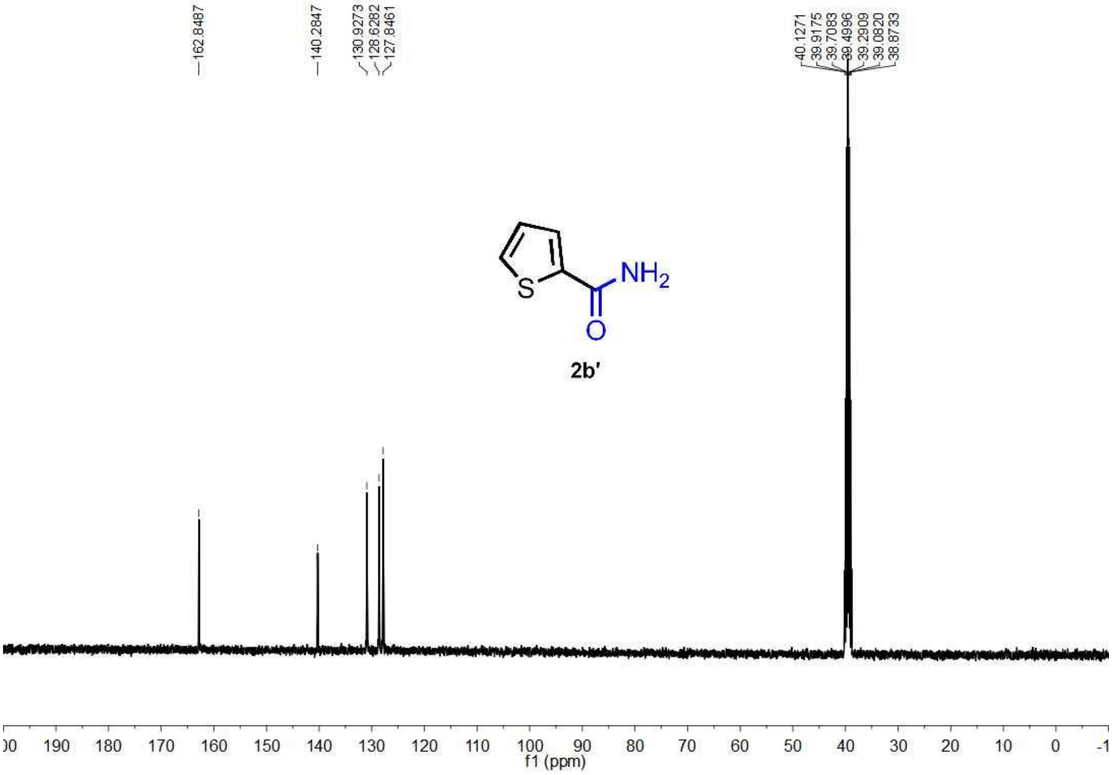

^{1}H NMR of product 2c' in d_6-DMSO (400 MHz)

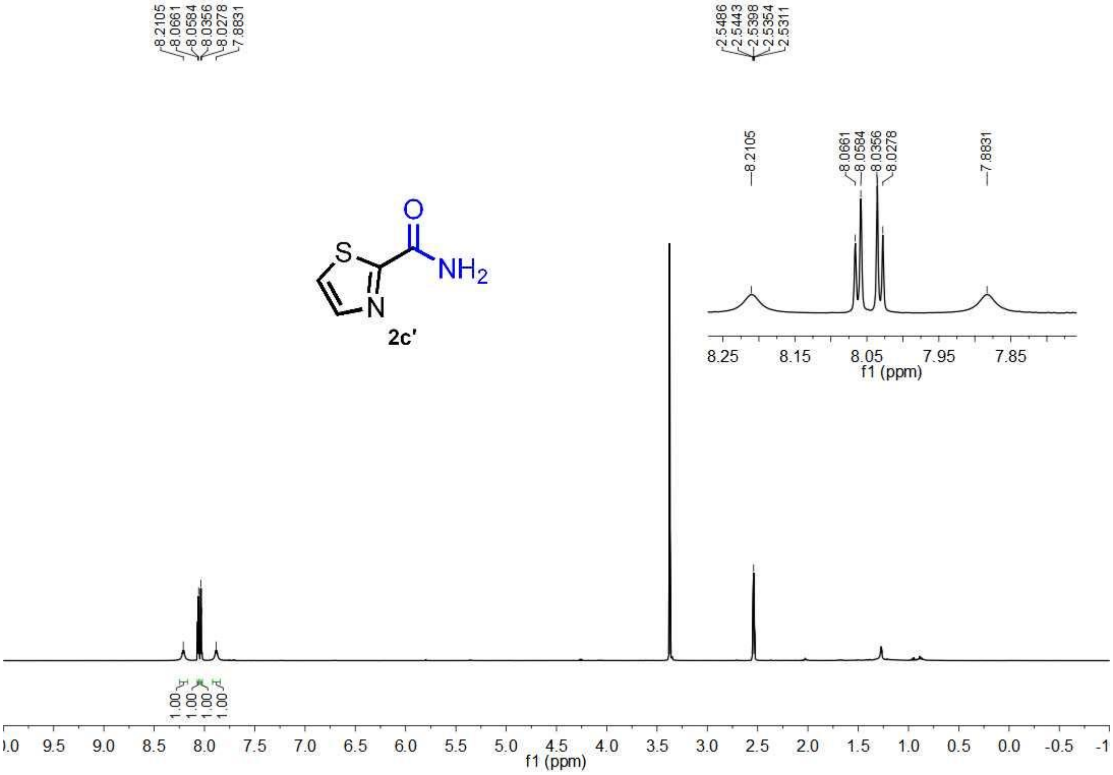

^{13}C NMR of product 2c' in *d_6*-DMSO (100 MHz)

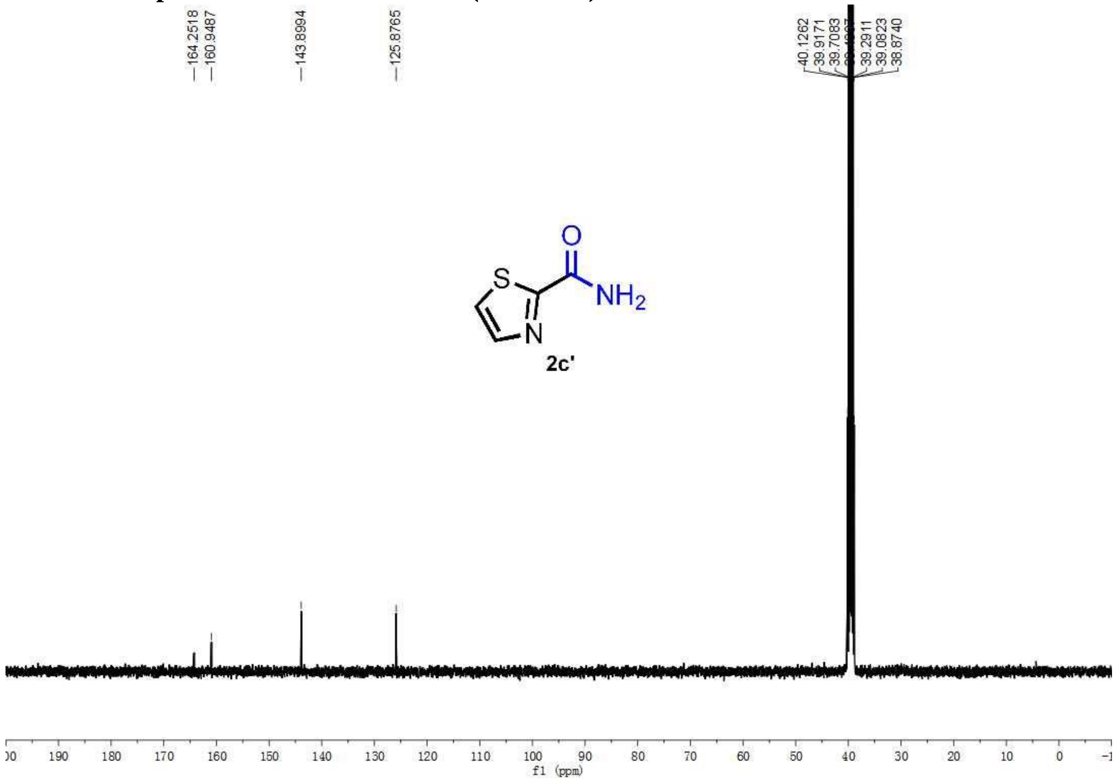

^{1}H NMR of product 2d' in $CDCl_3$ (400 MHz)

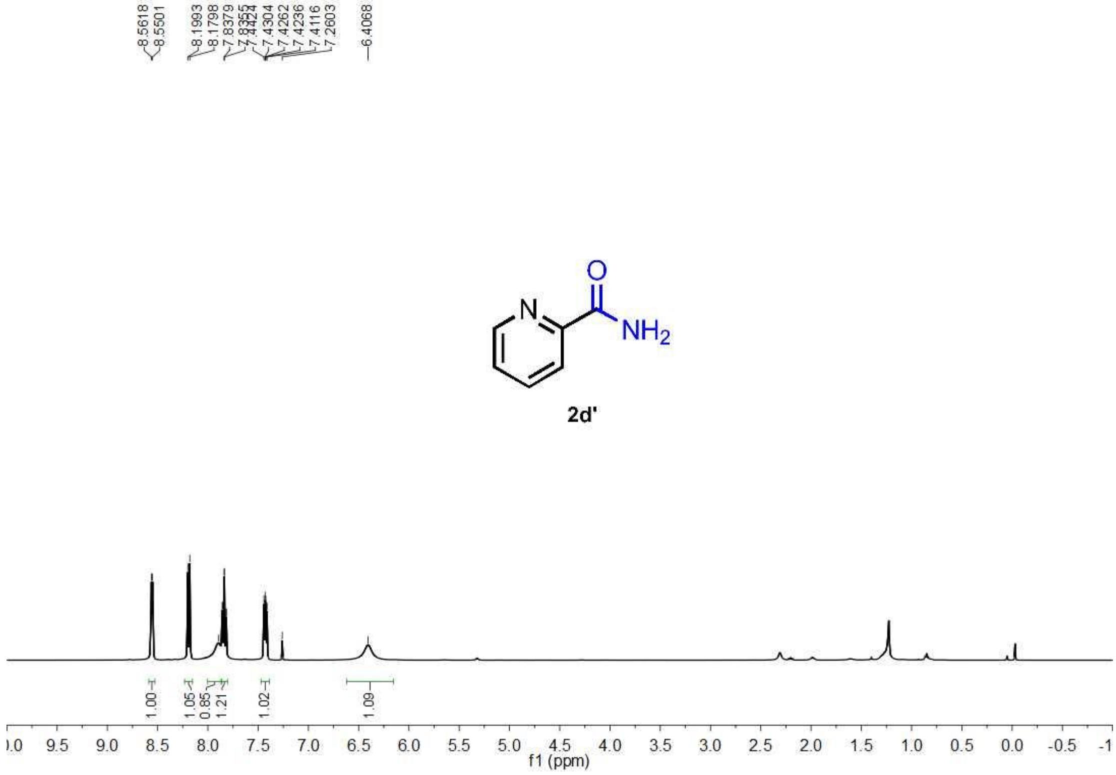

^{13}C NMR of product 2d' in $CDCl_3$ (100 MHz)

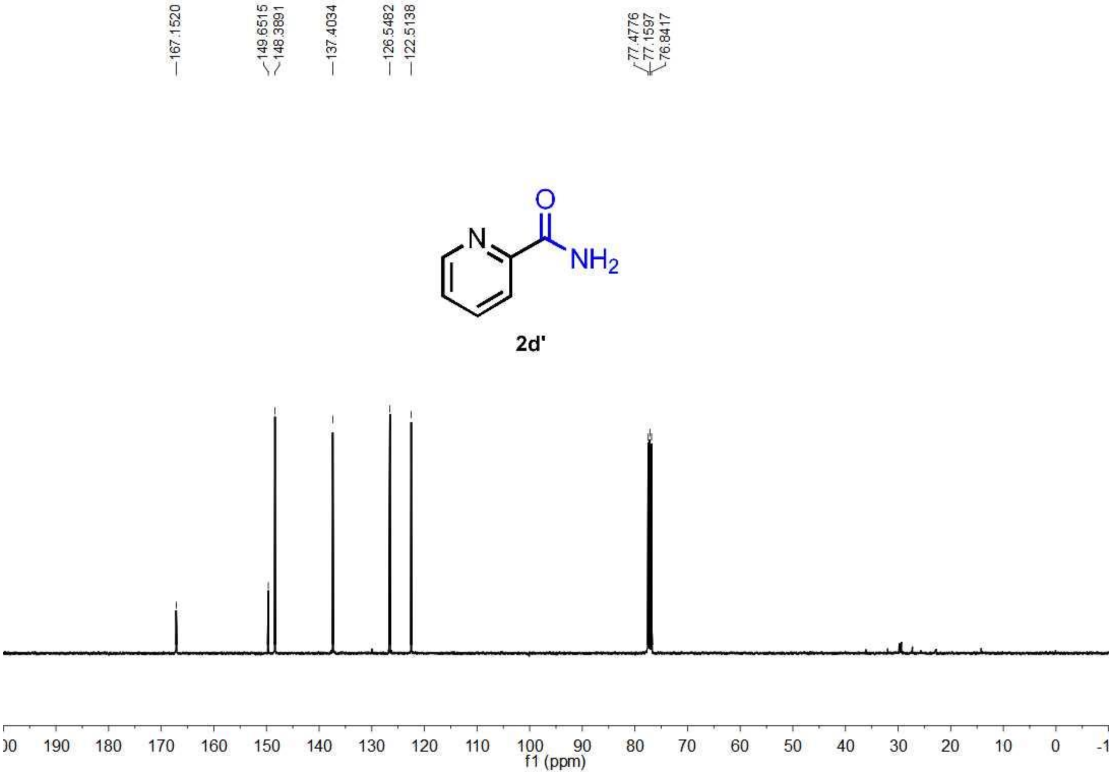

^{1}H NMR of product 2e' in d_6-DMSO (400 MHz)

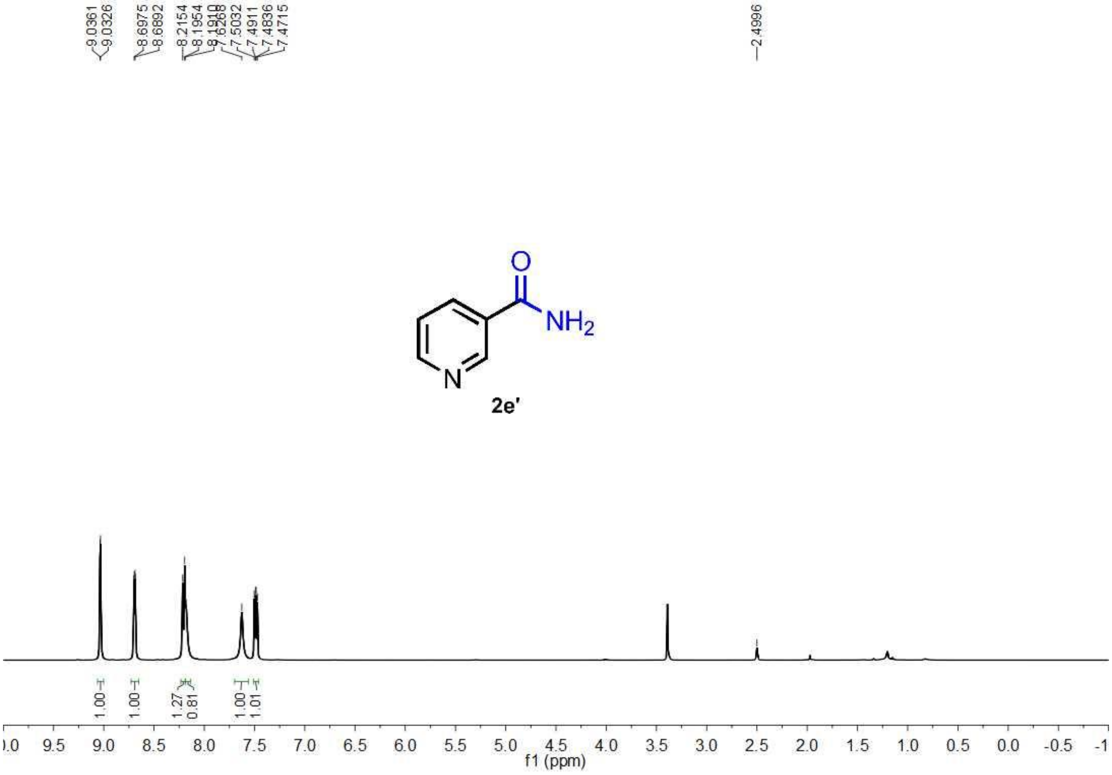

^{13}C NMR of product 2e' in *d*$_6$-DMSO (100 MHz)

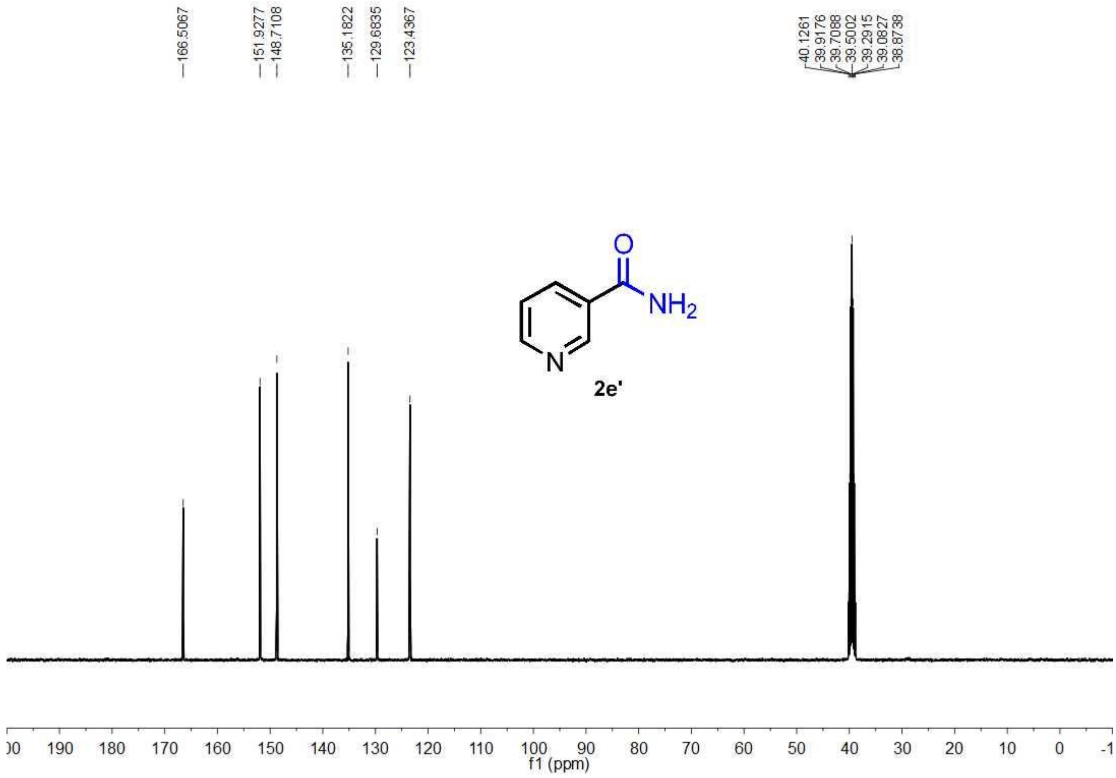

^{1}H NMR of product 2f' in *d*$_6$-DMSO (400 MHz)

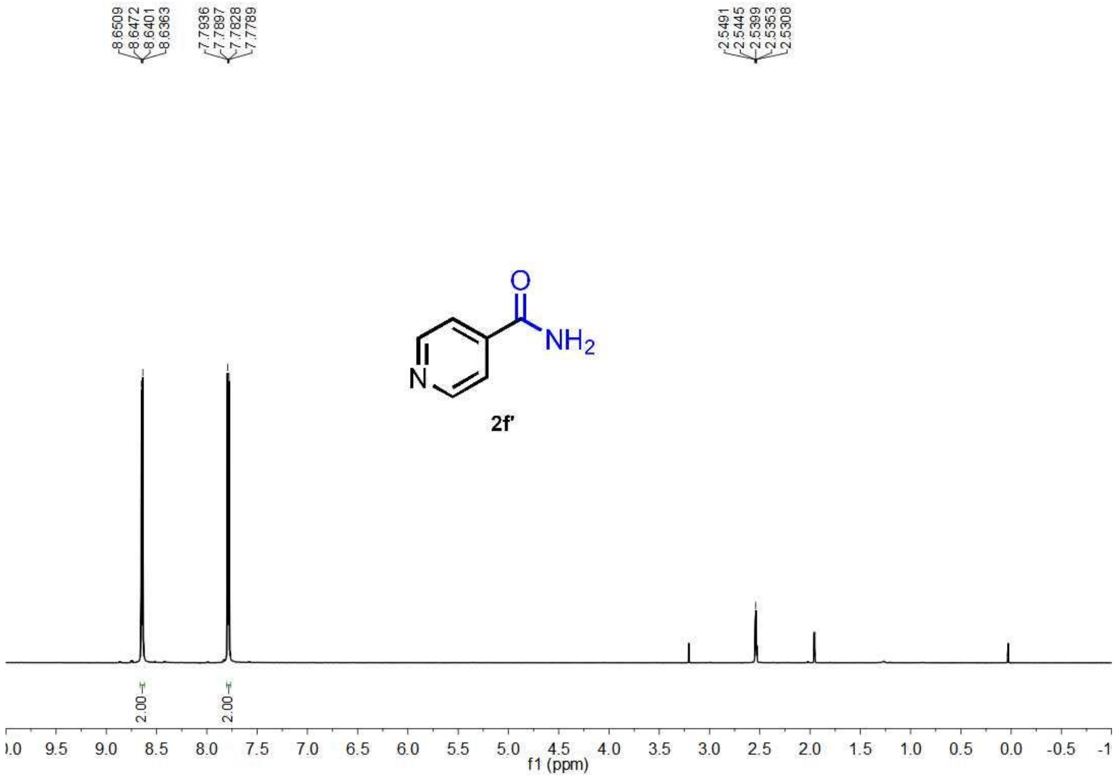

^{13}C NMR of product 2f' in *d_6*-DMSO (100 MHz)

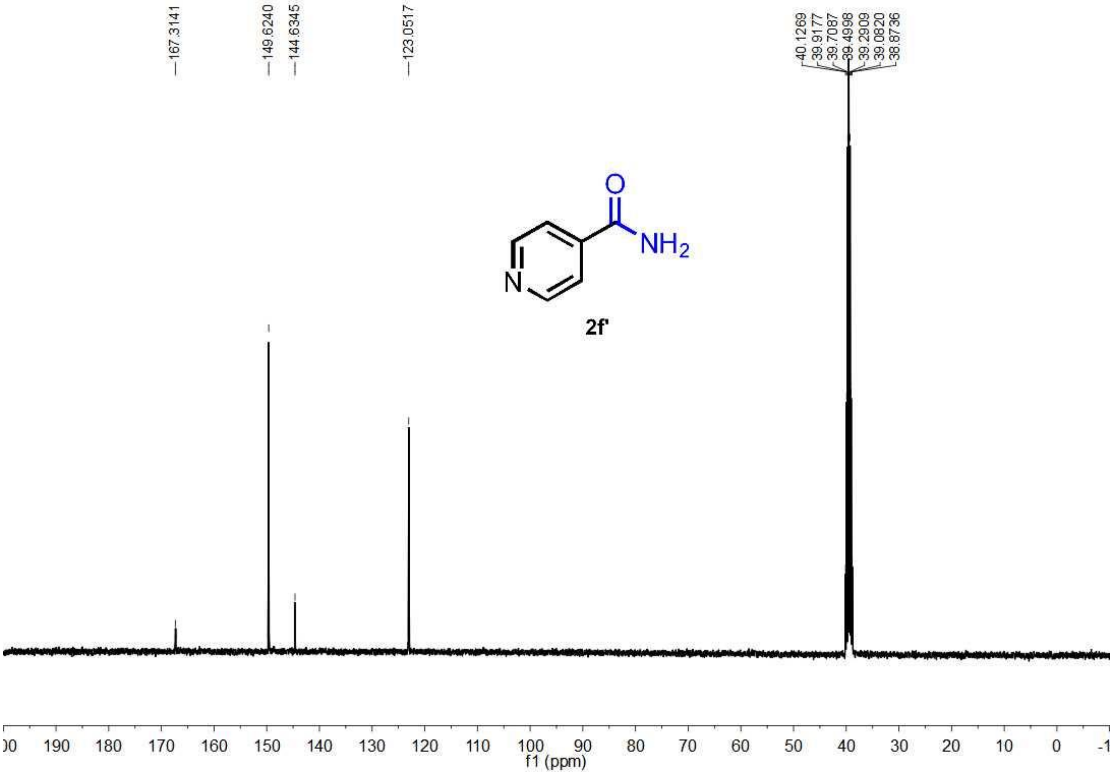

^{1}H NMR of product 2g' in *d_6*-DMSO (400 MHz)

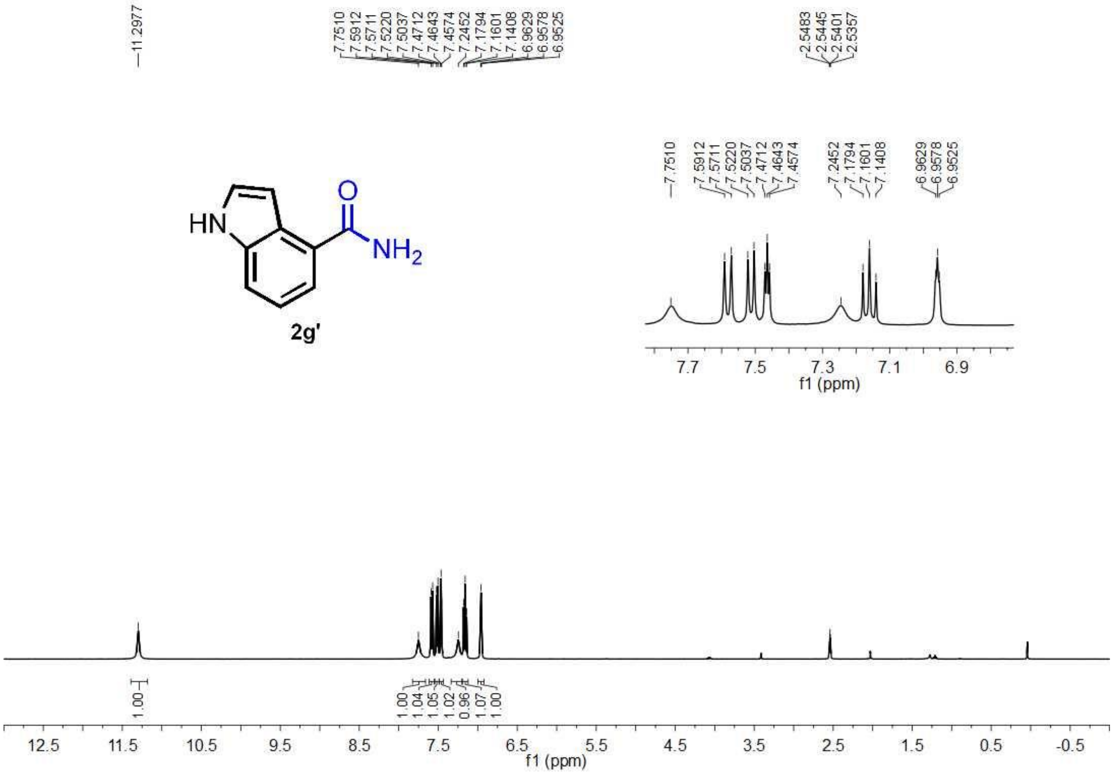

^{13}C NMR of product 2g' in d_6-DMSO (100 MHz)

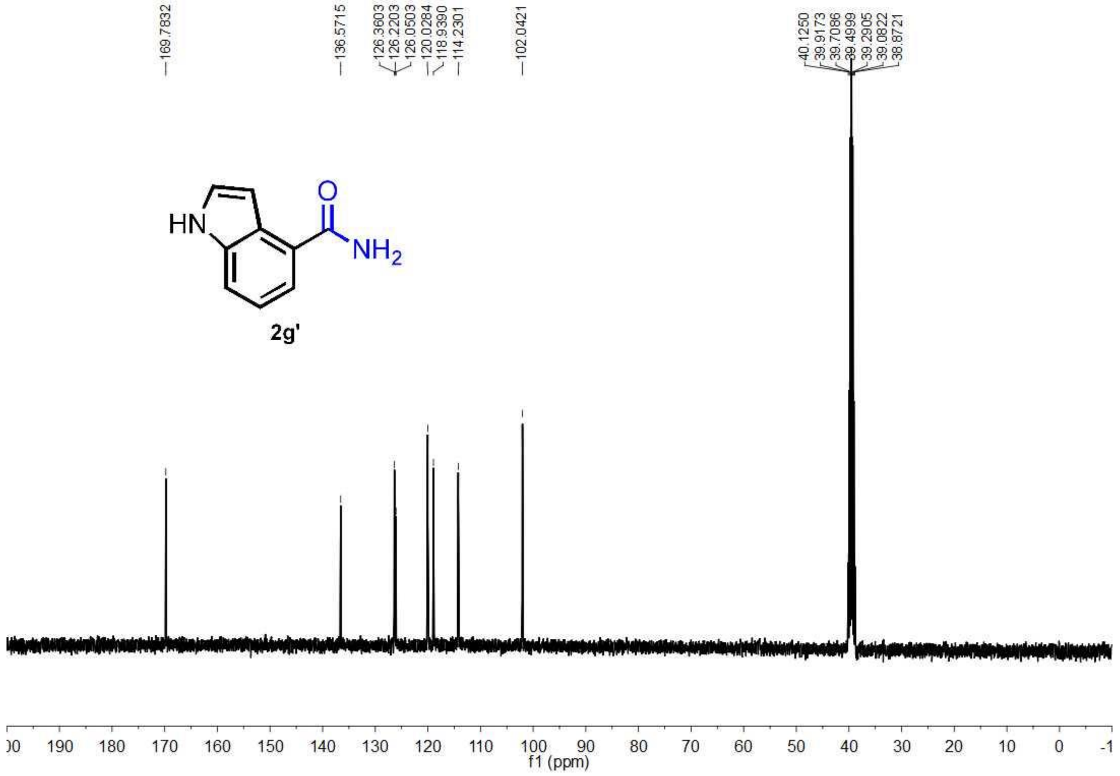

^{1}H NMR of product 2a'' in d_6-DMSO (400 MHz)

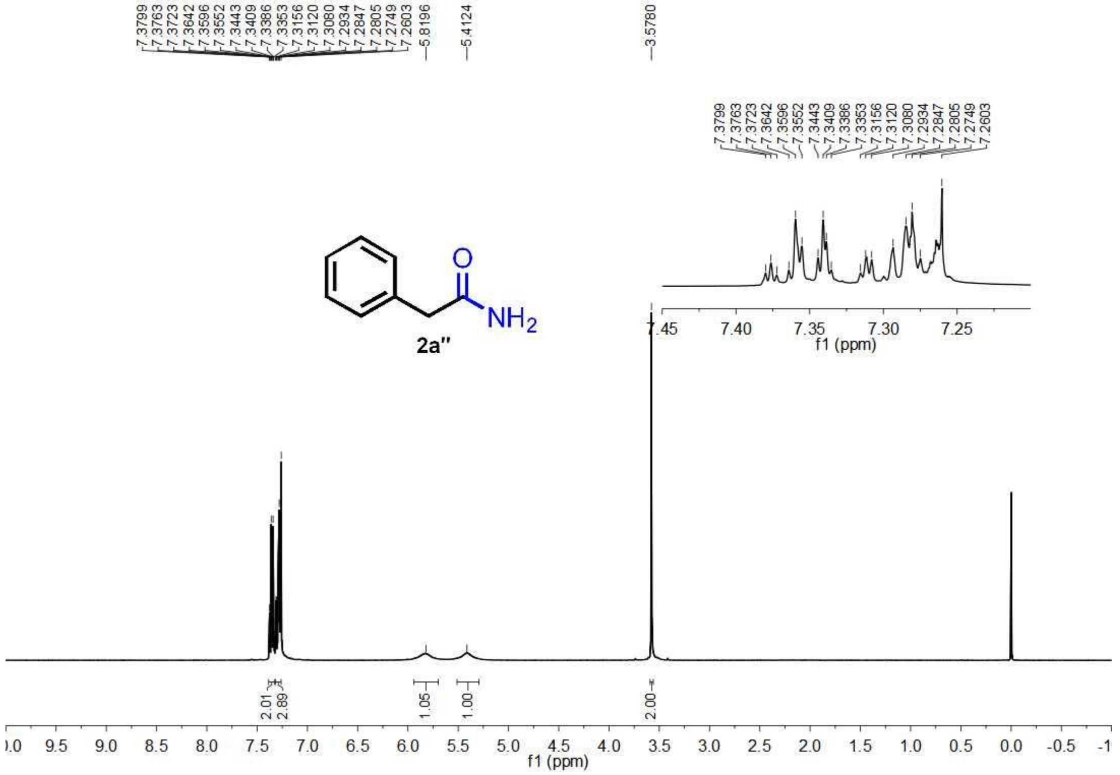

^{13}C NMR of product 2a'' in d_6-DMSO (100 MHz)

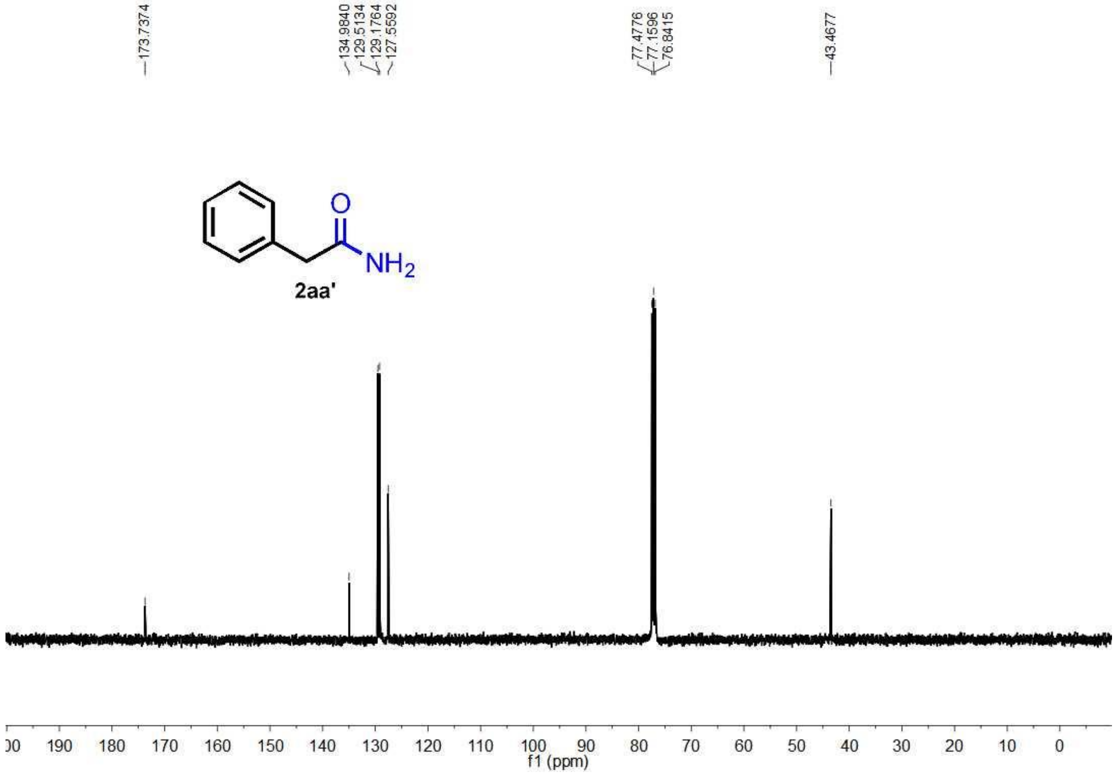

^{1}H NMR of product 2b'' in d_6-DMSO (400 MHz)

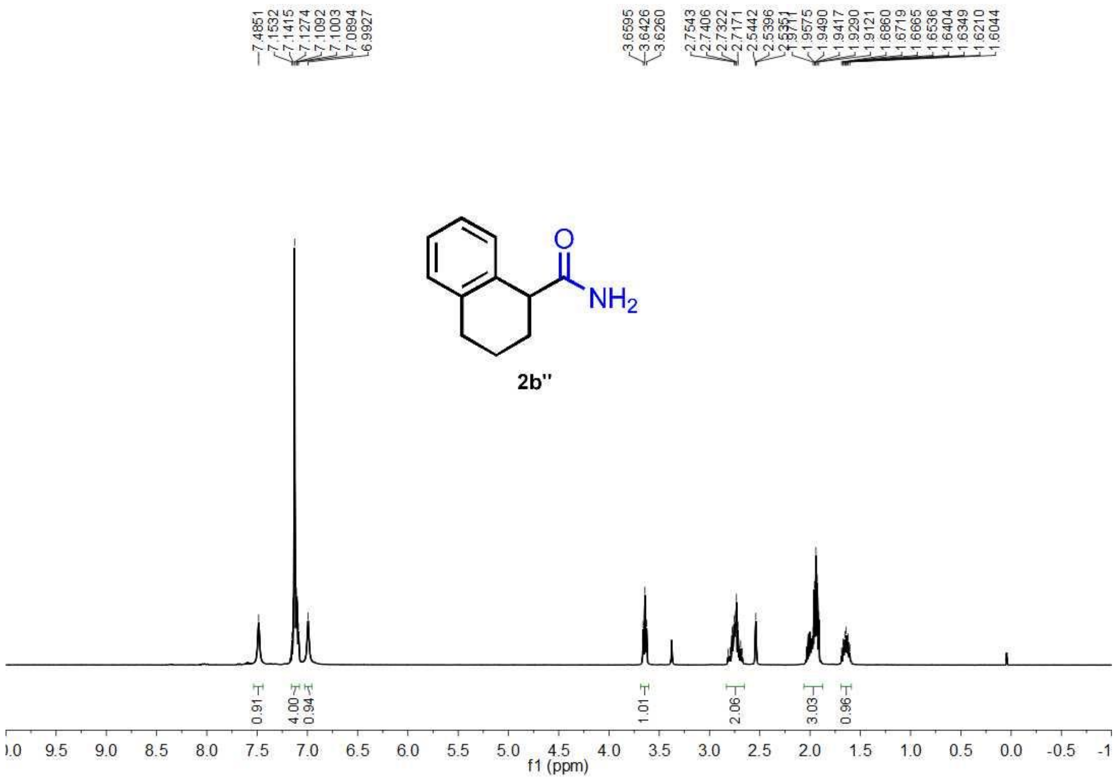

^{13}C NMR of product 2b'' in d_6-DMSO (100 MHz)

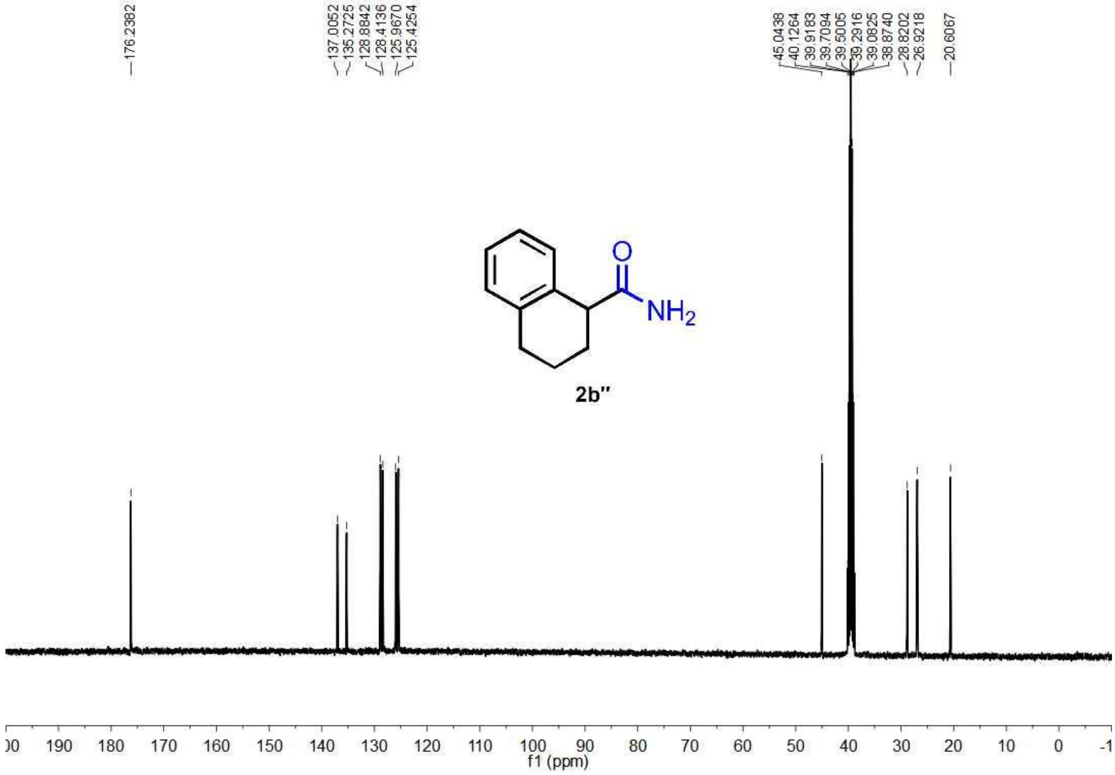

^{1}H NMR of product 3a in $CDCl_3$ (400 MHz)

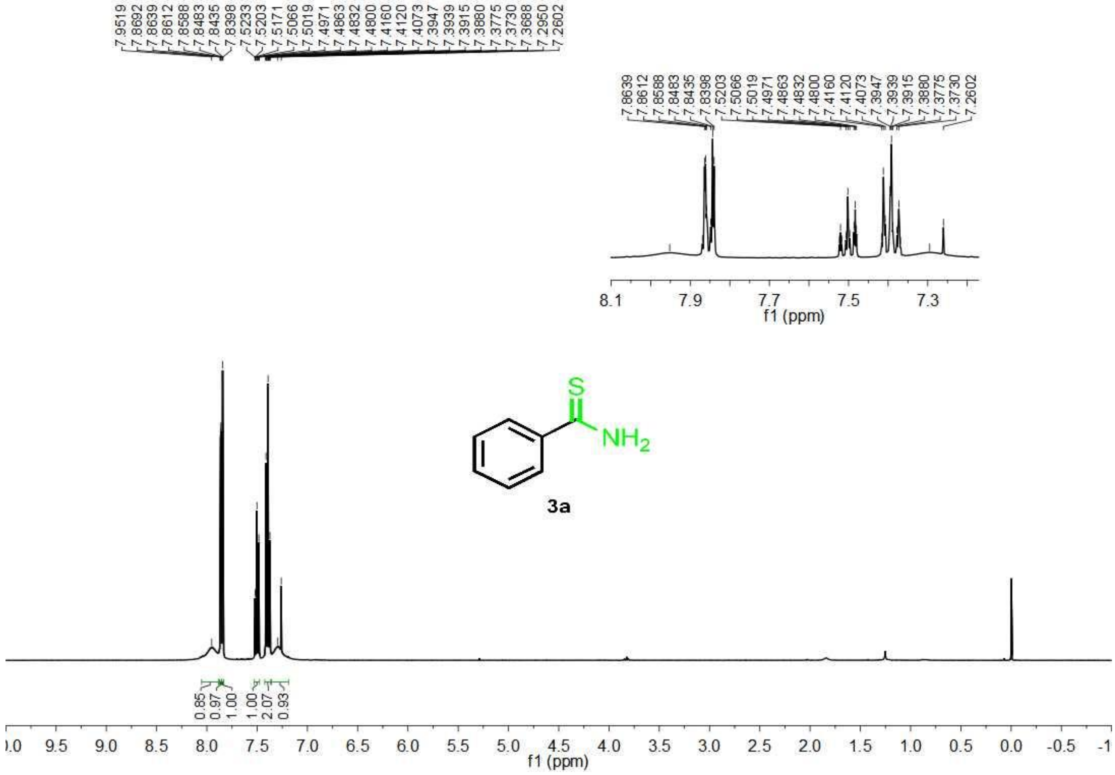

^{13}C NMR of product 3a in $CDCl_3$ (100 MHz)

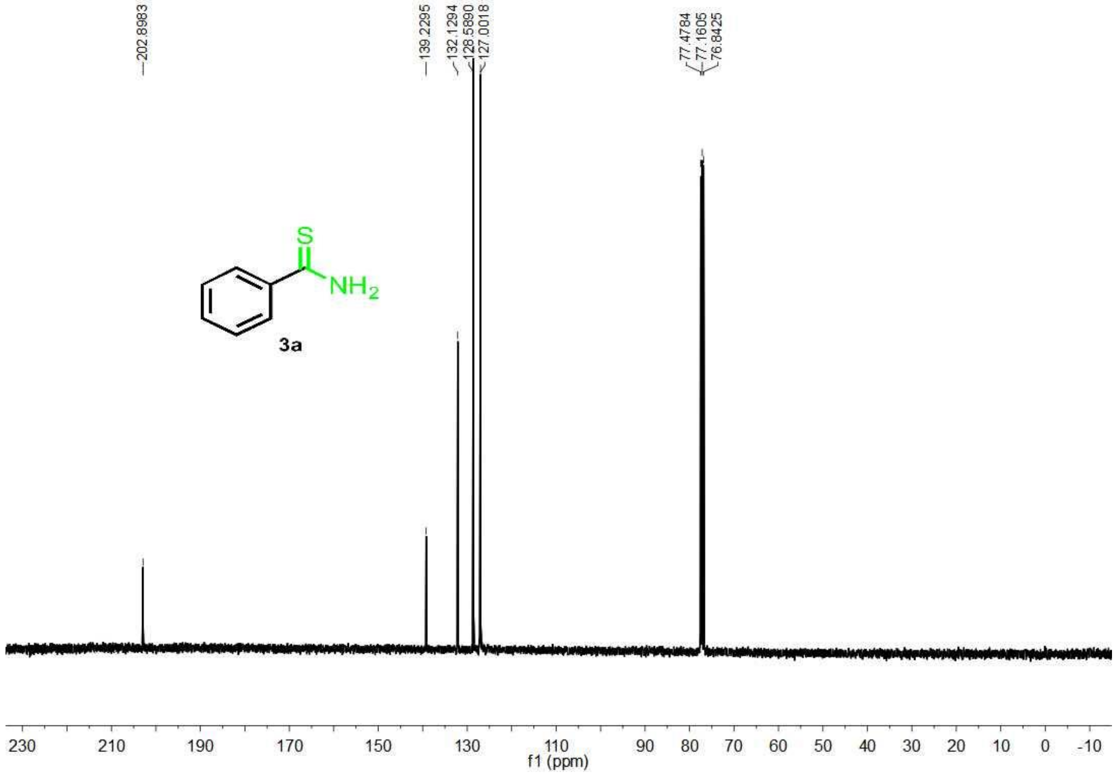

^{1}H NMR of product 3c in $CDCl_3$ (400 MHz)

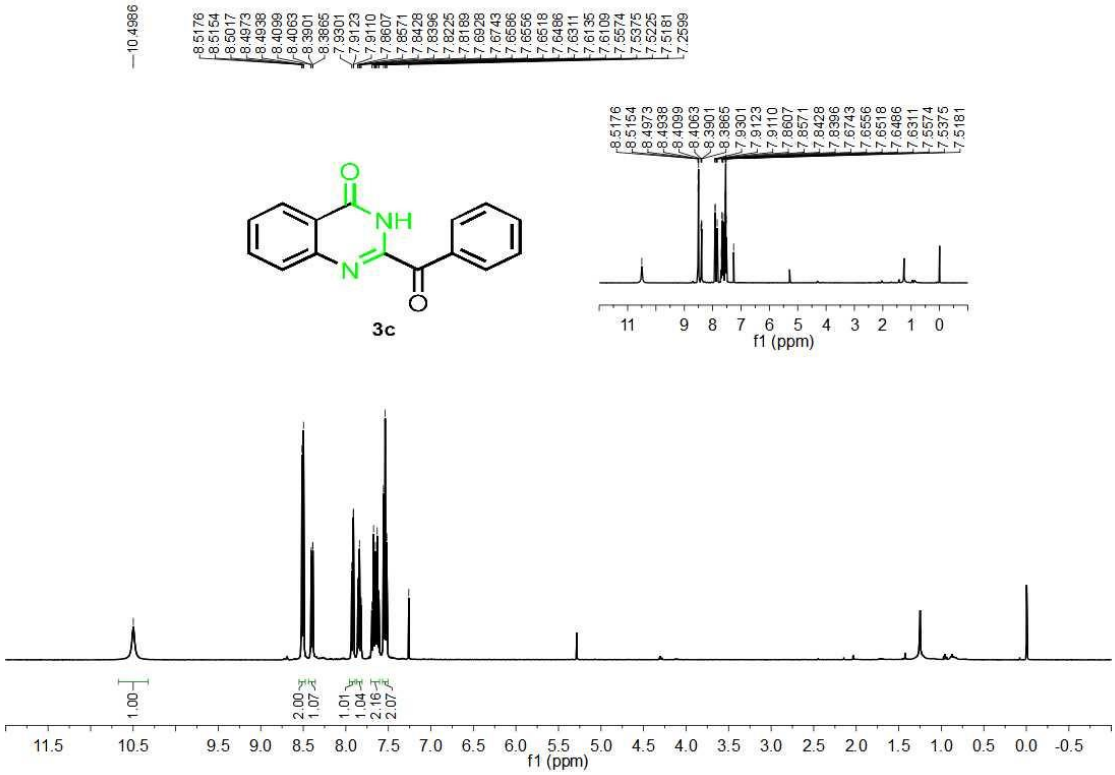

^{13}C NMR of product 3c in $CDCl_3$ (100 MHz)

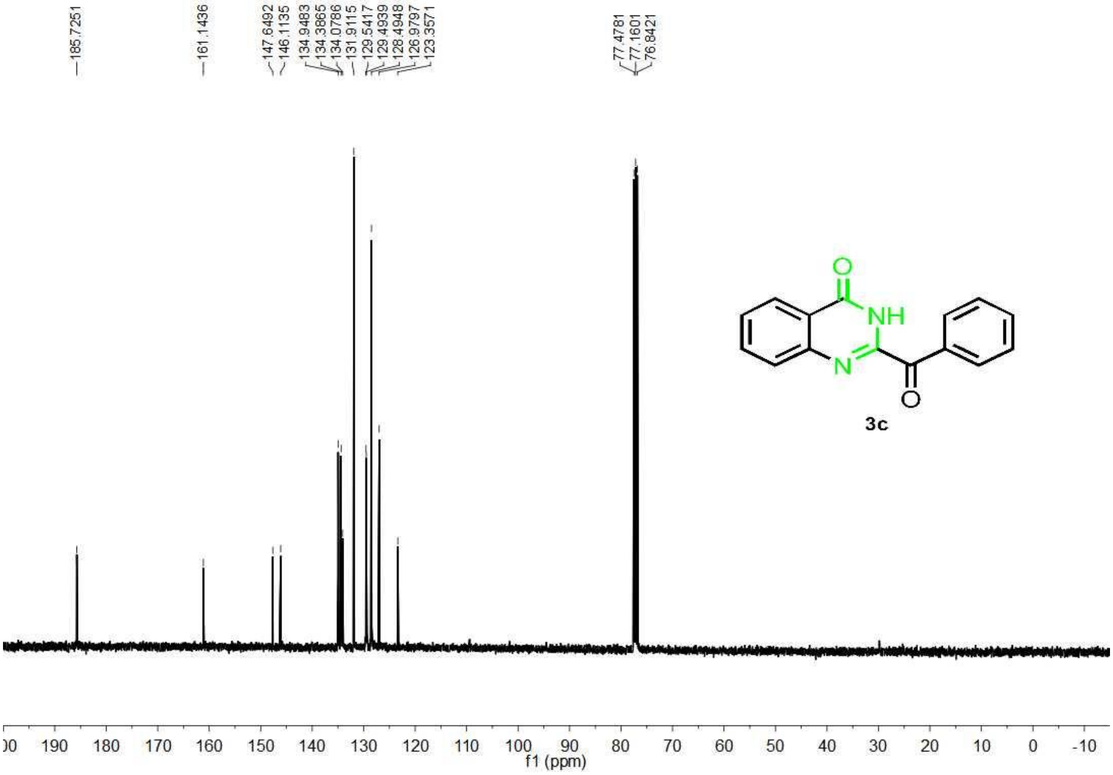

Supplement: Supplementary file 1 [file molecules-24-03838-s001.pdf]
